# Ex-ante impact of pest des petits ruminant control on micro and macro socioeconomic indicators in Senegal: A system dynamics modelling approach

**DOI:** 10.1371/journal.pone.0287386

**Published:** 2023-07-05

**Authors:** Joshua Aboah, Andrea Apolloni, Raphaël Duboz, Barbara Wieland, Pacem Kotchofa, Edward Okoth, Michel Dione

**Affiliations:** 1 International Livestock Research Institute, West Africa Regional Office, Dakar, Senegal; 2 Commonwealth Scientific and Industrial Research Organisation, Agriculture & Food Business Unit, Brisbane, Australia; 3 CIRAD, UMR ASTRE Montpellier, Montpellier, France; 4 Institute of Virology and Immunology, Mittelhäusern, Switzerland; 5 Department of Infectious Diseases and Pathobiology, Vetsuisse Faculty, University of Bern, Bern, Switzerland; 6 International Water Management Institute, C/o ILRI 2R87+GPC, Addis Ababa, Ethiopia; University of Zambia School of Veterinary Medicine, ZAMBIA

## Abstract

Vaccination is considered as the main tool for the Global Control and Eradication Strategy for *peste des petits ruminants (PPR)*, and the efficacity of the PPR-vaccine in conferring long-life immunity has been established. Despite this, previous studies asserted that vaccination can be expensive and consequently, the effectiveness of disease control may not necessarily translate to overall profit for farmers. Also, the consequences of PPR control on socioeconomic indicators like food and nutrition security at a macro-national level have not been explored thoroughly. Therefore, this study seeks to assess *ex-an*te the impact of PPR control strategies on farm-level profitability and the socioeconomic consequences concerning food and nutrition security at a national level in Senegal. A bi-level system dynamics model, compartmentalised into five modules consisting of integrated production-epidemiological, economics, disease control, marketing, and policy modules, was developed with the STELLA Architect software, validated, and simulated for 30 years at a weekly timestep. The model was parameterised with data from household surveys from pastoral areas in Northern Senegal and relevant existing data. Nine vaccination scenarios were examined considering different vaccination parameters (vaccination coverage, vaccine wastage, and the provision of government subsidies). The findings indicate that compared to a no-vaccination scenario, all the vaccination scenarios for both 26.5% (actual vaccination coverage) and 70% (expected vaccination coverage) resulted in statistically significant differences in the gross margin earnings and the potential per capita consumption for the supply of mutton and goat meat. At the prevailing vaccination coverage (with or without the provision of government subsidies), farm households will earn an average gross margin of $69.43 (annually) more than without vaccination, and the average per capita consumption for mutton and goat meat will increase by 1.13kg/person/year. When the vaccination coverage is increased to the prescribed threshold for PPR eradication (i.e., 70%), with or without the provision of government subsidies, the average gross margin earnings would be $72.23 annually and the per capita consumption will increase by 1.23kg/person/year compared to the baseline (without vaccination). This study’s findings offer an empirical justification for a sustainable approach to PPR eradication. The information on the socioeconomic benefits of vaccination can be promoted via sensitization campaigns to stimulate farmers’ uptake of the practice. This study can inform investment in PPR control.

## 1 Introduction

*Peste des petits ruminant* (PPR) is a highly contagious and deadly disease affecting mostly domestic and wild small ruminants. The disease is prevalent across Africa, the Middle East, and South Asia [1]. Due to the high mortality rate, the disease has an important influence on the economies of households. PPR control safeguards livelihoods and contributes to poverty alleviation of rural farm households, especially in developing countries [2]. Due to the devastating socioeconomic losses attributed to the PPR virus, the World Organisation for Animal Health (WOAH) and Food and Agriculture Organization (FAO) have launched the Peste des Petits Ruminant Global Eradication Programme (PPR-GEP) with the aim to eradicate PPR by 2030 [3].

Vaccination is deemed cost-effective and is considered as the key tool to control PPR [4, 5]. Depending on the context, mass or targeted vaccination is required [4, 6]. In any case, logistical support like cold chains is crucial for effective PPR vaccination campaigns [7, 8]. In addition, socio-economic factors that affect willingness to vaccinate or access to vaccines can jeopardize their success. Yet, vaccination campaigns can be expensive [9]. This assertion is reiterated in a study [10] that the effectiveness of disease control may not necessarily translate to overall profit for farmers. Hence, it is imperative to assess *ex-ante* the impact of different vaccination schemes to enable policymakers to know beforehand the costs and benefits of implementing different vaccination strategies.

Various studies have evaluated the adequacy of vaccine campaigns concerning vaccination coverage and a few outbreaks, and the financial viability of different PPR vaccination scenarios at macro-national levels [11, 12]. Studies that have assessed the economic or financial impact of PPR control strategies have used linear estimation [5, 9, 12]. Notable examples are the Vaccicost [9] and the use of social accounting matrix [12]. These estimations of impact ignored the potential recurring feedback interactions that the implementation of PPR control could have on production activities at the farm level and other macro socioeconomic impacts like food and nutrition security.

System dynamics (SD) modelling is a valuable analytical approach to capture the consequential impact of PPR control beyond the farm level. However, the application of SD models in PPR control studies has been limited so far [13]. Thus, adopting the system dynamics modelling approach, this study aims to examine the *ex-an*te impact of PPR control strategies on farm level profitability and the socioeconomic consequences concerning food and nutrition security at national level in Senegal. This objective is achieved by estimating the efficacy of different vaccine delivery scenarios based on the farm households’ profitability levels and the impact on the potential per capita average consumption of sheep and goat meat in Senegal.

## 2 Materials and methods

**Fig 1:**
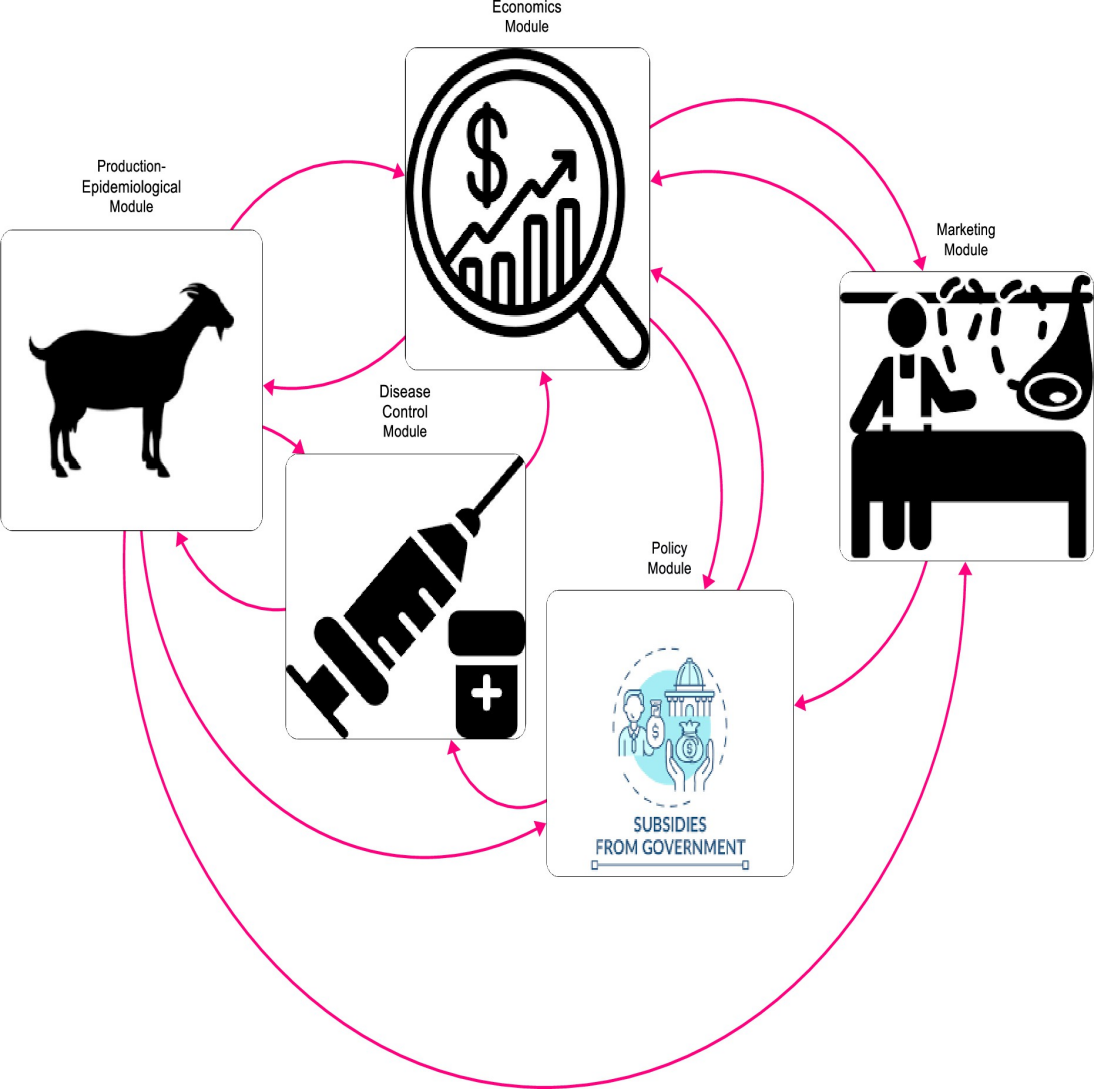
Overview of module interactions in the model.

A bi-level system dynamics model was compartmentalised into five modules. Three modules–the integrated production-epidemiological module, disease control module, and economics module were modelled at the farm level, and the marketing and policy modules were modelled at the national level. The interactions among the five modules are depicted by the pink links in Fig 1 and discussed in the ensuing sub-sections. The model was parameterised with data sourced from household survey data from the ECo-PPR project, and official data from the African Development Bank, World Bank, and FAOSTAT. The model was developed with the STELLA Architect software.

### 2.1 Farm level modules

**Fig 2:**
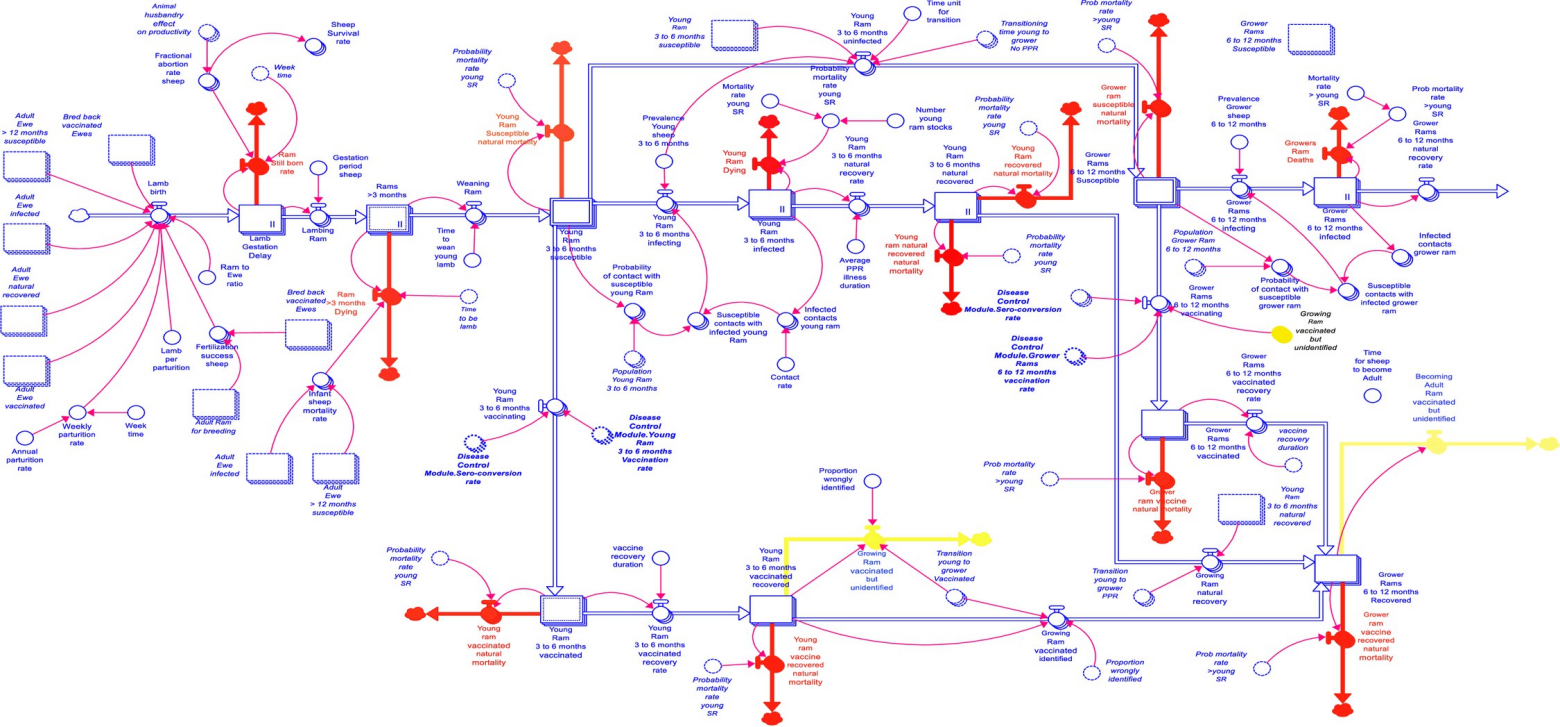
An extract of the SFDs showing the vaccination pathway for young ewes.

The first farm level module is the integrated production-epidemiological module, which was organised into two sectors (i.e., sheep and goats) and disaggregated based on the animal species to highlight the parturitional differences [14]. Adopting the animal categorisation [4], the model considered that small ruminants transitioned through four growth stages juvenile stage (0–3 months), young animals (>3 to 6 months), growing animals (>6 to 12 months), and adult animals (>12 months) in five different infection statuses–susceptible, infected, vaccinated, recovered, and dead. Fig 2 shows an extracted stock and flow diagram of the transition of young rams to grower rams in the integrated production-epidemiological module.

In the baseline scenario, susceptible animals became infected based on the contact rate and the prevalence (i.e., the number of already infected individuals in the herd) [4]. Infected animals recover or die after 10 days. Animals that recover from vaccination or recover naturally (without vaccination) acquire life-long immunity. Moreover, the demographic dynamics consider the transition of animals from one growth stage to the next within each compartment. The primary inflows in the production-epidemiological module consist of new births and animals received as gifts. An estimated abortion rate of 0.52 (from the household survey) was specified in the model^.^ Moreover, new births from recovered (or vaccinated) mothers, are covered by maternal antibodies for the first 3–4 months of life, before becoming susceptible.

**Fig 3:**
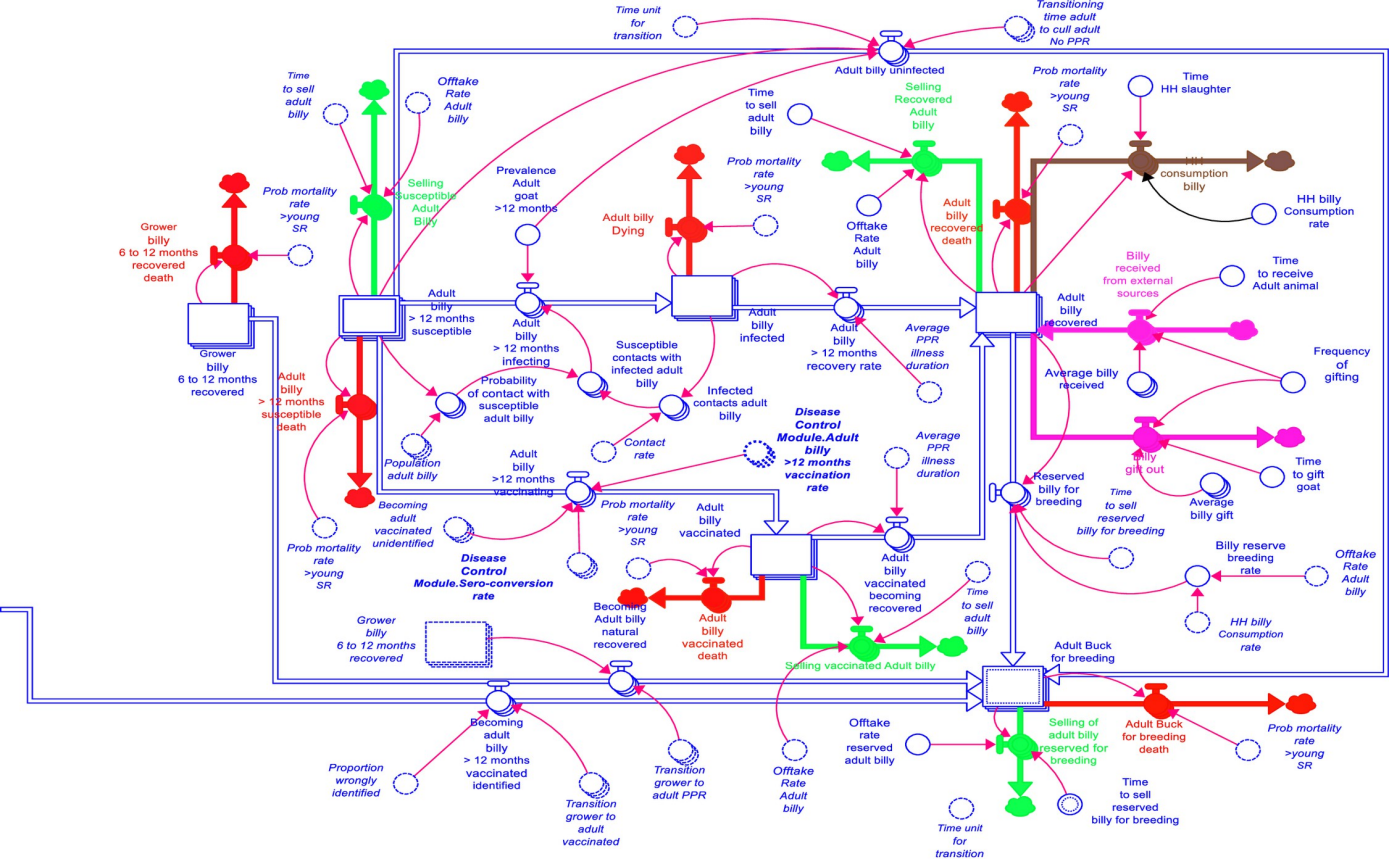
An extract of the SFD showing the outflows.

A fraction of susceptible animals is vaccinated based on the quantity of vaccine available and the time vaccines are deployed. In the absence of proper identification measures, animals may receive multiple vaccinations, resulting in what is referred to as vaccination wastage (highlighted as yellow-coloured outflows in Fig 2). Consequently, vaccine wastage quantifies the repeated vaccinations of individual animals. Fig 3 depicts five distinct outflows: death (red-coloured outflow), sales (green-coloured outflow), animals given as gifts (purple-coloured outflow), theft (black-coloured outflow), and household consumption as an outflow from the adult animal transition stage.

**Fig 4:**
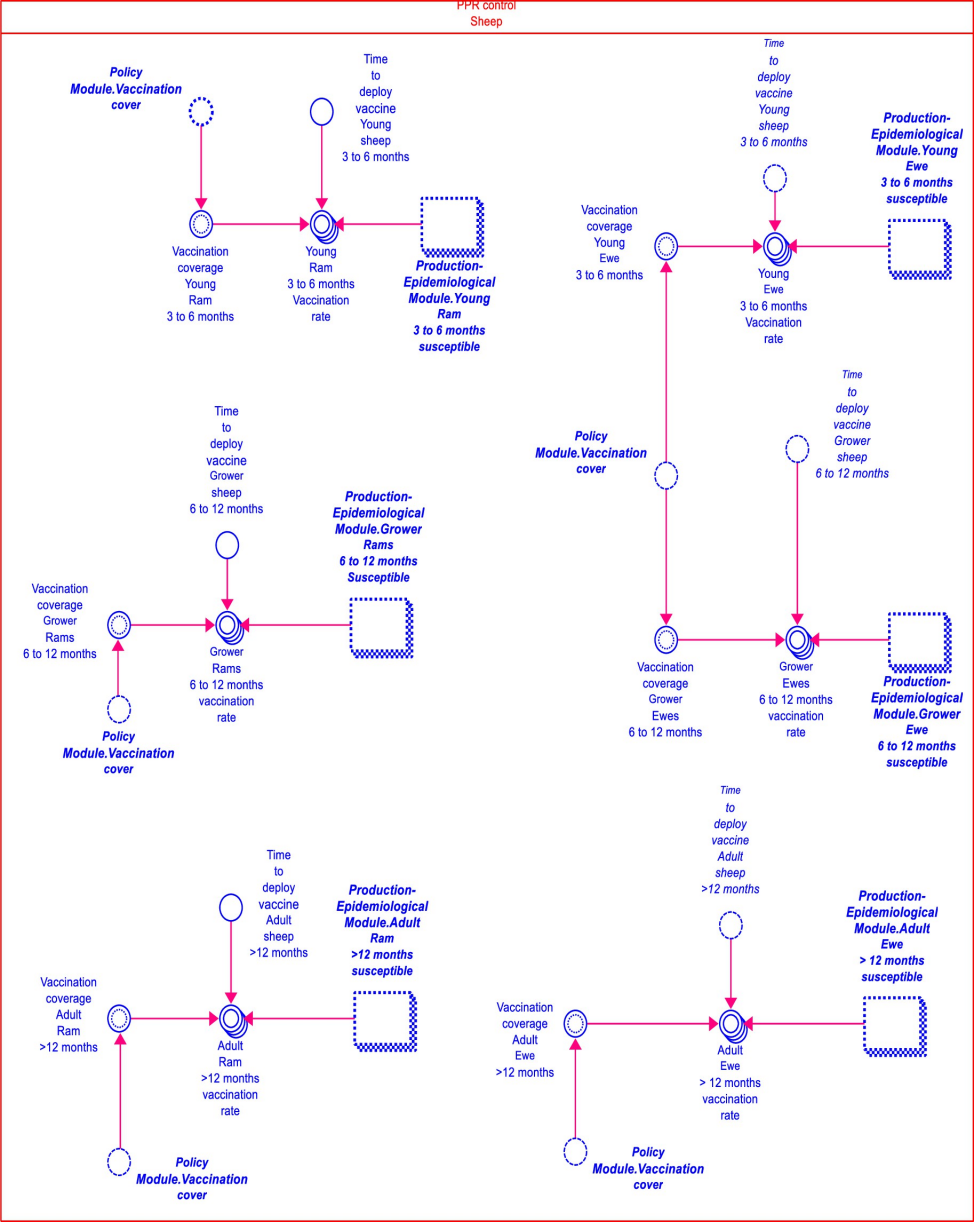
The PPR for sheep sector in the disease control module.

The second farm level module is the disease control module. Fig 4 shows the PPR control for sheep sector of the disease control module. In this module, vaccination was the earmarked disease control mechanism. The estimated vaccination rate from this module influenced the number of susceptible that are vaccinated in the production module. Vaccination in production year_(y+1)_ was influenced by the household decision to invest, which was captured as an expected production investment determined based on profitability in year_(y)_ [6]. No vaccination coverage was assumed for the baseline. The unit vaccination cost of 119 FCFA ($0.19) per animal (sheep or goat) from an official (government) source was factored into the total vaccination cost. The unit vaccination cost comprised the following costs: vaccine per dose, injection supplies, personnel cost, transport, training, social mobilisation, and surveillance and monitoring costs [9].

The economics module is the third farm level module that highlights the financial implication of production decisions. The number of small ruminants sold, which is an outflow from the production-epidemiological module, becomes an input for the total revenue estimations in the economic module. The body weights of the sold animals are influenced by the feed intake, which was predicated on the expected farm household production investment. The unit price of animals sold was endogenised in the marketing module. Hence, price dynamics were influenced by the market forces of aggregate supply and demand. The total production costs captured in the economic module included the total feeding and disease control costs. The gross margin over time was a key output estimated in the economic module. The gross margin was estimated as the difference between the total revenues and production costs. The gross margin level for year_(y)_ was used as an indicator for production investment in year_(y+1)_. Therefore, the production investment was specified as the first-order exponential smoothing of the gross margin for year_(y)_. A summary of the key parameters in the farm-level modules is presented in Table 1. Detailed equations of all parameters are included in S2 File.

**Table 1:** A summary of key parameters in the farm-level modules.

Parameter	Module	Value	Source
Vaccination cost	Economics	119 FCFA ($0.19) per vaccine delivered	Official government data
Daily weight gained by animals	Economics	62g per day	FAOSTAT
Prevalence rate	Production	0.25	[4]
Contact rate	Production	1	Assumed
Offtake rate for young ewe	Production	0.2	ECo-PPR household survey
Gestation period	Production	37.5 weeks	[14]
Average PPR illness duration	Production	10 days	[15]
Time to keep ewes		104 weeks	[16]
Time to sell bred-back nannies	Production	208 weeks	[16]
USD-CFA exchange rate	Economics	0.0015	Xe.com
Fractional abortion rate	Production	0.32	ECo-PPR household survey
Ram to Ewe birth ratio	Production	0.5	ECo-PPR household survey
Household consumption rate	Production	0.2	ECo-PPR household survey
Average feeding cost	Economics	2000 FCFA ($0.32) per animal per week	Market data
Feeding ration	Economics	1 bag for 10 animals (wet season) & 5 (dry season)	Market data
Proportion of multiple vaccinations	Disease control	0	No markings practised
Parturition per small ruminant	Production	1	No twining assumed

### 2.2 National level modules

**Fig 5:**
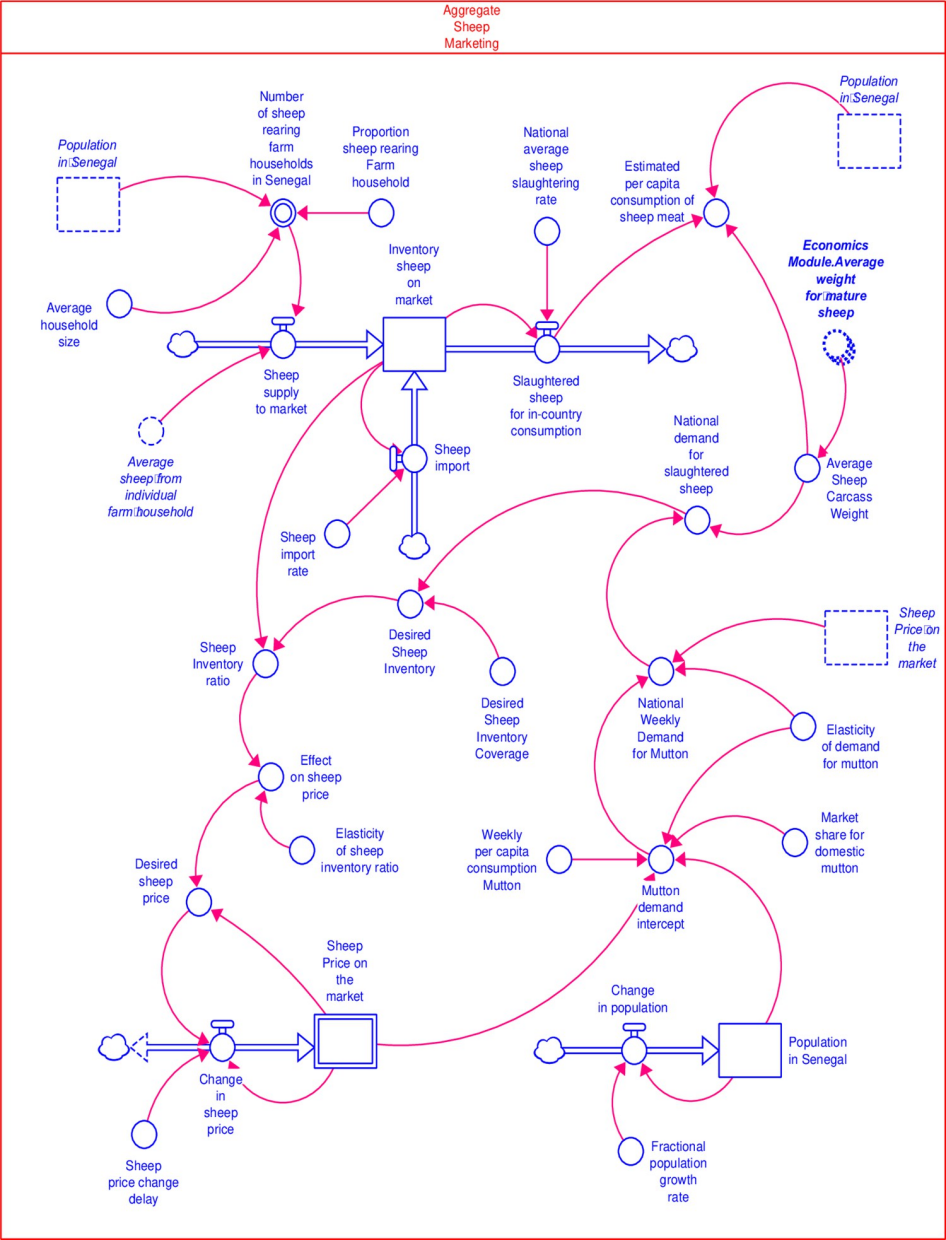
The aggregated sheep marketing sector in the marketing module.

The marketing module is one of the national level modules and highlights the consequence of farm level interventions on the supply of sheep and goats on the market, and the feedback effect of demand and price on production decisions. Fig 5 shows the stock and flow diagrams in the aggregated sheep marketing sector within the marketing module. The small ruminant supply on the market was extrapolated based on the number of small ruminants in the integrated production- epidemiological module, the population of farm households, and the number of farm households selling at monthly offtake rates. Livestock farming is practised by 29.5% of the total Senegalese households [17], and the average household size was seven [18].

The total supply from in-country producers and the in-flows of small ruminant imports accumulates into the inventory of small ruminants on the markets. The outflow from the stock of small ruminant inventories was specified by applying the national production, estimated based on data from African Development Bank (from 2010 to 2020). The aggregate demand for the small ruminant was determined using the average carcass weight specified from the economic module and estimated weekly demand for meat [19] using a 0.61 elasticity of demand [20]. Fractional population growth of 2.7% was applied in the model to capture the population dynamics. From 2010 to 2018, the annual population growth in Senegal is 2.7%, and the total population size as of 2021 is 17,196,308 (https://data.worldbank.org/country/SN).

The unit prices for goats and sheep were endogenised [21] based on the aggregate demand and supply for small ruminants. The unit prices of sheep and goat meat were determined using a biennial price change and an estimated 0.01 price elasticity of supply. The average producer price for goat and sheep from 2010 to 2019 are 1061.21 per kg and 1,702.94, respectively.

The potential per capita consumption of mutton and goat meat (PC _[consume]_) is a model output in the marketing module. PC _[consume]_ was estimated as the sum of national-level consumption from total slaughtered animals at the national level (Consume _[national]_), the aggregated household consumption based on the product of the average household consumption at the farm level (HH _[consume]_), and the number of small ruminant rearing households (HH _[farm]_), divided by the proportion of the non-infant population (Pop _[non-infant]_). Mathematically, PC _[consume]_ is expressed as shown in Eq 1.


$$PC_{\left[consume\right]}=[Consume_{\left[national\right]}+(HH_{\left[consume\right]}*HH_{\left[farm\right]})]/(Pop_{\left[Senegal\right]}*Pop_{\left[non‐infant\right]})$$ (1)

The second national level module is the policy module. This module captured the impact of the provision of government subsidies for PPR control. The provision of government subsidies influenced the unit vaccination cost in the economic module. The full cost of vaccination was borne by households when no subsidies were provided. Consequently, the provisions of the government’s subsidies reduced the unit vaccine cost in the economics module. For the baseline, no government subsidies were applied. Also, the total government subsidy provided was estimated based on an extrapolated vaccination cost using the average number of small ruminants produced at the household level for all livestock-producing households in Senegal.

### 2.3 Model validation

Following the SD model validation sequence [22], the model structure was first validated for the unit consistency test. The transition of animal growth was partitioned to suit the biological growth progression of the animals highlighted in previous studies. Also, the interrelated feedback and integration of the epidemiological and production parameters of the model were structurally validated by a reference group of experts including veterinaries and epidemiologists.

**Fig 6:**
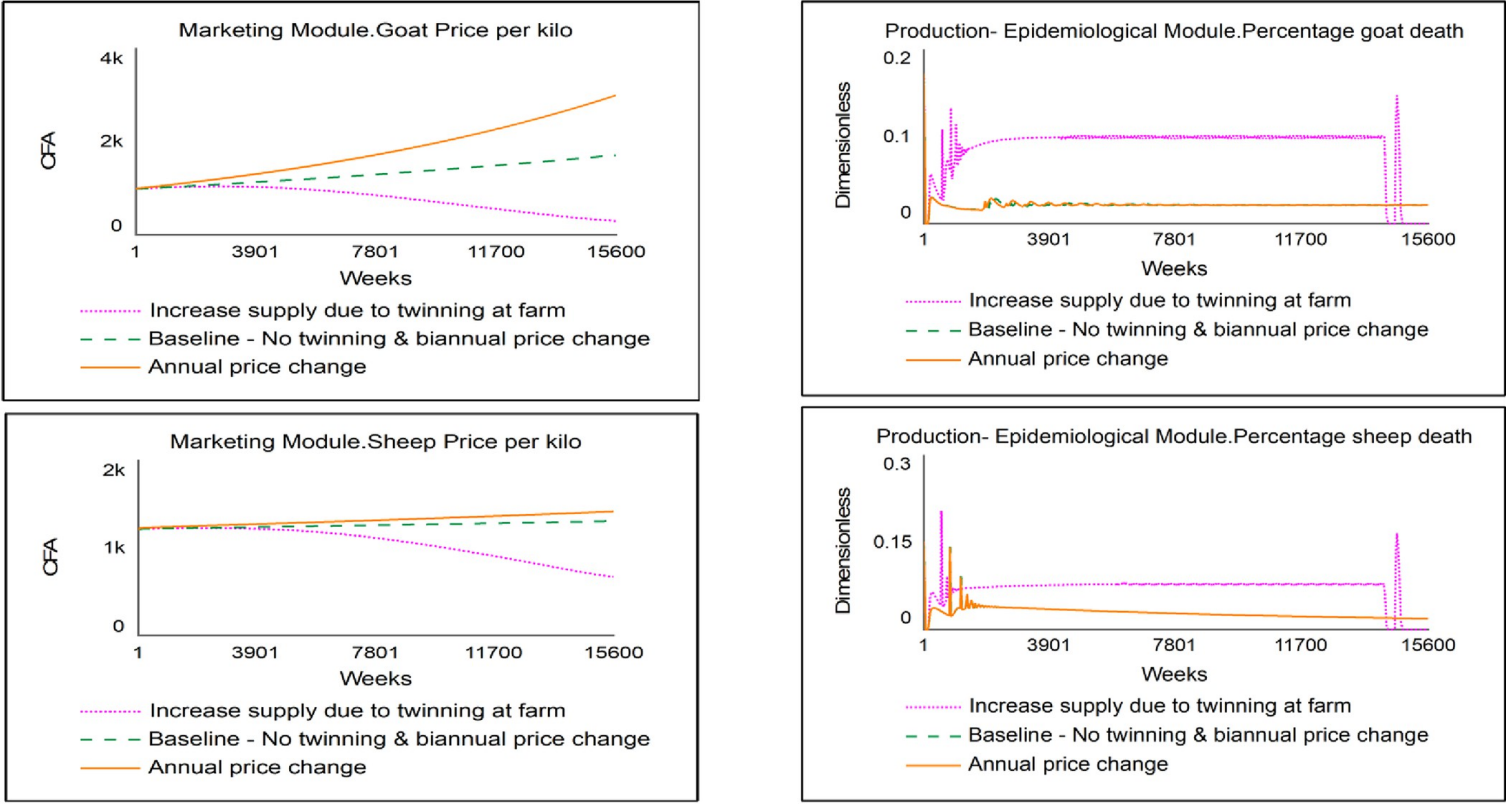
Results of the production-related extreme condition tests.

**Fig 7:**
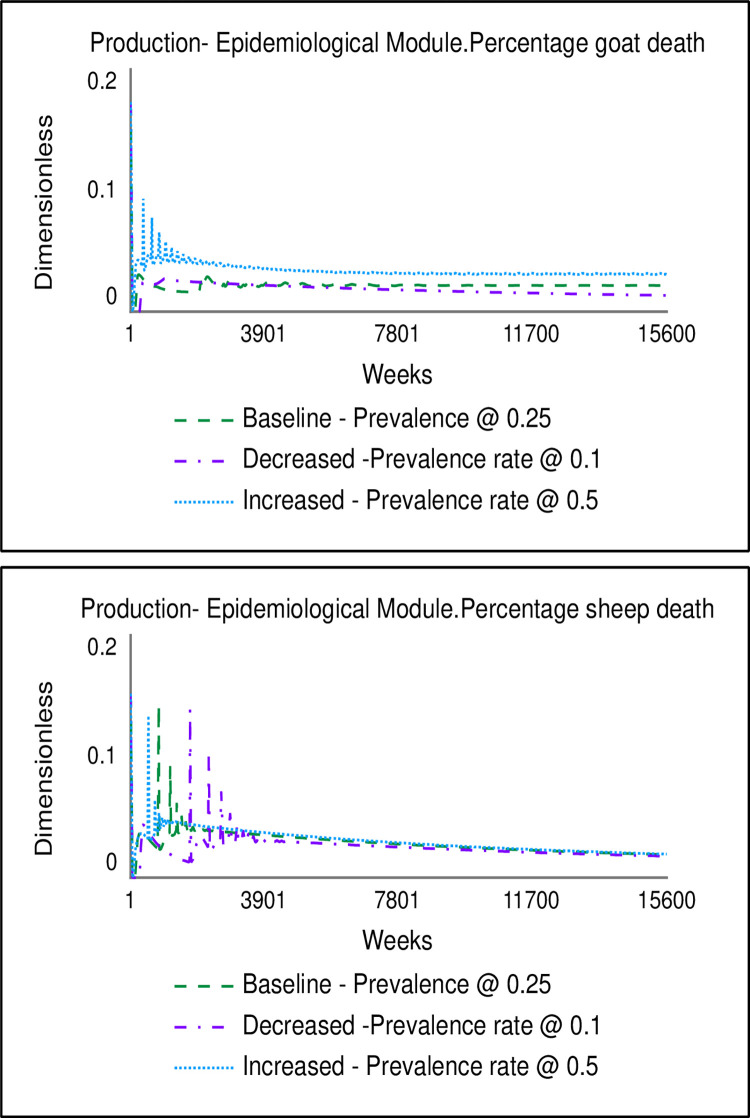
Results of the economic-related extreme condition tests.

The model behaviour was validated using production-related, epidemiological, and economic extreme-condition tests. Figs 6 and 7 show the key variables (unit price of animals on the left section (top and down), and the percentage of mortality on the right section) for the production-related and economics extreme-conditioned tests, respectively. The production-related extreme condition test was performed by assuming twinning births for both sheep and goats. This condition was expected to increase production at the farm level and simultaneously cause the aggregate supply to increase. Consistent with neoclassical economics theory, the price of goats and sheep decreased when twinning occurred at the farm level due to the increased supply resulting from increased production. Also, an increase in the number of goats and sheep at the farm level due to twinning corresponded to an increase in animal deaths when the prevalence rates of PPR remained unchanged.

**Fig 8:**
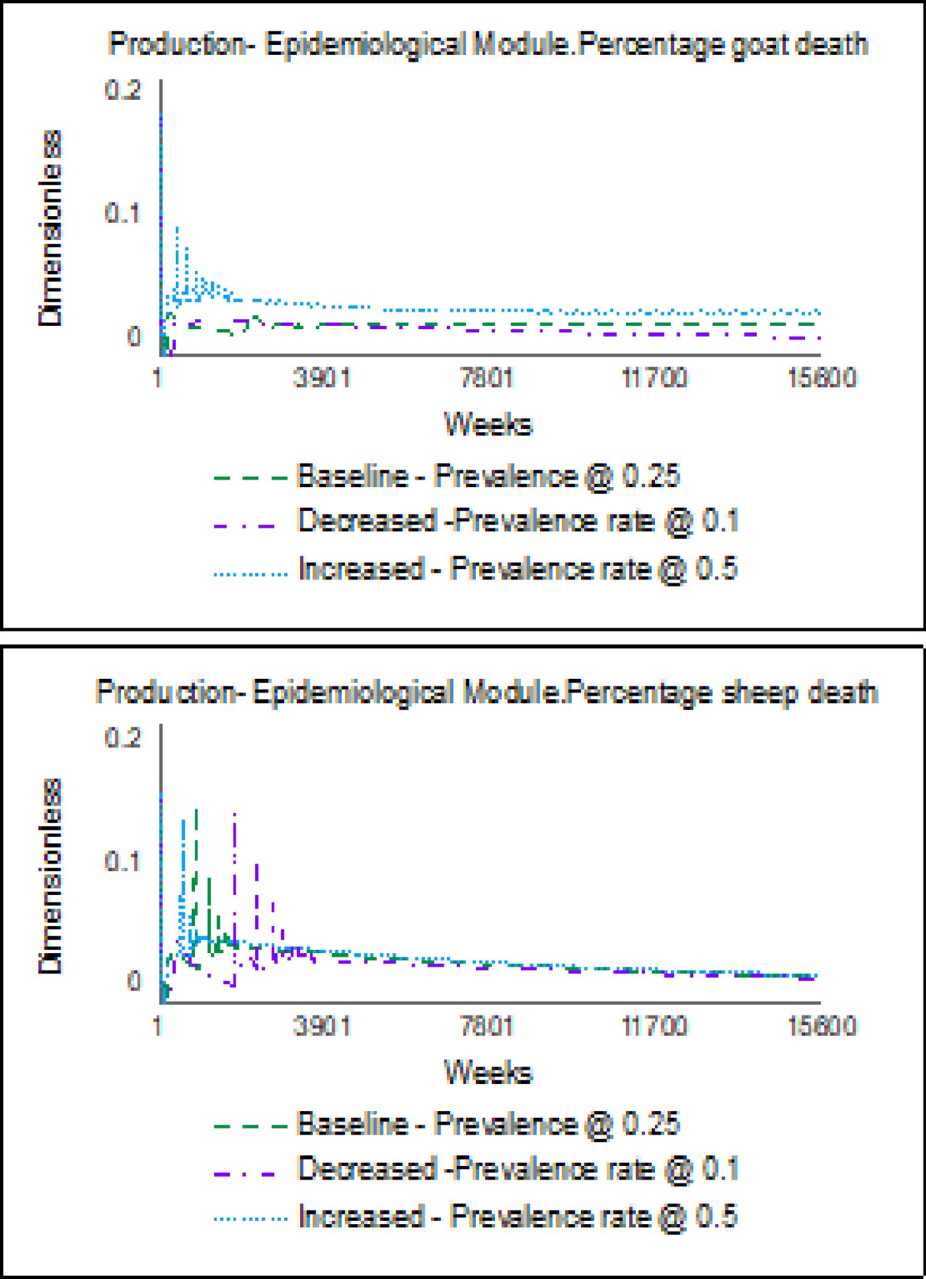
Results of the epidemiological-related extreme condition tests.

For the economics-related extreme-condition test, the biennial price change delay in the baseline was altered to an annual price change delay. This condition caused the unit price for goats and sheep to increase compared to the baseline. For the epidemiological-related extreme condition test, the prevalence rate for PPR was altered from the baseline level of 0.25 [4] by decreasing and increasing it to 0.1 and 0.5, respectively. The model behaviour for these extreme-condition tests, shown in Fig 8, was consistent with expected real-life behaviour. An increase and decrease in the prevalence rate caused the percentage of mortality to rise and decline, respectively.

### 2.4 Sensitivity analysis

**Fig 9:**
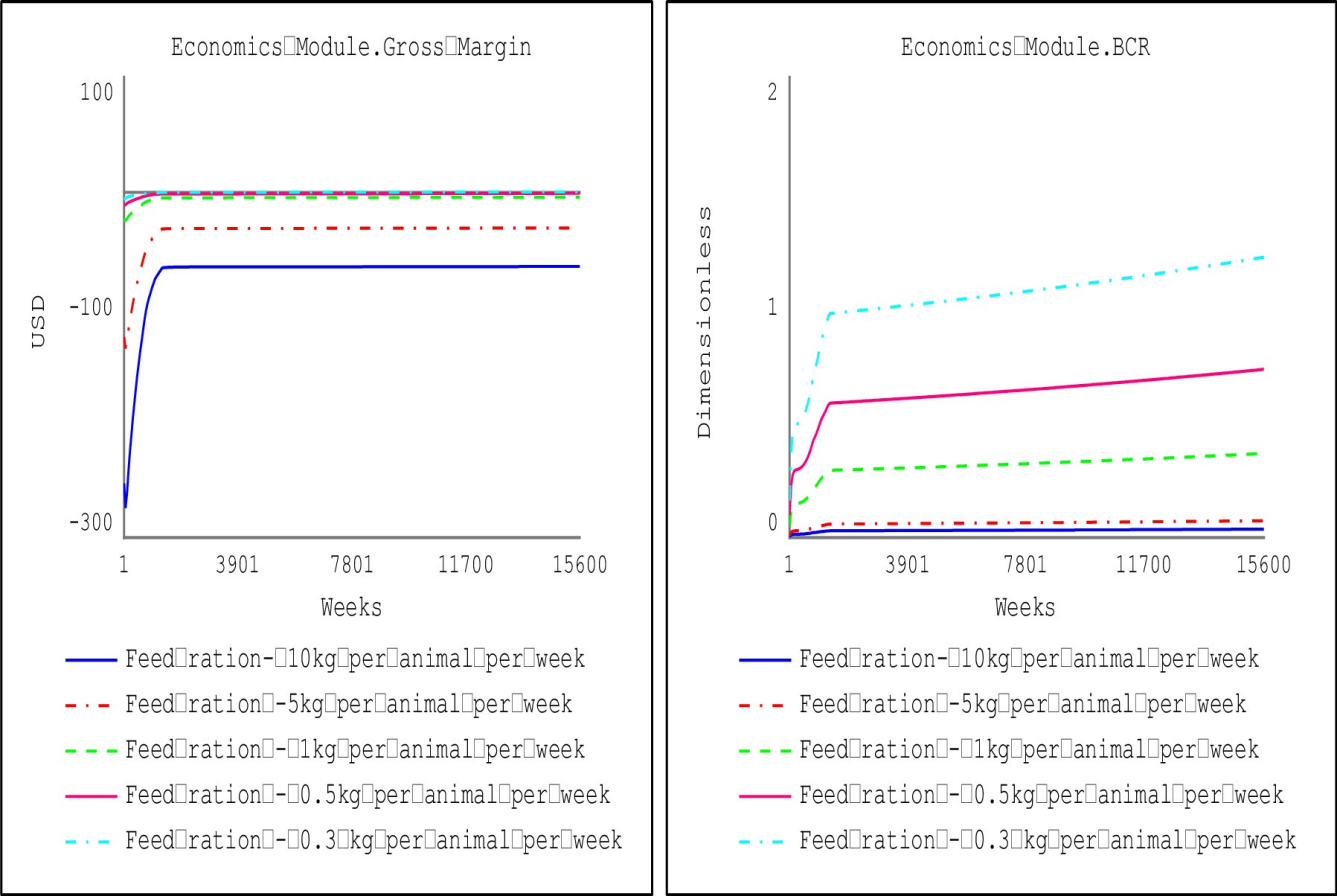
Threshold of economically viable feeding ration at price of animal feed (dry season).

Sensitivity analysis focused on the feeding cost because it is the most significant component of the production cost. At the prescribed feeding rations for the dry season (one bag of 50kg for 10 small ruminants) and wet seasons (a 50kg bag of feed for 5 animals), the results in Fig 9 indicate that small ruminant production will not be economically viable, as evidenced by benefit-cost ratio (BCR) of less than 1. Hence, sensitivity analyses were performed to determine an economically viable threshold for feeding rations during the dry and wet seasons. Results of the sensitivity analyses show that small ruminant production would be an economically viable venture (BCR greater than 1) if the weekly feeding ration for purchased feeds during the dry season is below the feeding ration threshold of 0.3 kg per animal. Consequently, farm households may adopt alternative feeding sources like residues and freely assessed forages to supplement the purchased feeds.

**Fig 10:**
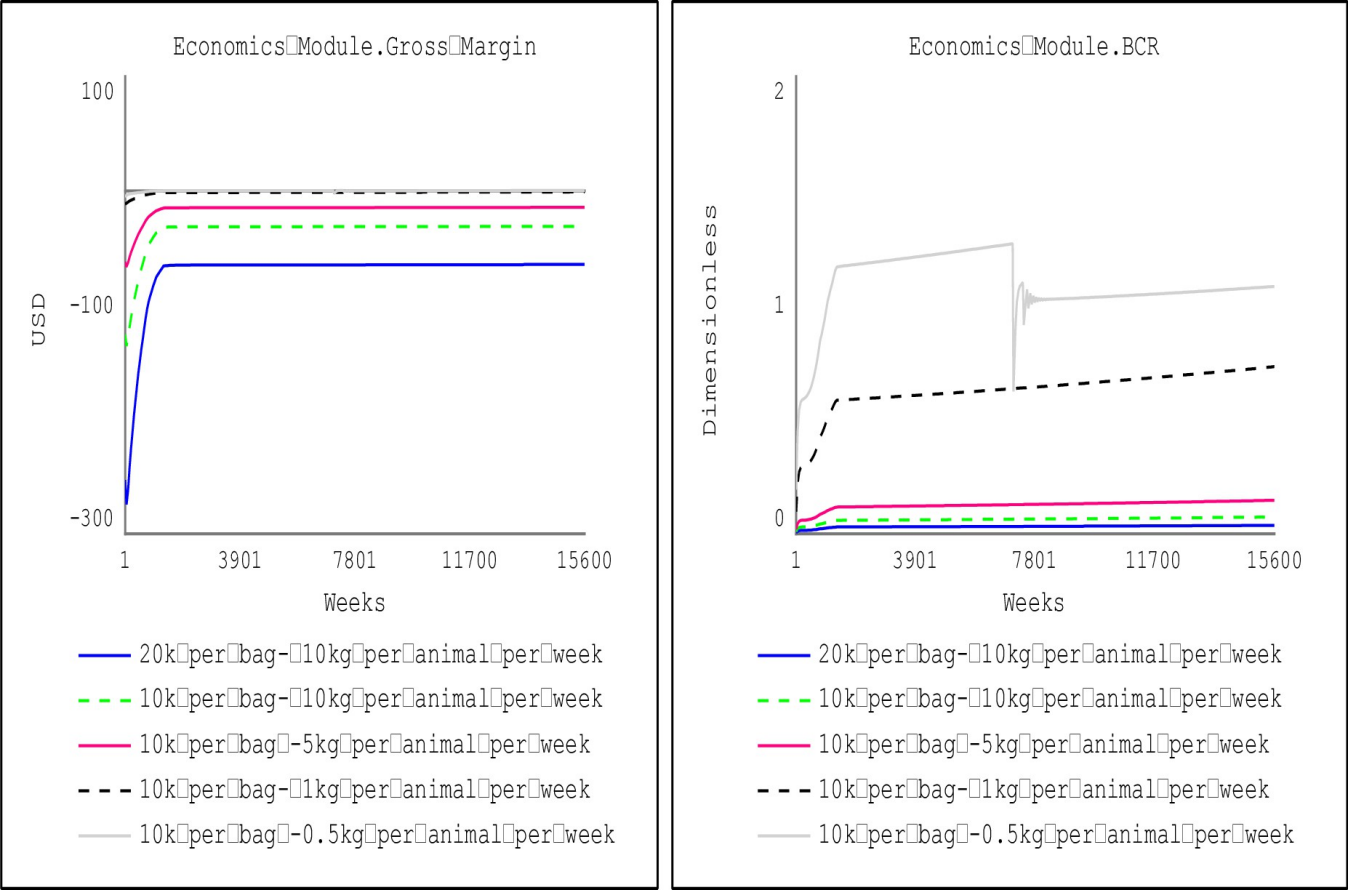
Threshold of economically viable feeding ration at the price of animal feed (wet season).

**Fig 11:**
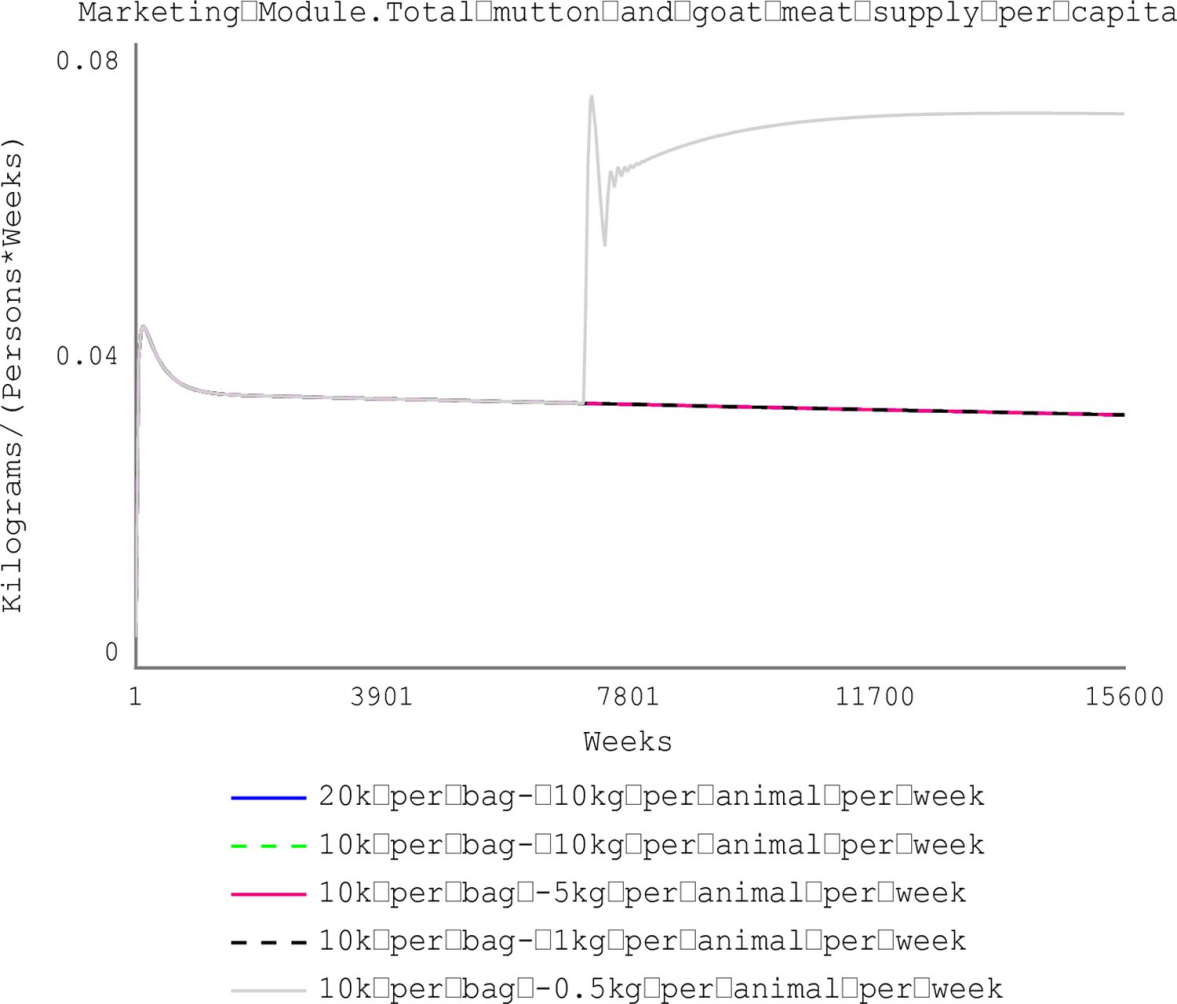
Weekly per capita consumption of goat meat and mutton.

Fig 10 shows farm households keeping small ruminants can increase the weekly feeding ration purchased by 67% (i.e., from 0.3 kg/animal to 0.5 kg/animal) during the wet season when the price of feeds falls by 50%. At the aggregate national level, the weekly per capita consumption for mutton and goat meat increases by 58.82% (from 0.034 kg/week/person) when small ruminant production is economically viable (at a feed price of 10,000 FCFA ($16.25) and weekly feeding ration is 0.5 kg/animal (as shown in Fig 11).

### 2.5 Vaccination scenarios

Three dimensions of vaccination are used to formulate the vaccination scenarios in this study. These include vaccination coverage, vaccine wastage, and the provision of government subsidies. For the vaccination coverage, two levels were considered– 26.5% coverage (the current nationwide vaccination coverage as of 2022) and 70% coverage (the recommended threshold for eradication according to the GCES). The vaccination coverage followed the national strategy for PPR eradication, whereby animals older than three months are vaccinated during the first two years, followed by a targeted vaccination of animals between four and 12 months for the following four years.

Two cases of vaccine wastage (no multiple vaccinations of the same animal versus 10% multiple vaccinations) were explored. In line with the vaccination studies conducted in Ghana and Burkina Faso [3], the provision of government subsidy and a case where households bear the full cost of vaccination were examined. All scenarios were compared with a no-vaccination scenario. Table 2 shows a summary of the different combinations of vaccination scenarios examined in the study.

**Table 2:** Vaccination scenarios.

Vaccination scenario	Details of scenarios
Scenario 1	0% coverage at 0% multiple vaccinations without government subsidy (baseline)
Scenario 2	26.5% coverage at 0% multiple vaccinations without government subsidy
Scenario 3	26.5% coverage at 0% multiple vaccinations with government subsidy
Scenario 4	26.5% coverage at 10% multiple vaccinations without government subsidy
Scenario 5	26.5% coverage at 10% multiple vaccinations with government subsidy
Scenario 6	70% coverage at 0% multiple vaccinations without government subsidy
Scenario 7	70% coverage at 0% multiple vaccinations with government subsidy
Scenario 8	70% coverage at 10% multiple vaccinations without government subsidy
Scenario 9	70% coverage at 10% multiple vaccinations with government subsidy

## 3 Results

The validated model was recalibrated to the economically viable threshold for purchased feeding rations and simulated for 30 years using a weekly timestep. Thus, the model captured the seasonal climatic variations in a year by assuming that the time followed a calendar year. The wet season started in week 24 and ended in week 40. The remaining weeks represented the dry season. The economically viable threshold for feeding rations specified in the model for the impact assessment is 0.5kg/animal/week and 0.2kg/animal/week for the wet and dry seasons, respectively.

Table 3 shows a summary of the main model outputs–gross margin at the farm level and the per capita consumption of mutton and goat meat at the national level. For the baseline scenario (i.e., no vaccination), farm households earn on average 7,040.36 FCFA ($11.44) annually at the economically viable threshold for feeding rations. Comparisons of the focal indicators for two cases–*(i)* feed cost at 10000 FCFA ($16.25) for a feeding ration of 1 kg/animal/week and *(ii)* feed cost at 10000 FCFA ($16.25) for a feeding ration of 0.5kg/animal/week show a statistically significant difference in the gross margin and per capita consumption of mutton and goat meat. Considering that natural grazing supplemented with fodder and kitchen leftovers/ residues is a common practice [16], there is a high tendency for farm households to reduce the feeding cost component of the total production cost and consequently, the gross margins.

**Table 3:** Summary of baseline (no vaccination) results.

Feeding rations	Gross margin (USD/week)	Per capita consumption of mutton and goat meat (kg week^-1^person^-1^)
Feed at 20,000 FCFA ($32.5) and feeding ration of 10kg/animal/week	-70.93	0.034
Feed at10000 FCFA ($16.25) and feeding ration of10kg/animal/week	-34.37	0.034
Feed at 10,000 FCFA ($16.25) and feeding ration of 5kg/animal/week	-16.08	0.034
Feed at10000 FCFA ($16.25) and feeding ration of1kg/animal/week	-1.45	0.034
Feed at 10,000 FCFA ($16.25) and feeding ration of 0.5kg/animal/week	0.22	0.054
*Welch two-sample t-test (for Feed at 10000 FCFA & 1kg feeding ration and Feed at 10000CFA & 0*.*5kg feeding ration)*		
T	-150.43	-142.29
Df	21501	15857
p-value	<2.2e-16	<2.2e-16

### 3.1 Impact of vaccination coverage

**Fig 12:**
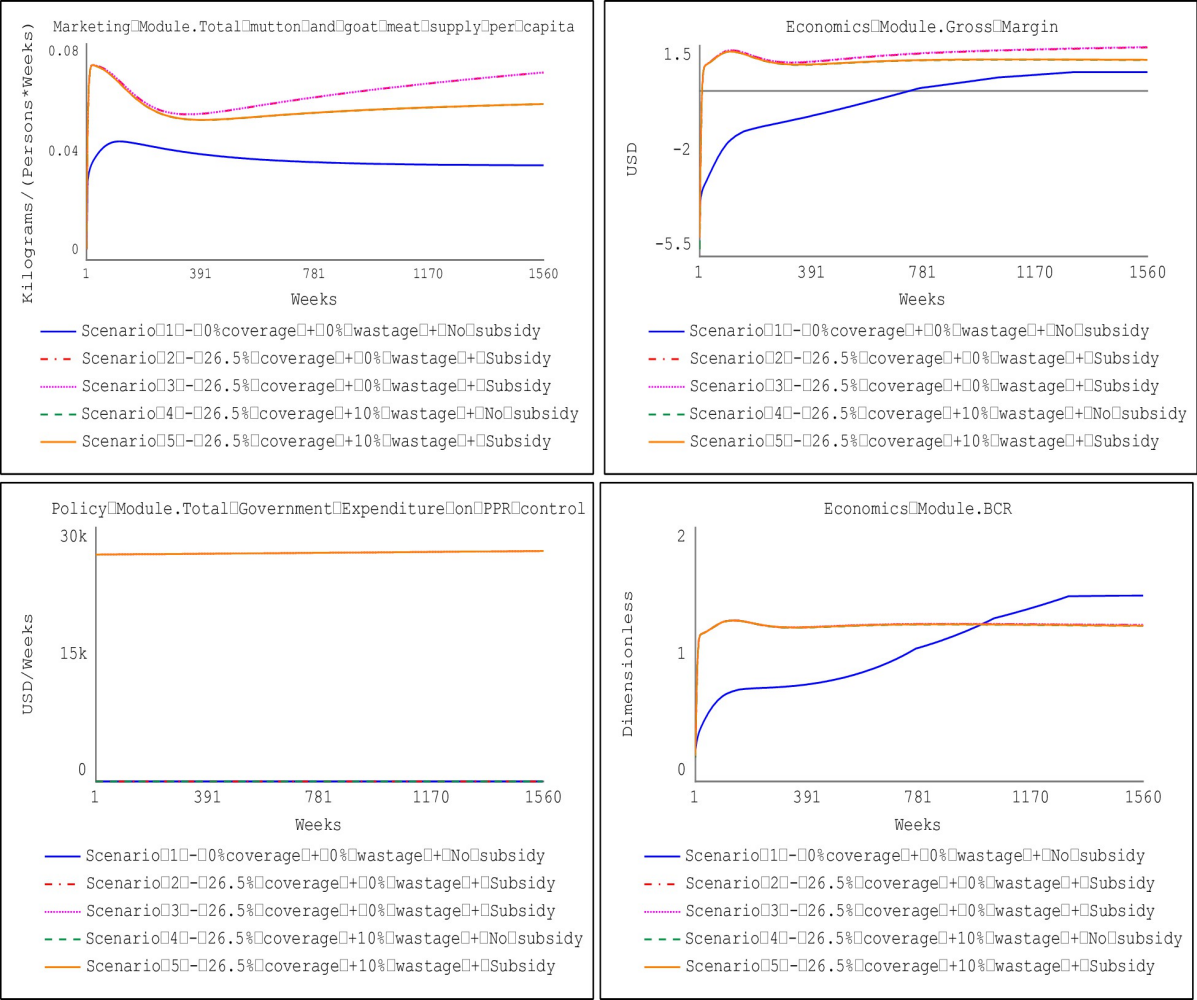
Impact of prevailing vaccination coverage (26.5%).

**Fig 13:**
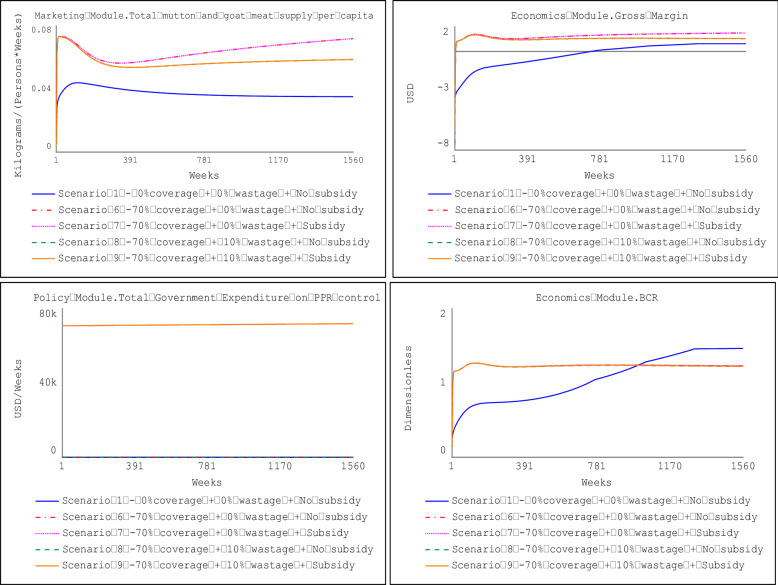
Impact of vaccination coverage (70%) for PPR eradication.

Figs 12 and 13 show the impact of vaccination scenarios under the prevailing coverage of 26.5% and the prescribed vaccination coverage for PPR eradication (i.e., 70%), respectively. Results indicate that, compared to a no-vaccination scenario (i.e., Scenario 1), all the vaccination scenarios yielded statistically significant differences in the gross margin attainable by farm households at the farm level and the potential per capita consumption for supply of mutton and goat meat except for four comparisons of vaccination scenarios as presented in Table 4. Details of the Welch two-sample t-test to compare the different scenarios are presented in the supplementary information.

**Table 4:** Vaccination scenarios with a statistically insignificant difference (for the gross margin).

	Scenario 2 (26.5% coverage at 0% multiple vaccinations without government subsidy) Vs. Scenario 3 (26.5% coverage at 0% multiple vaccinations with a government subsidy)	Scenario 4 (26.5% coverage at 10% multiple vaccinations without government subsidy) Vs. Scenario 5 (26.5% coverage at 10% multiple vaccinations with a government subsidy)	Scenario 6 (70% coverage at 0% multiple vaccinations without government subsidy) Vs. Scenario 7 (70% coverage at 0% multiple vaccinations with a government subsidy)	Scenario 8 (70% coverage at 10% multiple vaccinations without government subsidy) Vs. Scenario 9 (70% coverage at 10% multiple vaccinations with a government subsidy)
Mean annual gross margin (in $/yr)	Scenario 2 = 62.93	Scenario 4 = 51.58	Scenario 6 = 66.66	Scenario 8 = 2.99
	Scenario 3 = 63.40	Scenario 5 = 51.99	Scenario 7 = 67.08	Scenario 9 = 2.99
t	-0.75	-0.76	-0.70	-0.206
df	3114.7	3112	3105.2	3118
p-value	0.4528^++^	0.4497^++^	0.483^++^	0.8365^++^

^++^ p-value is not statistically significant at a 95% confidence level. Hence the null hypothesis that there is no difference in the means is accepted (not rejected).

Results in Table 4 indicate that at the prevailing vaccination coverage of 26.5% (with or without the provision of government subsidies), the average gross margin earning at the farm level is 42,728.36 FCFA ($69.43) (annually) more than the no-vaccination scenario (Scenario 1). The prevailing 26.5% vaccination coverage will result in the estimated per capita consumption for mutton and goat meat to increase by 1.13kg/person/year, compared to the baseline levels (no vaccination scenario). When the vaccination coverage is increased to the prescribed threshold for PPR eradication (i.e., 70%) with or without the provision of government subsidies, the average gross margin earning at the farm level will be 44,451.53 FCFA ($72.23) annually, and per capita consumption of mutton and goat meat to 1.23kg/person/year.

Also, the results revealed a statistically significant difference in the gross margin earnings at the farm level when the vaccination coverage is increased from the prevailing 26.5% to the prescribed eradication threshold of 70%. The increase in vaccination coverage will translate to a 4% increase in the gross margin earnings (i.e., 1,723.17 FCFA ($2.8)) annually (with or without the provision of government subsidies), and the per capita consumption will increase by 0.1kg/person/year.

### 3.2 Impact of vaccine wastage

The potential impact of vaccine wastage emanating from multiple vaccinations due to the inability to differentiate between vaccinated and unvaccinated animals was also explored. Results showed that when 10% of vaccinated animals are mistakenly re-vaccinated, the average gross margin earnings at the farm level decrease by 6,997.28 FCFA ($11.37) annually for a vaccination coverage of 26.5%. The decline in gross margin earnings dips further by 7,988.11 FCFA ($12.98) annually when the vaccination coverage is 70%. A 10% vaccine wastage at the farm level will correspond with a decrease in the per capita consumption by 0.3kg/person/year and 0.35kg/person/year when vaccination coverage is at 26.5% and 70%, respectively.

### 3.3 Discussion

This study’s findings provide a socioeconomic justification for the adoption of vaccination. While the estimated gross margin at the individual household level serves as a suitable economic indicator to stimulate small ruminant producers’ interest to engage in vaccination, there are differences in the suitability of indicators for socioeconomic impact assessment. Livestock rearing is sometimes a status symbol [23]. Hence, the motive for keeping small ruminants is not always only profit oriented. This situation is more prevalent in wealthy households. However, poor households often have the target of selling. As such, the use of gross margin as a socioeconomic indicator may be more appropriate for economically poor farm households than for wealthy households.

The economic loss due to PPR is another measure that can be used. However, a low economic loss due to PPR-related mortality has generally been reported at village levels in Nigeria ($3.8 to $14.6) and community levels in Pakistan ($33) [12]. While the economic impact of PPR control is often assessed at aggregated at national levels [1, 5, 12], the disaggregation of the economic impact at the household level will be a more effective way of stimulating the buy-in of farmers to voluntarily participate and even be willing to pay for vaccination. Nevertheless, the relatively low economic loss at individual levels may not incentivise farm households to adopt vaccination.

Furthermore, the findings provide evidence for the benefit of vaccination with or without subsidies from the government. Consequently, the willingness of farm households to vaccinate is crucial for the sustainable eradication of PPR in Senegal. Lessons can be drawn from the study on farmers’ willingness to vaccinate in Mali [24], which reported that farmers’ willingness to pay for vaccination increases when they are aware of the benefits of vaccination. Indeed, public awareness campaign is an integral principle of disease control [1]. Specifically, the media for disseminating information on the benefits and the content of the information are important for an effective public awareness campaign. Awareness campaigns at grassroot levels (like farm, community, and village levels) at vital meeting points of the targeted audience are crucial for the effective dissemination of the benefits of vaccination [2, 24].

Often the withdrawal of subsidies is accompanied by poor uptake of development-oriented interventions. Consequently, donors find themselves in a Samaritan’s Dilemma [25]. A well-organised policy is a key ingredient for effective official development assistance [26]. Therefore, a policy implication from this study’s findings is that for a long-term and sustainable implementation of the Global Control and Eradication Strategy for *peste des petit ruminants*, attention and resources should be dedicated to motivating farmer households to co-finance vaccination against PPR. A suite of interventions may be appropriate for effective vaccination exercise. For instance, given the critical role of feed purchases as a driver of economic viability, the provision of feed supplements and vaccines could be the silver lining intervention combination to incentivise farm households to be willing to co-finance vaccination.

Also, the belief system has a role in farmers’ decision to vaccinate [24]. This study’s findings provide evidence for the financial implication of vaccine wastage emanating from multiple vaccinations of already vaccinated animals due to farmers’ belief that marking animals makes the animal unwholesome. Since animal identification has many other benefits for disease control and might even be used to increase market value, reducing vaccination wastage may be another important argument for setting up better and acceptable animal identification systems.

## 4 Conclusions

This study sought to examine the *ex-ante* impact of different vaccination scenarios on the economic viability of small ruminant production at the individual farm level and the consequential impact on the potential supply of mutton and goat meat for consumption at the national level. This study’s findings provide empirical evidence of the socioeconomic benefits of vaccination. Generally, increasing the vaccination coverage corresponds to increasing gross margin and the potential per capita consumption. However, the cost of feed is crucial for economic viability. Consequently, the provision of feed supplements can be a suitable accompaniment for vaccine delivery to incentivise farm households’ willingness to vaccinate, especially in the dry season.

Considering that there was no statistically significant difference in the gross margin earnings and the potential per capita consumption when vaccination is performed with or without government subsidies, a sustainable strategy for PPR eradication will include the promotion of the benefits of vaccination via sensitization campaigns to stimulate farmers’ uptake of the practice. Also, there is room to explore innovative ways to mark small ruminants that are vaccinated to reduce vaccine wastage. Such innovation will factor in the socio-cultural concerns impeding the marking of animals after vaccination.

An underlying assumption in the model is that logistics for vaccine delivery are effective across country. Future extension of the model can incorporate the economic impact of logistics like cold chains or remoteness for vaccine delivery. Such studies could consider the climatic variability in the country affecting accessibility of remote areas and explore the optimum time for vaccine delivery.

## Supporting information

S1 File **Appendix A**: Comparative analysis for gross margin at farm-level. **Appendix B**: Comparative Analysis for Per Capita Consumption at National Level.
(DOCX)

S2 File (DOCX)
