# Correction: Ex-ante impact of pest des petits ruminant control on micro and macro socioeconomic indicators in Senegal: A system dynamics modelling approach

**DOI:** 10.1371/journal.pone.0317858

**Published:** 2025-01-16

**Authors:** Joshua Aboah, Andrea Apolloni, Raphaël Duboz, Barbara Wieland, Pacem Kotchofa, Edward Okoth, Michel Dione

The word "peste" and "ruminants" are misspelled in the article title. The correct title is: Ex-ante impact of peste des petits ruminants control on micro and macro socioeconomic indicators in Senegal: A system dynamics modelling approach. The correct citation is: Aboah J, Apolloni A, Duboz R, Wieland B, Kotchofa P, Okoth E, et al. (2023) Ex-ante impact of peste des petits ruminants control on micro and macro socioeconomic indicators in Senegal: A system dynamics modelling approach. PLOS ONE 18(7): e0287386. https://doi.org/10.1371/journal.pone.0287386
