# Supplementary material for: Ex-ante impact of pest des petits ruminant control on micro and macro socioeconomic indicators in Senegal: A system dynamics modelling approach
Source: PLoS One. 2023 Jul 5;18(7):e0287386. doi: 10.1371/journal.pone.0287386 (PMC10321633; doi:10.1371/journal.pone.0287386)
Supplement: S1 File — Appendix A: Comparative analysis for gross margin at farm-level. Appendix B: Comparative Analysis for Per Capita Consumption at National Level. (DOCX) [file pone.0287386.s001.docx]

# Supporting information

# Appendix A: Comparative Analysis for Gross Margin at Farm-level

# **S1 TableComparison of the baseline gross margin (Scenario 1) and the other vaccination scenarios**

| Scenario 1 Vs. Scenario (i) (where i=2,3,4,5,6,7,8,9) | Mean (scenario 1)  [$ per week] | Mean (scenario 1)  [$ per year] | Mean (scenario i)  [$ per week] | Mean (scenario i)  [$ per year] | t | df | p-value |
| --- | --- | --- | --- | --- | --- | --- | --- |
| Scenario 1 Vs. Scenario i=2 | -0.23 | -11.95 | 1.21 | 62.93 | -59.66 | 1964.7 | <2.2e-16** |
| Scenario 1 Vs. Scenario i=3 |  |  | 1.22 | 63.40 | -59.66 | 1964.7 | <2.2e-16** |
| Scenario 1 Vs. Scenario i=4 |  |  | 0.992 | 51.58 | -50.87 | 1890.2 | <2.2e-16** |
| Scenario 1 Vs. Scenario i=5 |  |  | 0.999 | 51.99 | -52.41 | 1862.9 | <2.2e-16** |
| Scenario 1 Vs. Scenario i=6 |  |  | 1.28 | 66.66 | -62.20 | 1968.9 | <2.2e-16** |
| Scenario 1 Vs. Scenario i=7 |  |  | 1.29 | 67.08 | -62.98 | 1921 | <2.2e-16** |
| Scenario 1 Vs. Scenario i=8 |  |  | 1.03 | 53.67 | -52.65 | 1875.8 | <2.2e-16** |
| Scenario 1 Vs. Scenario i=9 |  |  | 1.04 | 54.11 | -53.39 | 1827.3 | <2.2e-16** |

# **S2 TableComparison of the gross margin for Scenario 2 and vaccination scenarios 3,4,5,6,7,8 and 9**

| Scenario (2) Vs. Scenario (i) (where i=3,4,5,6,7,8,9) | Mean (scenario 2)  [$ per week] | Mean (scenario 2)  [$ per year] | Mean (scenario i)  [$ per week] | Mean (scenario i)  [$ per year] | t | df | p-value |
| --- | --- | --- | --- | --- | --- | --- | --- |
| Scenario 2 Vs. Scenario i=3 | 1.21 | 62.93 | 1.22 | 63.40 | -0.75 | 3114.7 | 0.4528^++^ |
| Scenario 2 Vs. Scenario i=4 |  |  | 0.992 | 51.58 | 19.16 | 3061.1 | <2.2e-16** |
| Scenario 2 Vs. Scenario i=5 |  |  | 0.999 | 51.99 | 18.817 | 3020.9 | <2.2e-16** |
| Scenario 2 Vs. Scenario i=6 |  |  | 1.28 | 66.66 | -5.99 | 3115.7 | 2.30e-09** |
| Scenario 2 Vs. Scenario i=7 |  |  | 1.29 | 67.08 | -6.87 | 3092.2 | 7.85e-12** |
| Scenario 2 Vs. Scenario i=8 |  |  | 1.03 | 53.67 | 15.79 | 3041.6 | <2.2e-16** |
| Scenario 2 Vs. Scenario i=9 |  |  | 1.04 | 54.11 | 15.57 | 2948.5 | <2.2e-16** |

** p-value is statistically significant at 95% confidence level. Hence the null hypothesis that there is no difference in the means is rejected for the alternative that there is difference in the means

++ p-value is not statistically significant at 95% confidence level. Hence the null hypothesis that there is no difference in the means is accepted (not rejected).

# **S3 Table Comparison of the gross margin for Scenario 3 and vaccination scenarios 4,5,6,7,8 and 9**

| Scenario (3) Vs. Scenario (i) (where i=4,5,6,7,8,9) | Mean (scenario 3)  [$ per week] | Mean (scenario 3)  [$ per year] | Mean (scenario i)  [$ per week] | Mean (scenario i)  [$ per year] | t | df | p-value |
| --- | --- | --- | --- | --- | --- | --- | --- |
| Scenario 3 Vs. Scenario i=4 | 1.22 | 63.40 | 0.992 | 51.58 | 20.32 | 3084.6 | <2.2e-16** |
| Scenario 3 Vs. Scenario i=5 |  |  | 0.999 | 51.99 | 19.99 | 3051.7 | <2.2e-16** |
| Scenario 3 Vs. Scenario i=6 |  |  | 1.28 | 66.66 | -5.33 | 3117.9 | 1.05e-07** |
| Scenario 3 Vs. Scenario i=7 |  |  | 1.29 | 67.08 | -6.204 | 3107.3 | 6.22e-10** |
| Scenario 3 Vs. Scenario i=8 |  |  | 1.03 | 53.67 | 16.90 | 3069 | <2.2e-16** |
| Scenario 3 Vs. Scenario i=9 |  |  | 1.04 | 54.11 | 16.72 | 2988 | <2.2e-16** |

# **S4 Table Comparison of the gross margin for Scenario 4 and vaccination scenarios 5,6,7,8 and 9**

| Scenario (4) Vs. Scenario (i)(where i=5,6,7,8,9) | Mean (Scenario 4)  [$ per week] | Mean (Scenario 4)  [$ per year] | Mean (Scenario i)  [$ per week] | Mean (Scenario i)  [$ per year] | t | df | p-value |
| --- | --- | --- | --- | --- | --- | --- | --- |
| Scenario 4 Vs. Scenario i=5 | 0.992 | 51.58 | 0.999 | 51.99 | -0.76 | 3112 | 0.4497^++^ |
| Scenario 4 Vs. Scenario i=6 |  |  | 1.28 | 66.66 | -25.86 | 3081.2 | <2.2e-16** |
| Scenario 4 Vs. Scenario i=7 |  |  | 1.29 | 67.08 | -27.51 | 3111.5 | <2.2e-16** |
| Scenario 4 Vs. Scenario i=8 |  |  | 1.03 | 53.67 | -3.83 | 3116.4 | 0.00013** |
| Scenario 4 Vs. Scenario i=9 |  |  | 1.04 | 54.11 | -4.812 | 3082.8 | 1.54e-06** |

# **S5 Table Comparison of the gross margin for Scenario 5 and vaccination scenarios 6,7,8 and 9**

| Scenario (5) Vs. Scenario (i)(where i=6,7,8,9) | Mean (Scenario 5)  [$ per week] | Mean (Scenario 5)  [$ per year] | Mean (Scenario i)  [$ per week] | Mean (Scenario i)  [$ per year] | t | df | p-value |
| --- | --- | --- | --- | --- | --- | --- | --- |
| Scenario 5 Vs. Scenario i=6 | 0.999 | 51.99 | 1.28 | 66.66 | -25.64 | 3047.1 | <2.2e-16** |
| Scenario 5 Vs. Scenario i=7 |  |  | 1.29 | 67.08 | -27.34 | 3093.3 | <2.2e-16** |
| Scenario 5 Vs. Scenario i=8 |  |  | 1.03 | 53.67 | -3.15 | 3116.6 | 0.0016** |
| Scenario 5 Vs. Scenario i=9 |  |  | 1.04 | 54.11 | -4.13 | 3105.6 | 3.65e-05** |

# **S6 Table Comparison of the gross margin for Scenario 6 and vaccination scenarios 7,8 and 9**

| Scenario (6) Vs. Scenario (i)(where i=7,8,9) | Mean (Scenario 6)  [$ per week] | Mean (Scenario 6)  [$ per year] | Mean (Scenario i)  [$ per week] | Mean (Scenario i)  [$ per year] | t | df | p-value |
| --- | --- | --- | --- | --- | --- | --- | --- |
| Scenario 6 Vs. Scenario i=7 | 1.28 | 66.66 | 1.29 | 67.08 | -0.70 | 3105.2 | 0.483^++^ |
| Scenario 6 Vs. Scenario i=8 |  |  | 1.03 | 53.67 | 22.50 | 3064.9 | <2.2e-16** |
| Scenario 6 Vs. Scenario i=9 |  |  | 1.04 | 54.11 | 22.53 | 2981.8 | <2.2e-16** |

# **S7 Table Comparison of the gross margin for Scenario 7 and vaccination scenarios 8 and 9**

| Scenario (7) Vs. Scenario (i) (where i=8,9) | Mean (Scenario 7)  [$ per week] | Mean (Scenario 7)  [$ per year] | Mean (Scenario i)  [$ per week] | Mean (Scenario i)  [$ per year] | t | df | p-value |
| --- | --- | --- | --- | --- | --- | --- | --- |
| Scenario 7 Vs. Scenario i=8 | 1.29 | 67.08 | 1.03 | 53.67 | 24.06 | 3103.6 | <2.2e-16 ** |
| Scenario 7 Vs. Scenario i=9 |  |  | 1.04 | 54.11 | 24.177 | 3047.9 | <2.2e-16 ** |

# **S8 Table Comparison of the gross margin for Scenario 8 and vaccination scenario 9**

| Scenario (8) Vs. Scenario (9) | Mean (Scenario 8)  [$ per week] | Mean (Scenario 8)  [$ per year] | Mean (Scenario 9)  [$ per week] | Mean (Scenario 9)  [$ per year] | t | df | p-value |
| --- | --- | --- | --- | --- | --- | --- | --- |
| Scenario 8 Vs. Scenario 9 | 1.03 | 53.67 | 1.04 | 54.11 | -0.84 | 3095.9 | 0.402^++^ |

# Appendix B: Comparative Analysis for Per Capita Consumption at National Level

# **S9 TableComparison of the baseline per capita consumption and the other vaccination scenarios**

| Scenario (1) Vs. Scenario (i) where i=2,3,4,5,6,7,8,9 | Mean (scenario 1)  [kg/person/week] | Mean (scenario 1)  [kg/person/year] | Mean (scenario i)  [kg/person/week] | Mean (scenario i) [kg/person/year] | t | df | p-value |
| --- | --- | --- | --- | --- | --- | --- | --- |
| Scenario 1 Vs. Scenario i=2 | 0.04 | 1.94 | 0.062 | 3.22 | -156.6 | 2333.8 | <2.2e-16** |
| Scenario 1 Vs. Scenario i=3 |  |  | 0.062 | 3.22 | -157.12 | 2336.2 | <2.23-16** |
| Scenario 1 Vs. Scenario i=4 |  |  | 0.056 | 2.93 | -138.22 | 2600.3 | <2.23-16** |
| Scenario 1 Vs. Scenario i=5 |  |  | 0.056 | 2.93 | -138.66 | 2603.7 | <2.23-16** |
| Scenario 1 Vs. Scenario i=6 |  |  | 0.064 | 3.35 | -173.69 | 2353.2 | <2.23-16** |
| Scenario 1 Vs. Scenario i=7 |  |  | 0.064 | 3.35 | -174.01 | 2354.7 | <2.23-16** |
| Scenario 1 Vs. Scenario i=8 |  |  | 0.058 | 2.99 | -150.11 | 2628.8 | <2.2e-16** |
| Scenario 1 Vs. Scenario i=9 |  |  | 0.058 | 2.99 | -150.6 | 2632.2 | <2.2e-16** |

# **S10 Table Comparison of the per capita consumption for Scenario 2 and Scenarios 3,4,5,6,7,8 and 9**

| Scenario (2) Vs. Scenario (i) where i=3,4,5,6,7,8,9 | Mean (scenario 2)  [kg/person/week] | Mean (scenario 2)  [kg/person/year] | Mean (scenario i)  [kg/person/week] | Mean (scenario i) [kg/person/year] | t | df | p-value |
| --- | --- | --- | --- | --- | --- | --- | --- |
| Scenario 2 Vs. Scenario i=3 | 0.062 | 3.22 | 0.062 | 3.22 | -0.23 | 3118 | 0.8158^++^ |
| Scenario 2 Vs. Scenario i=4 |  |  | 0.056 | 2.93 | 31.27 | 3020.1 | <2.2e-16** |
| Scenario 2 Vs. Scenario i=5 |  |  | 0.056 | 2.93 | 31.13 | 3017.8 | <2.2e-16** |
| Scenario 2 Vs. Scenario i=6 |  |  | 0.064 | 3.35 | -12.14 | 3117.4 | <2.2e-16** |
| Scenario 2 Vs. Scenario i=7 |  |  | 0.064 | 3.35 | -12.27 | 3117.3 | <2.2e-16** |
| Scenario 2 Vs. Scenario i=8 |  |  | 0.058 | 2.99 | 23.98 | 3000.7 | <2.2e-16** |
| Scenario 2 Vs. Scenario i=9 |  |  | 0.058 | 2.99 | 23.81 | 2998.2 | <2.2e-16** |

** p-value is statistically significant at 95% confidence level. Hence the null hypothesis that there is no difference in the means is rejected for the alternative that there is difference in the means

++ p-value is not statistically significant at 95% confidence level. Hence the null hypothesis that there is no difference in the means is accepted (not rejected).

# **S11 Table Comparison of the per capita consumption for Scenario 3 and Scenarios 4,5,6,7,8 and 9**

| Scenario (3) Vs. Scenario (i) where i=4,5,6,7,8,9 | Mean (Scenario 3)  [kg/person/week] | Mean (Scenario 3)  [kg/person/year] | Mean (Scenario i)  [kg/person/week] | Mean (scenario i) [kg/person/year] | t | df | p-value |
| --- | --- | --- | --- | --- | --- | --- | --- |
| Scenario 3 Vs. Scenario i=4 | 0.062 | 3.22 | 0.056 | 2.93 | 31.56 | 3022 | <2.2e-16** |
| Scenario 3 Vs. Scenario i=5 |  |  | 0.056 | 2.93 | 31.42 | 3019.7 | <2.2e-16** |
| Scenario 3 Vs. Scenario i=6 |  |  | 0.064 | 3.35 | -11.91 | 3117.5 | <2.2e-16** |
| Scenario 3 Vs. Scenario i=7 |  |  | 0.064 | 3.35 | -12.05 | 3117.4 | <2.2e-16** |
| Scenario 3 Vs. Scenario i=8 |  |  | 0.058 | 2.99 | 24.26 | 3002.7 | <2.2e-16** |
| Scenario 3 Vs. Scenario i=9 |  |  | 0.058 | 2.99 | 24.09 | 3000.3 | <2.2e-16** |

# **S12 Table Comparison of the per capita consumption for Scenario 4 and Scenarios 5,6,7,8 and 9**

| Scenario (4) Vs. Scenario (i) where i=5,6,7,8,9 | Mean (scenario 4)  [kg/person/week] | Mean (scenario 4)  [kg/person/year] | Mean (scenario i)  [kg/person/week] | Mean (scenario i) [kg/person/year] | t | df | p-value |
| --- | --- | --- | --- | --- | --- | --- | --- |
| Scenario 4 Vs. Scenario i=5 | 0.056 | 2.93 | 0.056 | 2.93 | -0.184 | 3118 | 0.854^++^ |
| Scenario 4 Vs. Scenario i=6 |  |  | 0.064 | 3.35 | -44.74 | 3034.2 | <2.2e-16** |
| Scenario 4 Vs. Scenario i=7 |  |  | 0.064 | 3.35 | -44.91 | 3035.3 | <2.2e-16** |
| Scenario 4 Vs. Scenario i=8 |  |  | 0.058 | 2.99 | -8.33 | 3117 | <2.2e-16** |
| Scenario 4 Vs. Scenario i=9 |  |  | 0.058 | 2.99 | -8.54 | 3116.7 | <2.2e-16** |

# **S13 Table Comparison of the per capita consumption for Scenario 5 and Scenarios 6,7,8 and 9**

| Scenario (5) Vs. Scenario (i)where i=6,7,8,9 | Mean (scenario 5)  [kg/person/week] | Mean (scenario 5)  [kg/person/year] | Mean (scenario i)  [kg/person/week] | Mean (scenario i) [kg/person/year] | t | df | p-value |
| --- | --- | --- | --- | --- | --- | --- | --- |
| Scenario 5 Vs. Scenario i=6 | 0.056 | 2.93 | 0.064 | 3.35 | -44.61 | 3032.1 | <2.2e-16** |
| Scenario 5 Vs. Scenario i=7 |  |  | 0.064 | 3.35 | -44.78 | 3033.2 | <2.2e-16** |
| Scenario 5 Vs. Scenario i=8 |  |  | 0.058 | 2.99 | -8.15 | 3117.2 | 5.09e-16** |
| Scenario 5 Vs. Scenario i=9 |  |  | 0.058 | 2.99 | -8.37 | 3117 | <2.2e-16** |

# **S14 Table Comparison of the per capita consumption for Scenario 6 and Scenarios 7,8 and 9**

| Scenario (6) Vs. Scenario (i) where i=7,8,9 | Mean (scenario 6)  [kg/person/week] | Mean (scenario 6)  [kg/person/year] | Mean (scenario i)  [kg/person/week] | Mean (scenario i) [kg/person/year] | t | df | p-value |
| --- | --- | --- | --- | --- | --- | --- | --- |
| Scenario 6 Vs. Scenario i=7 | 0.064 | 3.35 | 0.064 | 3.35 | -0.13 | 3118 | 0.8957^++^ |
| Scenario 6 Vs. Scenario i=8 |  |  | 0.058 | 2.99 | 37.48 | 3016 | <2.2e-16** |
| Scenario 6 Vs. Scenario i=9 |  |  | 0.058 | 2.99 | 37.33 | 3013.7 | <2.2e-16** |

# **S15 Table Comparison of the per capita consumption for Scenario 7 and Scenarios 8 and 9**

| Scenario (7) Vs. Scenario (i) where i=8,9 | Mean (scenario 7)  [kg/person/week] | Mean (scenario 7)  [kg/person/year] | Mean (scenario i)  [kg/person/week] | Mean (scenario i) [kg/person/year] | t | df | p-value |
| --- | --- | --- | --- | --- | --- | --- | --- |
| Scenario 7 Vs. Scenario i=8 | 0.064 | 3.35 | 0.058 | 2.99 | 37.65 | 3017.1 | <2.2e-16 ** |
| Scenario 7 Vs. Scenario i=9 |  |  | 0.058 | 2.99 | 37.50 | 3014.8 | <2.2e-16 ** |

# **S16 Table Comparison of the per capita consumption for Scenario 8 and Scenario and 9**

| Scenario (8) Vs. Scenario (9) | Mean (scenario 8)  [kg/person/week] | Mean (scenario 8)  [kg/person/year] | Mean (scenario 9)  [kg/person/week] | Mean (scenario 9) [kg/person/year] | t | df | p-value |
| --- | --- | --- | --- | --- | --- | --- | --- |
| Scenario 8 Vs. Scenario 9 | 0.058 | 2.99 | 0.058 | 2.99 | -0.206 | 3118 | 0.8365^++^ |
