# Supplementary material for: Ex-ante impact of pest des petits ruminant control on micro and macro socioeconomic indicators in Senegal: A system dynamics modelling approach
Source: PLoS One. 2023 Jul 5;18(7):e0287386. doi: 10.1371/journal.pone.0287386 (PMC10321633; doi:10.1371/journal.pone.0287386)
Supplement: S2 File — (DOCX) [file pone.0287386.s002.docx]

Supplementary File

| Total | Count | Including Array Elements |
| --- | --- | --- |
| Variables | 602 | 2039 |
| Modules | 5 |  |
| Sectors | 9 |  |
| Stocks | 98 | 377 |
| Flows | 148 | 565 |
| Converters | 356 | 1097 |
| Constants | 97 | 140 |
| Equations | 407 | 1522 |
| Graphicals | 14 | 14 |
| Macro Variables | 196 |  |

|  | Equation | Properties | Units | Documentation | Annotation |
| --- | --- | --- | --- | --- | --- |
| Top-Level Model: | | | | | |
| Disease_Control_Module: | | | | | |
| "Adult_billy_>12_months_vaccination_rate"[HHD_Gender, SR_activity_responsibility] | "Vaccination_coverage_Adult_billy_>_12_months"*("Production-_Epidemiological_Module"."Adult_billy_>_12_months_susceptible"/"Time_to_deploy_vaccine_for_Adult_goat_>_12_months") |  | Goat/Weeks |  |  |
| "Adult_Ewe_>_12_months_vaccination_rate"[HHD_Gender, SR_activity_responsibility] | "Vaccination_coverage_Adult_Ewe_>12_months"*("Production-_Epidemiological_Module"."Adult_Ewe_>_12_months_susceptible"/"Time_to_deploy_vaccine_Adult_sheep_>12_months") |  | Sheep per week |  |  |
| "Adult_nanny_>_12_months_vaccination_rate"[HHD_Gender, SR_activity_responsibility] | "Vaccination_coverage_Adult_nanny_>12_months"*("Production-_Epidemiological_Module"."Adult_nanny_>_12_months_susceptible"/"Time_to_deploy_vaccine_for_Adult_goat_>_12_months") |  | Goat/Weeks |  |  |
| "Adult_Ram_>12_months_vaccination_rate"[HHD_Gender, SR_activity_responsibility] | "Vaccination_coverage_Adult_Ram_>12_months"*("Production-_Epidemiological_Module"."Adult_Ram_>12_months_susceptible"/"Time_to_deploy_vaccine_Adult_sheep_>12_months") |  | Sheep per week |  |  |
| Grower_billy_6_to_12_months_vaccination_rate[HHD_Gender, SR_activity_responsibility] | Vaccination_coverage_grower_billy_6_to_12_months*("Production-_Epidemiological_Module".Grower_billy_6_to_12_months_susceptible/Time_to_deploy_vaccine_for_Grower_goat_6_to_12_months) |  | Goat/Weeks |  |  |
| Grower_Ewes_6_to_12_months_vaccination_rate[HHD_Gender, SR_activity_responsibility] | Vaccination_coverage_Grower_Ewes_6_to_12_months*("Production-_Epidemiological_Module".Grower_Ewe_6_to_12_months_susceptible/Time_to_deploy_vaccine_Grower_sheep_6_to_12_months) |  | Sheep per week |  |  |
| Grower_nanny_6_to_12_months_vaccination_rate[HHD_Gender, SR_activity_responsibility] | Vaccination_coverage_grower_nanny_6_to_12_month*("Production-_Epidemiological_Module".Grower_nanny_6_to_12_months_susceptible/Time_to_deploy_vaccine_for_Grower_goat_6_to_12_months) |  | Goat/Weeks |  |  |
| Grower_Rams_6_to_12_months_vaccination_rate[HHD_Gender, SR_activity_responsibility] | ("Production-_Epidemiological_Module".Grower_Rams_6_to_12_months_Susceptible/Time_to_deploy_vaccine_Grower_sheep_6_to_12_months)*Vaccination_coverage_Grower_Rams_6_to_12_months |  | Sheep per week |  |  |
| "Sero-conversion_rate"[HHD_Gender, SR_activity_responsibility] | IF("Production-_Epidemiological_Module".Expected_production_investment=1)THEN 1 ELSE 0 |  | Dimensionless |  |  |
| "Time_to_deploy_vaccine_Adult_sheep_>12_months" | 1 |  | Weeks |  |  |
| "Time_to_deploy_vaccine_for_Adult_goat_>_12_months" | 1 |  | Weeks |  |  |
| Time_to_deploy_vaccine_for_Grower_goat_6_to_12_months | 1 |  | Weeks |  |  |
| Time_to_deploy_vaccine_for_Young_goat_3_to_6_months | 1 |  | Weeks |  |  |
| Time_to_deploy_vaccine_Grower_sheep_6_to_12_months | 1 |  | Weeks |  |  |
| Time_to_deploy_vaccine_Young_sheep_3_to_6_months | 1 |  | Weeks |  |  |
| Vaccination_coverage_'young_nanny_3_to_6_months[HHD_Gender, SR_activity_responsibility] | IF((Policy_Module.Government_subsidy>0) OR(Economic_Module.Profit >0)) THEN 1 ELSE 0 |  | Dimensionless |  |  |
| "Vaccination_coverage_Adult_billy_>_12_months"[HHD_Gender, SR_activity_responsibility] | IF((Policy_Module.Government_subsidy>0) OR(Economic_Module.Profit >0)) THEN 1 ELSE 0 |  | Dimensionless |  |  |
| "Vaccination_coverage_Adult_Ewe_>12_months"[HHD_Gender, SR_activity_responsibility] | IF((Policy_Module.Government_subsidy>0) OR(Economic_Module.Profit >0)) THEN 1 ELSE 0 |  | Dimensionless |  |  |
| "Vaccination_coverage_Adult_nanny_>12_months"[HHD_Gender, SR_activity_responsibility] | IF((Policy_Module.Government_subsidy>0) OR(Economic_Module.Profit >0)) THEN 1 ELSE 0 |  | Dimensionless |  |  |
| "Vaccination_coverage_Adult_Ram_>12_months"[HHD_Gender, SR_activity_responsibility] | IF((Policy_Module.Government_subsidy>0) OR(Economic_Module.Profit >0)) THEN 1 ELSE 0 |  | Dimensionless |  |  |
| Vaccination_coverage_grower_billy_6_to_12_months[HHD_Gender, SR_activity_responsibility] | IF((Policy_Module.Government_subsidy>0) OR(Economic_Module.Profit >0)) THEN 1 ELSE 0 |  | Dimensionless |  |  |
| Vaccination_coverage_Grower_Ewes_6_to_12_months[HHD_Gender, SR_activity_responsibility] | IF((Policy_Module.Government_subsidy>0) OR(Economic_Module.Profit >0)) THEN 1 ELSE 0 |  | Dimensionless |  |  |
| Vaccination_coverage_grower_nanny_6_to_12_month[HHD_Gender, SR_activity_responsibility] | IF((Policy_Module.Government_subsidy>0) OR(Economic_Module.Profit >0)) THEN 1 ELSE 0 |  | Dimensionless |  |  |
| Vaccination_coverage_Grower_Rams_6_to_12_months[HHD_Gender, SR_activity_responsibility] | IF((Policy_Module.Government_subsidy>0) OR(Economic_Module.Profit >0)) THEN 1 ELSE 0 |  | Dimensionless |  |  |
| Vaccination_coverage_young_billy_3_to_6_months[HHD_Gender, SR_activity_responsibility] | IF((Policy_Module.Government_subsidy>0) OR(Economic_Module.Profit >0)) THEN 1 ELSE 0 |  | Dimensionless |  |  |
| Vaccination_coverage_Young_Ewe_3_to_6_months[HHD_Gender, SR_activity_responsibility] | IF((Policy_Module.Government_subsidy>0) OR(Economic_Module.Profit >0)) THEN 1 ELSE 0 |  | Dimensionless |  |  |
| Vaccination_coverage_Young_Ram_3_to_6_months[HHD_Gender, SR_activity_responsibility] | IF((Policy_Module.Government_subsidy>0) OR(Economic_Module.Profit >0)) THEN 1 ELSE 0 |  | Dimensionless |  |  |
| Young_billy_3_to_6_months_vaccination_rate[HHD_Gender, SR_activity_responsibility] | Vaccination_coverage_young_billy_3_to_6_months*("Production-_Epidemiological_Module".Young_billy_3_to_6_months_susceptible/Time_to_deploy_vaccine_for_Young_goat_3_to_6_months) |  | Goat/Weeks |  |  |
| Young_Ewe_3_to_6_months_Vaccination_rate[HHD_Gender, SR_activity_responsibility] | Vaccination_coverage_Young_Ewe_3_to_6_months*("Production-_Epidemiological_Module".Young_Ewe_3_to_6_months_susceptible/Time_to_deploy_vaccine_Young_sheep_3_to_6_months) |  | Sheep per week |  |  |
| Young_nanny_3_to_6_months_vaccination_rate[HHD_Gender, SR_activity_responsibility] | Vaccination_coverage_'young_nanny_3_to_6_months*("Production-_Epidemiological_Module".Young_nanny_3_to_6_months_susceptible/Time_to_deploy_vaccine_for_Young_goat_3_to_6_months) |  | Goat/Weeks |  |  |
| Young_Ram_3_to_6_months_Vaccination_rate[HHD_Gender, SR_activity_responsibility] | ("Production-_Epidemiological_Module".Young_Ram_3_to_6_months_susceptible/Time_to_deploy_vaccine_Young_sheep_3_to_6_months)*Vaccination_coverage_Young_Ram_3_to_6_months |  | Sheep per week |  |  |
| Economic_Module: | | | | | |
| Annual_Cumulative_profit[HHD_Gender, SR_activity_responsibility](t) | Annual_Cumulative_profit[HHD_Gender, SR_activity_responsibility](t - dt) + (Profit_over_time[HHD_Gender, SR_activity_responsibility] - Profit[HHD_Gender, SR_activity_responsibility]) * dt | INIT Annual_Cumulative_profit[HHD_Gender, SR_activity_responsibility] = 0 | CFA |  |  |
| Profit[HHD_Gender, SR_activity_responsibility] | PULSE(Annual_Cumulative_profit, 52, Interval_of_profit) |  | CFA/Weeks |  | UNIFLOW |
| Profit_over_time[HHD_Gender, SR_activity_responsibility] | PULSE(Gross_Margin, 52, Interval_of_profit) |  | CFA/Weeks |  |  |
| Actual_vaccination_cost_goat[HHD_Gender, SR_activity_responsibility] | TRIANGULAR(150.6, 213.35, 338.85) {Tago et al., 2017 Vaccin cost - Min $0.24 Mean= $0.34, Max= $0.54} |  | CFA per goat |  |  |
| Actual_vaccination_cost_sheep[HHD_Gender, SR_activity_responsibility] | TRIANGULAR(150.6, 213.35, 338.85) {Tago et al., 2017 Vaccin cost - Min $0.24 Mean= $0.34, Max= $0.54} |  | CFA per sheep |  |  |
| Adult_goat_feeding_cost[HHD_Gender, SR_activity_responsibility] | Feed_intake_adult_goat*Average_weekly_feeding_cost_sheep[Adult] |  | CFA/Weeks |  |  |
| Adult_sheep_feeding_cost[HHD_Gender, SR_activity_responsibility] | Feed_intake_adult_sheep*Average_weekly_feeding_cost_sheep[Adult] |  | CFA/Weeks |  |  |
| Average_price_fully_grown_Ewe[HHD_Gender, SR_activity_responsibility] | 2500 {HH survey- selling price divided by 40 kg} |  | CFA per kg |  |  |
| Average_price_mature_billy[HHD_Gender, SR_activity_responsibility] | 455{HH survey -selling price divided by 40kg} |  | CFA per kg |  |  |
| Average_price_mature_nanny[HHD_Gender, SR_activity_responsibility] | 1125 {HH survey -selling price divided by 40 kg} |  | CFA per kg |  |  |
| Average_price_mature_Ram[HHD_Gender, SR_activity_responsibility] | 2500{HH survey- selling price divided by 40 kg} |  | CFA per kg |  |  |
| Average_weekly_feed_intake_adult_goat[Animal_age_group, HHD_Gender, SR_activity_responsibility] | IF("Production-_Epidemiological_Module".Feeding_ration[HHD_Gender,SR_activity_responsibility]=2) THEN 2 ELSE 1.5 |  | kg per Goat |  |  |
| Average_weekly_feed_intake_adult_sheep[Animal_age_group, HHD_Gender, SR_activity_responsibility] | IF("Production-_Epidemiological_Module".Feeding_ration[HHD_Gender,SR_activity_responsibility]=2) THEN 2 ELSE 1.5 |  | kg per sheep |  |  |
| Average_weekly_feed_intake_grower_goat[Animal_age_group, HHD_Gender, SR_activity_responsibility] | IF("Production-_Epidemiological_Module".Feeding_ration[HHD_Gender,SR_activity_responsibility]=2) THEN 2 ELSE 1 |  | kg per Goat |  |  |
| Average_weekly_feed_intake_grower_sheep[HHD_Gender, SR_activity_responsibility, Animal_age_group] | IF("Production-_Epidemiological_Module".Feeding_ration[HHD_Gender,SR_activity_responsibility]=2) THEN 1.5 ELSE 1 |  | kg per sheep |  |  |
| Average_weekly_feed_intake_level_sheep[Animal_age_group, HHD_Gender, SR_activity_responsibility] | IF("Production-_Epidemiological_Module".Feeding_ration[HHD_Gender,SR_activity_responsibility]=2) THEN 1 ELSE 0.5 |  | kg per sheep |  |  |
| Average_weekly_feed_intake_young_goat[Animal_age_group, HHD_Gender, SR_activity_responsibility] | IF("Production-_Epidemiological_Module".Feeding_ration[HHD_Gender,SR_activity_responsibility]=2) THEN 1 ELSE 0.5 |  | kg per goat |  |  |
| Average_weekly_feeding_cost_goat[Young] | 2400 |  | CFA per kg per week |  |  |
| Average_weekly_feeding_cost_goat[Grower] | 2400 |  |  |  |  |
| Average_weekly_feeding_cost_goat[Adult] | 2400 |  |  |  |  |
| Average_weekly_feeding_cost_sheep[Young] | 2400 {CFA per kg} |  | CFA per kg per week |  |  |
| Average_weekly_feeding_cost_sheep[Grower] | 2400 {CFA per kg} |  |  |  |  |
| Average_weekly_feeding_cost_sheep[Adult] | 2400 {CFA per kg} |  |  |  |  |
| Average_weight_for_mature_sheep[Young, Male_HHD, HHD] | IF("Production-_Epidemiological_Module".Animal_husbandry_effect_on_productivity[Male_HHD,HHD]=1)THEN 40 ELSE 30 {https://www.fao.org/3/u7600t/u7600T0e.htm} |  | kg per sheep |  |  |
| Average_weight_for_mature_sheep[Young, Male_HHD, Spouse] | IF("Production-_Epidemiological_Module".Animal_husbandry_effect_on_productivity[Male_HHD,Spouse]=1)THEN 40 ELSE 30 {https://www.fao.org/3/u7600t/u7600T0e.htm} |  |  |  |  |
| Average_weight_for_mature_sheep[Young, Female_HHD, HHD] | IF("Production-_Epidemiological_Module".Animal_husbandry_effect_on_productivity[Female_HHD,HHD]=1)THEN 40 ELSE 30 {https://www.fao.org/3/u7600t/u7600T0e.htm} |  |  |  |  |
| Average_weight_for_mature_sheep[Young, Female_HHD, Spouse] | IF("Production-_Epidemiological_Module".Animal_husbandry_effect_on_productivity[Female_HHD,Spouse]=1)THEN 40 ELSE 30 {https://www.fao.org/3/u7600t/u7600T0e.htm} |  |  |  |  |
| Average_weight_for_mature_sheep[Grower, Male_HHD, HHD] | IF("Production-_Epidemiological_Module".Animal_husbandry_effect_on_productivity[Male_HHD,HHD]>1)THEN 10 ELSE 5 |  |  |  |  |
| Average_weight_for_mature_sheep[Grower, Male_HHD, Spouse] | IF("Production-_Epidemiological_Module".Animal_husbandry_effect_on_productivity[Male_HHD,Spouse]>1)THEN 10 ELSE 5 |  |  |  |  |
| Average_weight_for_mature_sheep[Grower, Female_HHD, HHD] | IF("Production-_Epidemiological_Module".Animal_husbandry_effect_on_productivity[Female_HHD,HHD]>1)THEN 10 ELSE 5 |  |  |  |  |
| Average_weight_for_mature_sheep[Grower, Female_HHD, Spouse] | IF("Production-_Epidemiological_Module".Animal_husbandry_effect_on_productivity[Female_HHD,Spouse]>1)THEN 10 ELSE 5 |  |  |  |  |
| Average_weight_for_mature_sheep[Adult, Male_HHD, HHD] | IF("Production-_Epidemiological_Module".Animal_husbandry_effect_on_productivity[Male_HHD,HHD]>1)THEN 10 ELSE 5 |  |  |  |  |
| Average_weight_for_mature_sheep[Adult, Male_HHD, Spouse] | IF("Production-_Epidemiological_Module".Animal_husbandry_effect_on_productivity[Male_HHD,Spouse]>1)THEN 10 ELSE 5 |  |  |  |  |
| Average_weight_for_mature_sheep[Adult, Female_HHD, HHD] | IF("Production-_Epidemiological_Module".Animal_husbandry_effect_on_productivity[Female_HHD,HHD]>1)THEN 10 ELSE 5 |  |  |  |  |
| Average_weight_for_mature_sheep[Adult, Female_HHD, Spouse] | IF("Production-_Epidemiological_Module".Animal_husbandry_effect_on_productivity[Female_HHD,Spouse]>1)THEN 10 ELSE 5 |  |  |  |  |
| Average_weight_gained_goat[Young, Male_HHD, HHD] | IF("Production-_Epidemiological_Module".Animal_husbandry_effect_on_productivity[Male_HHD,HHD]=1) THEN 25 ELSE 20 {http://agtr.ilri.cgiar.org/djallonke} |  | kg per Goat |  |  |
| Average_weight_gained_goat[Young, Male_HHD, Spouse] | IF("Production-_Epidemiological_Module".Animal_husbandry_effect_on_productivity[Male_HHD,Spouse]=1)THEN 25 ELSE 20 {http://agtr.ilri.cgiar.org/djallonke} |  |  |  |  |
| Average_weight_gained_goat[Young, Female_HHD, HHD] | IF("Production-_Epidemiological_Module".Animal_husbandry_effect_on_productivity[Female_HHD,HHD]=1)THEN 25 ELSE 20 |  |  |  |  |
| Average_weight_gained_goat[Young, Female_HHD, Spouse] | IF("Production-_Epidemiological_Module".Animal_husbandry_effect_on_productivity[Female_HHD,Spouse]=1) THEN 25 ELSE 20 {http://agtr.ilri.cgiar.org/djallonke} |  |  |  |  |
| Average_weight_gained_goat[Grower, Male_HHD, HHD] | IF("Production-_Epidemiological_Module".Animal_husbandry_effect_on_productivity[Male_HHD,HHD]=1)THEN 30 ELSE 25 |  |  |  |  |
| Average_weight_gained_goat[Grower, Male_HHD, Spouse] | IF("Production-_Epidemiological_Module".Animal_husbandry_effect_on_productivity[Male_HHD,Spouse]=1)THEN 30 ELSE 25 |  |  |  |  |
| Average_weight_gained_goat[Grower, Female_HHD, HHD] | IF("Production-_Epidemiological_Module".Animal_husbandry_effect_on_productivity[Female_HHD,HHD]=1) THEN 30 ELSE 25 |  |  |  |  |
| Average_weight_gained_goat[Grower, Female_HHD, Spouse] | IF("Production-_Epidemiological_Module".Animal_husbandry_effect_on_productivity[Female_HHD,Spouse]=1)THEN 30 ELSE 25 |  |  |  |  |
| Average_weight_gained_goat[Adult, Male_HHD, HHD] | IF("Production-_Epidemiological_Module".Animal_husbandry_effect_on_productivity[Male_HHD,HHD]=1)THEN 40 ELSE 30 |  |  |  |  |
| Average_weight_gained_goat[Adult, Male_HHD, Spouse] | IF("Production-_Epidemiological_Module".Animal_husbandry_effect_on_productivity[Male_HHD,Spouse]=1)THEN 40 ELSE 30 |  |  |  |  |
| Average_weight_gained_goat[Adult, Female_HHD, HHD] | IF("Production-_Epidemiological_Module".Animal_husbandry_effect_on_productivity[Female_HHD,HHD]=1)THEN 40 ELSE 30 |  |  |  |  |
| Average_weight_gained_goat[Adult, Female_HHD, Spouse] | IF("Production-_Epidemiological_Module".Animal_husbandry_effect_on_productivity[Female_HHD,Spouse]=1)THEN 40 ELSE 30 |  |  |  |  |
| Average_weight_loss_of_mature_goats_due_to_mortality[HHD_Gender, SR_activity_responsibility] | 30 |  | kg per Goat |  |  |
| Average_weight_loss_of_mature_sheep_due_to_mortality[HHD_Gender, SR_activity_responsibility] | 30 {https://www.fao.org/3/u7600t/u7600T0e.htm} |  | kg per sheep |  |  |
| Avoidable_vaccination_cost_goat[HHD_Gender, SR_activity_responsibility] | Unit_vaccination_cost_goat*("Production-_Epidemiological_Module".Growing_billy_vaccinated_unidentified+"Production-_Epidemiological_Module"."Becoming_adult_nanny_>_12_months_vaccinated_unidentified"+"Production-_Epidemiological_Module".Growing_nanny_vaccinated_unidentified+"Production-_Epidemiological_Module".Becoming_adult_vaccinated_unidentified) |  | CFA/Weeks |  |  |
| Avoidable_vaccination_cost_sheep[HHD_Gender, SR_activity_responsibility] | Unit_vaccination_cost_sheep*("Production-_Epidemiological_Module".Growing_Ram_vaccinated_but_unidentified+"Production-_Epidemiological_Module".Growing_Ewe_vaccinated_but_unidentified+"Production-_Epidemiological_Module".Becoming_Adult_Ram_vaccinated_but_unidentified+"Production-_Epidemiological_Module".Becoming_Adult_Ewe_vaccinated_unidentified) |  | CFA/Weeks |  |  |
| BCR[HHD_Gender, SR_activity_responsibility] | Total_Revenue/Total_production_cost |  | Dimensionless |  |  |
| Economic_loss_billy[HHD_Gender, SR_activity_responsibility] | Average_price_mature_billy*Average_weight_loss_of_mature_goats_due_to_mortality*("Production-_Epidemiological_Module"."Kid_>3_months_Death"+"Production-_Epidemiological_Module".Young_Billy_Deaths+"Production-_Epidemiological_Module".Grower_Billy_Deaths+"Production-_Epidemiological_Module".Kid_Still_Birth_Death+"Production-_Epidemiological_Module".Adult_billy_Deaths) |  | CFA |  |  |
| Economic_loss_Ewe[HHD_Gender, SR_activity_responsibility] | Average_price_fully_grown_Ewe*Average_weight_loss_of_mature_sheep_due_to_mortality*("Production-_Epidemiological_Module".Young_Ewe_3_to_6_months_Death+"Production-_Epidemiological_Module".Adult_Ewes_Death+"Production-_Epidemiological_Module".Grower_Ewes_6_to_12_months_Death) |  | CFA |  |  |
| Economic_loss_nanny[HHD_Gender, SR_activity_responsibility] | Average_weight_loss_of_mature_goats_due_to_mortality*Average_price_mature_nanny*("Production-_Epidemiological_Module".Young_Nanny_Death+"Production-_Epidemiological_Module"."Adult_Nanny_>12_months_Death"+"Production-_Epidemiological_Module".Grower_Nanny_Death) |  | CFA |  |  |
| Economic_loss_Ram[HHD_Gender, SR_activity_responsibility] | Average_price_mature_Ram*Average_weight_loss_of_mature_sheep_due_to_mortality*("Production-_Epidemiological_Module"."Lamb_>3_months_Death"+"Production-_Epidemiological_Module".Grower_Rams_6_to_12_months_Death+"Production-_Epidemiological_Module".Still_Birth_Ram_Death+"Production-_Epidemiological_Module".Young_Ram_3_to_6_months_Death+"Production-_Epidemiological_Module"."Adult_Ram_>12_months_Death") |  | CFA |  |  |
| Feed_intake_adult_goat[HHD_Gender, SR_activity_responsibility] | Average_weekly_feed_intake_adult_goat[Adult,HHD_Gender,SR_activity_responsibility]*"Production-_Epidemiological_Module"."Total_Adult_goat_>12_months" |  | Kilograms |  |  |
| Feed_intake_adult_sheep[Male_HHD, HHD] | Average_weekly_feed_intake_adult_sheep[Adult,Male_HHD,HHD]*"Production-_Epidemiological_Module"."Total_Adult_>12_months"[Male_HHD,HHD] |  | Kilograms |  |  |
| Feed_intake_adult_sheep[Male_HHD, Spouse] | Average_weekly_feed_intake_adult_sheep[Adult,Male_HHD,Spouse]*"Production-_Epidemiological_Module"."Total_Adult_>12_months"[Male_HHD,Spouse] |  |  |  |  |
| Feed_intake_adult_sheep[Female_HHD, HHD] | Average_weekly_feed_intake_adult_sheep[Adult,Female_HHD,HHD]*"Production-_Epidemiological_Module"."Total_Adult_>12_months"[Female_HHD,HHD] |  |  |  |  |
| Feed_intake_adult_sheep[Female_HHD, Spouse] | Average_weekly_feed_intake_adult_sheep[Adult,Female_HHD,Spouse]*"Production-_Epidemiological_Module"."Total_Adult_>12_months"[Female_HHD,Spouse] |  |  |  |  |
| Feed_intake_grower_goat[HHD_Gender, SR_activity_responsibility] | Average_weekly_feed_intake_grower_goat[Grower,HHD_Gender,SR_activity_responsibility]*"Production-_Epidemiological_Module".Total_Grower_goat_6_to_12_months |  | Kilograms |  |  |
| Feed_intake_grower_sheep[Male_HHD, HHD] | Average_weekly_feed_intake_grower_sheep[Male_HHD,HHD,Grower]*"Production-_Epidemiological_Module".Total_Grower_sheep_6_to_12_months[Male_HHD,HHD] |  | Kilograms |  |  |
| Feed_intake_grower_sheep[Male_HHD, Spouse] | Average_weekly_feed_intake_grower_sheep[Male_HHD,Spouse,Grower]*"Production-_Epidemiological_Module".Total_Grower_sheep_6_to_12_months[Male_HHD,Spouse] |  |  |  |  |
| Feed_intake_grower_sheep[Female_HHD, HHD] | Average_weekly_feed_intake_grower_sheep[Female_HHD,HHD,Grower]*"Production-_Epidemiological_Module".Total_Grower_sheep_6_to_12_months[Female_HHD,HHD] |  |  |  |  |
| Feed_intake_grower_sheep[Female_HHD, Spouse] | Average_weekly_feed_intake_grower_sheep[Female_HHD,Spouse,Grower]*"Production-_Epidemiological_Module".Total_Grower_sheep_6_to_12_months[Female_HHD,Spouse] |  |  |  |  |
| Feed_intake_young_goat[Male_HHD, HHD] | Average_weekly_feed_intake_young_goat[Young,Male_HHD,HHD]*"Production-_Epidemiological_Module".Total_young_goat_3_to_6_months[Male_HHD,HHD] |  | Kilograms |  |  |
| Feed_intake_young_goat[Male_HHD, Spouse] | Average_weekly_feed_intake_young_goat[Young,Male_HHD,Spouse]*"Production-_Epidemiological_Module".Total_young_goat_3_to_6_months[Male_HHD,Spouse] |  |  |  |  |
| Feed_intake_young_goat[Female_HHD, HHD] | Average_weekly_feed_intake_young_goat[Young,Female_HHD,HHD]*"Production-_Epidemiological_Module".Total_young_goat_3_to_6_months[Female_HHD,HHD] |  |  |  |  |
| Feed_intake_young_goat[Female_HHD, Spouse] | Average_weekly_feed_intake_young_goat[Young,Female_HHD,Spouse]*"Production-_Epidemiological_Module".Total_young_goat_3_to_6_months[Female_HHD,Spouse] |  |  |  |  |
| Feed_intake_young_sheep[Male_HHD, HHD] | Average_weekly_feed_intake_level_sheep[Young,Male_HHD,HHD]*"Production-_Epidemiological_Module".Total_Young_sheep_3_to_6_months[Male_HHD,HHD] |  | Kilograms |  |  |
| Feed_intake_young_sheep[Male_HHD, Spouse] | Average_weekly_feed_intake_level_sheep[Young,Male_HHD,Spouse]*"Production-_Epidemiological_Module".Total_Young_sheep_3_to_6_months[Male_HHD,Spouse] |  |  |  |  |
| Feed_intake_young_sheep[Female_HHD, HHD] | Average_weekly_feed_intake_level_sheep[Young,Female_HHD,HHD]*"Production-_Epidemiological_Module".Total_Young_sheep_3_to_6_months[Female_HHD,HHD] |  |  |  |  |
| Feed_intake_young_sheep[Female_HHD, Spouse] | Average_weekly_feed_intake_level_sheep[Young,Female_HHD,Spouse]*"Production-_Epidemiological_Module".Total_Young_sheep_3_to_6_months[Female_HHD,Spouse] |  |  |  |  |
| Gross_Margin[HHD_Gender, SR_activity_responsibility] | Total_Revenue-Total_production_cost |  | CFA |  |  |
| Grower_goat_feeding_cost[HHD_Gender, SR_activity_responsibility] | Feed_intake_grower_goat*Average_weekly_feeding_cost_goat[Grower] |  | CFA/Weeks |  |  |
| Grower_sheep_feeding_cost[HHD_Gender, SR_activity_responsibility] | Feed_intake_grower_sheep*Average_weekly_feeding_cost_sheep[Grower] |  | CFA/Weeks |  |  |
| Interval_of_profit | 52 |  | Weeks |  |  |
| Revenue_adult_sheep_sales[HHD_Gender, SR_activity_responsibility] | (Total_weight_of_adult_ram_sold*Marketing_Module.Sheep_Price_on_the_market)+(Total_weight_of_adult_ewe_sold*Marketing_Module.Sheep_Price_on_the_market)+(Total_weight_of_reserved_adult_ram_sold*Marketing_Module.Sheep_Price_on_the_market) {CFA per week} |  | CFA |  |  |
| Revenue_Goat[HHD_Gender, SR_activity_responsibility] | Revenue_young_goat_sale+Revenue_of_adult_goat_sold |  | CFA |  |  |
| Revenue_of_adult_goat_sold[HHD_Gender, SR_activity_responsibility] | (Total_weight_of_served_adult_billy*Marketing_Module.Goat_Price_on_the_market)+(Total_weight_of_adult_billy_sold*Marketing_Module.Goat_Price_on_the_market)+(Total_weight_culled_adult_nanny_sold*Marketing_Module.Goat_Price_on_the_market) {CFA per week} |  | CFA |  |  |
| Revenue_Sheep[HHD_Gender, SR_activity_responsibility] | Revenue_young_sheep_sales+Revenue_adult_sheep_sales |  | CFA |  |  |
| Revenue_young_goat_sale[HHD_Gender, SR_activity_responsibility] | (Total_weight_of_young_billy_sold*Marketing_Module.Goat_Price_on_the_market) +(Total_weight_of_young_nanny_sold*Marketing_Module.Goat_Price_on_the_market) {CFA per week} |  | CFA |  |  |
| Revenue_young_sheep_sales[HHD_Gender, SR_activity_responsibility] | (Total_weight_of_young_ram_sold*Marketing_Module.Sheep_Price_on_the_market) +(Total_weight_of_young_ewe_sold*Marketing_Module.Sheep_Price_on_the_market) |  | CFA |  |  |
| Time_of_loss_incurred | 1 |  | Weeks |  |  |
| Timing_for_cost | 1 |  | Weeks |  |  |
| Total_Economic_Loss[HHD_Gender, SR_activity_responsibility] | Economic_loss_Ram+Economic_loss_billy+Economic_loss_Ewe+Economic_loss_nanny+((Avoidable_vaccination_cost_sheep+Avoidable_vaccination_cost_goat)*Time_of_loss_incurred) |  | CFA |  |  |
| Total_goat_death[HHD_Gender, SR_activity_responsibility] | "Production-_Epidemiological_Module".Adult_billy_Deaths+"Production-_Epidemiological_Module"."Kid_>3_months_Death"+"Production-_Epidemiological_Module".Kid_Still_Birth_Death+"Production-_Epidemiological_Module".Young_Nanny_Death+"Production-_Epidemiological_Module".Young_Billy_Deaths+"Production-_Epidemiological_Module".Grower_Nanny_Death+"Production-_Epidemiological_Module".Grower_Billy_Deaths+"Production-_Epidemiological_Module"."Adult_Nanny_>12_months_Death" |  | Goat |  |  |
| Total_goat_feeding_cost[HHD_Gender, SR_activity_responsibility] | Young_goat_feeding_cost+Adult_goat_feeding_cost+Grower_goat_feeding_cost |  | CFA/Weeks |  |  |
| Total_production_cost[HHD_Gender, SR_activity_responsibility] | (Total_sheep_feeding_cost+Total_goat_feeding_cost+Vaccination_cost_goat+Vaccination_cost_sheep)*Timing_for_cost |  | CFA |  |  |
| Total_Revenue[HHD_Gender, SR_activity_responsibility] | Revenue_Sheep+Revenue_Goat |  | CFA |  |  |
| Total_sheep_death[HHD_Gender, SR_activity_responsibility] | "Production-_Epidemiological_Module".Grower_Rams_6_to_12_months_Death+"Production-_Epidemiological_Module".Still_Birth_Ram_Death+"Production-_Epidemiological_Module"."Adult_Ram_>12_months_Death"+"Production-_Epidemiological_Module"."Lamb_>3_months_Death"+"Production-_Epidemiological_Module".Young_Ram_3_to_6_months_Death+"Production-_Epidemiological_Module".Young_Ewe_3_to_6_months_Death+"Production-_Epidemiological_Module".Adult_Ewes_Death+"Production-_Epidemiological_Module".Grower_Ewes_6_to_12_months_Death |  | Sheep |  |  |
| Total_sheep_feeding_cost[HHD_Gender, SR_activity_responsibility] | Young_sheep_feeding_cost+Adult_sheep_feeding_cost+Grower_sheep_feeding_cost |  | CFA/Weeks |  |  |
| Total_weight_culled_adult_nanny_sold[HHD_Gender, SR_activity_responsibility] | "Production-_Epidemiological_Module".Sold_Cull_Adult_nanny*Average_weight_gained_goat[Adult,HHD_Gender,SR_activity_responsibility] {kg per weeks} |  | Kilograms |  |  |
| Total_weight_of_adult_billy_sold[HHD_Gender, SR_activity_responsibility] | "Production-_Epidemiological_Module".Adult_billy_sold*Average_weight_gained_goat[Adult,HHD_Gender,SR_activity_responsibility] {kg per weeks} |  | Kilograms |  |  |
| Total_weight_of_adult_ewe_sold[HHD_Gender, SR_activity_responsibility] | ("Production-_Epidemiological_Module".Sold_Cull_Adult_Ewe+"Production-_Epidemiological_Module"."Sold_Adult_Ewe_>12_months")*Average_weight_for_mature_sheep[Adult,HHD_Gender,SR_activity_responsibility] {kg per week} |  | Kilograms |  |  |
| Total_weight_of_adult_ram_sold[HHD_Gender, SR_activity_responsibility] | "Production-_Epidemiological_Module".Sold_Adult_ram*Average_weight_for_mature_sheep[Adult,HHD_Gender,SR_activity_responsibility] {kg per week} |  | Kilograms |  |  |
| Total_weight_of_reserved_adult_ram_sold[HHD_Gender, SR_activity_responsibility] | "Production-_Epidemiological_Module".Sold_Reserved_Adult_Ram*Average_weight_for_mature_sheep[Adult,HHD_Gender,SR_activity_responsibility] {kg per week} |  | Kilograms |  |  |
| Total_weight_of_served_adult_billy[HHD_Gender, SR_activity_responsibility] | "Production-_Epidemiological_Module".Adult_Billy_reserved_sold*Average_weight_gained_goat[Adult,HHD_Gender,SR_activity_responsibility] {kg per week} |  | Kilograms |  |  |
| Total_weight_of_young_billy_sold[HHD_Gender, SR_activity_responsibility] | "Production-_Epidemiological_Module".Young_billy_Sold*Average_weight_gained_goat[Young,HHD_Gender,SR_activity_responsibility] {kg per week} |  | Kilograms |  |  |
| Total_weight_of_young_ewe_sold[HHD_Gender, SR_activity_responsibility] | "Production-_Epidemiological_Module".Sold_Young_Ewe*Average_weight_for_mature_sheep[Young,HHD_Gender,SR_activity_responsibility] {kg per week} |  | Kilograms |  |  |
| Total_weight_of_young_nanny_sold[HHD_Gender, SR_activity_responsibility] | "Production-_Epidemiological_Module".Young_nanny_Sold*Average_weight_gained_goat[Young,HHD_Gender,SR_activity_responsibility]{kg per week} |  | Kilograms |  |  |
| Total_weight_of_young_ram_sold[HHD_Gender, SR_activity_responsibility] | ("Production-_Epidemiological_Module".Sold_Young_Ram_3_to_6_months_natural_recovered+"Production-_Epidemiological_Module".Sold_Young_Ram_3_to_6_recovered)*Average_weight_for_mature_sheep[Young,HHD_Gender,SR_activity_responsibility] |  | Kilograms |  |  |
| Unit_vaccination_cost_goat[HHD_Gender, SR_activity_responsibility] | Actual_vaccination_cost_goat-(Actual_vaccination_cost_goat*(Policy_Module.Proportion_covered_by_subsidy*Policy_Module.Government_subsidy)) |  | CFA per goat |  |  |
| Unit_vaccination_cost_sheep[HHD_Gender, SR_activity_responsibility] | Actual_vaccination_cost_sheep-(Actual_vaccination_cost_sheep*(Policy_Module.Proportion_covered_by_subsidy*Policy_Module.Government_subsidy)) {Tago et al., 2017 Vaccin cost - Min $0.24 Mean= $0.34, Max= $0.54} |  | CFA per sheep |  |  |
| Vaccination_cost_goat[HHD_Gender, SR_activity_responsibility] | (Unit_vaccination_cost_goat*(Disease_Control_Module.Young_nanny_3_to_6_months_vaccination_rate+Disease_Control_Module.Young_billy_3_to_6_months_vaccination_rate+Disease_Control_Module.Grower_billy_6_to_12_months_vaccination_rate+Disease_Control_Module.Grower_nanny_6_to_12_months_vaccination_rate+Disease_Control_Module."Adult_billy_>12_months_vaccination_rate"+Disease_Control_Module."Adult_nanny_>_12_months_vaccination_rate"))+Avoidable_vaccination_cost_goat |  | CFA/Weeks |  |  |
| Vaccination_cost_sheep[HHD_Gender, SR_activity_responsibility] | ((Disease_Control_Module.Young_Ram_3_to_6_months_Vaccination_rate+Disease_Control_Module.Young_Ewe_3_to_6_months_Vaccination_rate+Disease_Control_Module.Grower_Ewes_6_to_12_months_vaccination_rate+Disease_Control_Module.Grower_Rams_6_to_12_months_vaccination_rate+Disease_Control_Module."Adult_Ewe_>_12_months_vaccination_rate"+Disease_Control_Module."Adult_Ram_>12_months_vaccination_rate")*Unit_vaccination_cost_sheep)+Avoidable_vaccination_cost_sheep |  | CFA/Weeks |  |  |
| Young_goat_feeding_cost[HHD_Gender, SR_activity_responsibility] | Feed_intake_young_goat*Average_weekly_feeding_cost_goat[Young] |  | CFA/Weeks |  |  |
| Young_sheep_feeding_cost[HHD_Gender, SR_activity_responsibility] | Feed_intake_young_sheep*Average_weekly_feeding_cost_sheep[Young] {CFA} |  | CFA/Weeks |  |  |
| Marketing_Module: | | | | | |
| Goat_Price_on_the_market(t) | Goat_Price_on_the_market(t - dt) + (Change_in_goat_price) * dt | INIT Goat_Price_on_the_market = 2500{HH survey- selling price divided by 40 kg} | CFA |  | NON-NEGATIVE |
| Inventory_Goat_on_market(t) | Inventory_Goat_on_market(t - dt) + (Goat_supply_to_market + Goat_import - "Slaughtered_goat_for_in-country_consumption") * dt | INIT Inventory_Goat_on_market = 6232388/52{FAOSTAT, 2019} | Goat |  | NON-NEGATIVE |
| Inventory_sheep_on_market(t) | Inventory_sheep_on_market(t - dt) + (Sheep_supply_to_market + Sheep_import - "Slaughtered_sheep_for_in-country_consumption") * dt | INIT Inventory_sheep_on_market = 5456574/52{FAOSTAT, 2019} | Sheep |  | NON-NEGATIVE |
| Population_in_Senegal(t) | Population_in_Senegal(t - dt) + (Change_in_population) * dt | INIT Population_in_Senegal = 17196308 {Worldbank data, 2021} | People |  | NON-NEGATIVE |
| Sheep_Price_on_the_market(t) | Sheep_Price_on_the_market(t - dt) + (Change_in_sheep_price) * dt | INIT Sheep_Price_on_the_market = 2500{HH survey- selling price divided by 40 kg} | CFA |  | NON-NEGATIVE |
| Change_in_goat_price | (Goat_Price_on_the_market-Desired_goat_price)/Goat_price_change_delay |  | CFA/Weeks |  |  |
| Change_in_population | Population_in_Senegal*Fractional_population_growth_rate |  | People/Weeks |  | UNIFLOW |
| Change_in_sheep_price | (Sheep_Price_on_the_market-Desired_sheep_price)/Sheep_price_change_delay |  | CFA/Weeks |  |  |
| Goat_import | Inventory_Goat_on_market*Goat_import_rate |  | Goat/Weeks |  | UNIFLOW |
| Goat_supply_to_market | Average_goat_from_individual_farm_household*Number_of_goat_rearing_farm_households_in_Senegal |  | Goat/Weeks |  | UNIFLOW |
| Sheep_import | Inventory_sheep_on_market*Sheep_import_rate |  | Sheep per week |  | UNIFLOW |
| Sheep_supply_to_market | Average_sheep_from_individual_farm_household*Number_of_sheep_rearing_farm_households_in_Senegal |  | Sheep per week |  | UNIFLOW |
| "Slaughtered_goat_for_in-country_consumption" | Inventory_Goat_on_market*National_average_goat_slaughtering_rate |  | Goat/Weeks |  | UNIFLOW |
| "Slaughtered_sheep_for_in-country_consumption" | Inventory_sheep_on_market*National_average_sheep_slaughtering_rate |  | Sheep per week |  | UNIFLOW |
| Average_goat_Carcass_Weight | MEAN(Economic_Module.Average_weight_gained_goat) |  | kg per Goat |  |  |
| Average_goat_from_individual_farm_household | (MEAN("Production-_Epidemiological_Module".Young_nanny_Sold)+MEAN("Production-_Epidemiological_Module".Adult_billy_sold)+MEAN("Production-_Epidemiological_Module".Sold_Cull_Adult_nanny)+MEAN("Production-_Epidemiological_Module".Adult_Billy_reserved_sold)+MEAN("Production-_Epidemiological_Module".Young_billy_Sold))/(Number_of_farm_households_selling_goat_weekly*Duration_of_goat_sales_contribution) |  | Goat per household per weeks |  |  |
| Average_household_size | 7 {worldbank data} |  | People per household |  |  |
| Average_Sheep_Carcass_Weight | MEAN(Economic_Module.Average_weight_for_mature_sheep) |  | kg per sheep |  |  |
| Average_sheep_from_individual_farm_household | (MEAN("Production-_Epidemiological_Module"."Sold_Adult_Ewe_>12_months")+MEAN("Production-_Epidemiological_Module".Sold_Cull_Adult_Ewe)+MEAN("Production-_Epidemiological_Module".Sold_Reserved_Adult_Ram)+MEAN("Production-_Epidemiological_Module".Sold_Young_Ewe)+MEAN("Production-_Epidemiological_Module".Sold_Adult_ram) +MEAN("Production-_Epidemiological_Module".Sold_Young_Ram_3_to_6_months_natural_recovered) +MEAN("Production-_Epidemiological_Module".Sold_Young_Ram_3_to_6_recovered))/(Duration_of_sheep_sales_contribution*Number_of_farm_households_selling_sheep_weekly) |  | Sheep per household per weeks |  |  |
| Desired_Goat_Inventory | National_demand_for_slaughtered_goat*Desired_goat_Inventory_Coverage |  | Goat |  |  |
| Desired_goat_Inventory_Coverage | 1 |  | Weeks |  |  |
| Desired_goat_price | Goat_Price_on_the_market*Effect_on_goat_price |  | CFA |  |  |
| Desired_Sheep_Inventory | National_demand_for_slaughtered_sheep*Desired_Sheep_Inventory_Coverage |  | Sheep |  |  |
| Desired_Sheep_Inventory_Coverage | 1 |  | Weeks |  |  |
| Desired_sheep_price | Sheep_Price_on_the_market*Effect_on_sheep_price |  | CFA |  |  |
| Duration_of_goat_sales_contribution | 208 |  | Weeks |  |  |
| Duration_of_sheep_sales_contribution | 52 |  | Weeks |  |  |
| Effect_on_goat_price | Goat_Inventory_ratio^Elasticity_of_goat_inventory_ratio |  | Dimensionless |  |  |
| Effect_on_sheep_price | Sheep_Inventory_ratio^Elasticity_of_sheep_inventory_ratio |  | Dimensionless |  |  |
| Elasticity_of_demand_for_goat | -0.61{Femenia, 2019} |  | Dimensionless |  |  |
| Elasticity_of_demand_for_mutton | -0.61{Femenia, 2019} |  | Dimensionless |  |  |
| Elasticity_of_goat_inventory_ratio | -0.01 {Parameter for sensitivity analysis} |  | Dimensionless |  |  |
| Elasticity_of_sheep_inventory_ratio | -0.01 {Parameter for sensitivity analysis} |  | Dimensionless |  |  |
| Estimated_per_capita_consumption_goat_meat | ("Slaughtered_goat_for_in-country_consumption"*Average_goat_Carcass_Weight)/Population_in_Senegal |  | Kilograms/(Persons*Weeks) |  |  |
| Estimated_per_capita_consumption_of_sheep_meat | ("Slaughtered_sheep_for_in-country_consumption"*Average_Sheep_Carcass_Weight)/Population_in_Senegal |  | Kilograms/(Persons*Weeks) |  |  |
| Fractional_population_growth_rate | 0.027/52 {World bank, 2021} |  | Per Week |  |  |
| Goat_demand_intercept | LN((Population_in_Senegal*Weekly_per_capita_consumption_goat*Market_share_for_domestic_goat)/(Goat_Price_on_the_market^Elasticity_of_demand_for_goat)) |  | kg per week |  |  |
| Goat_import_rate | 0.069/52{AfDB} |  | Per Week |  |  |
| Goat_Inventory_ratio | Inventory_Goat_on_market/Desired_Goat_Inventory |  | Dimensionless |  |  |
| Goat_price_change_delay | 52 |  | Dimensionless |  |  |
| Market_share_for_domestic_goat | 0.9 |  | Dimensionless |  |  |
| Market_share_for_domestic_mutton | 0.9 |  | Dimensionless |  |  |
| Mutton_demand_intercept | LN((Population_in_Senegal*Weekly_per_capita_consumption_Mutton*Market_share_for_domestic_mutton)/(Sheep_Price_on_the_market^Elasticity_of_demand_for_mutton)) |  | kg per week |  |  |
| National_average_goat_slaughtering_rate | 0.214 {AfDB Data} |  | Per Week |  |  |
| National_average_sheep_slaughtering_rate | 0.293 {AfDB Data from 2010 to 2020} |  | Per Week |  |  |
| National_demand_for_slaughtered_goat | National_Weekly_Demand_for_goat/Average_goat_Carcass_Weight |  | Goat/Weeks |  |  |
| National_demand_for_slaughtered_sheep | National_Weekly_Demand_for_Mutton/Average_Sheep_Carcass_Weight |  | Sheep per week |  |  |
| National_Weekly_Demand_for_goat | EXP(Goat_demand_intercept)*(Goat_Price_on_the_market^Elasticity_of_demand_for_goat) |  | kg per week |  |  |
| National_Weekly_Demand_for_Mutton | EXP(Mutton_demand_intercept)*(Sheep_Price_on_the_market^Elasticity_of_demand_for_mutton) |  | kg per week |  |  |
| Number_of_farm_households_selling_goat_weekly | 1 |  | Household |  |  |
| Number_of_farm_households_selling_sheep_weekly | 1 |  | Household |  |  |
| Number_of_goat_rearing_farm_households_in_Senegal | (Population_in_Senegal/Average_household_size)*Proportion_goat_rearing_farm_household |  | Household |  |  |
| Number_of_sheep_rearing_farm_households_in_Senegal | (Population_in_Senegal/Average_household_size)*Proportion_sheep_rearing_Farm_household |  | Household |  |  |
| Proportion_goat_rearing_farm_household | 0.295 {Habanabakize et al., 2022} |  | Dimensionless |  |  |
| Proportion_sheep_rearing_Farm_household | 0.295 {Habanabakize et al., 2022} |  | Dimensionless |  |  |
| Sheep_import_rate | 0.069/52{AfDB Data from 2010 to 2020} |  | Per Week |  |  |
| Sheep_Inventory_ratio | Inventory_sheep_on_market/Desired_Sheep_Inventory |  | Dimensionless |  |  |
| Sheep_price_change_delay | 52 |  | Dimensionless |  |  |
| Weekly_per_capita_consumption_goat | 2/52 {yet to be confirmed} |  | kg per week per person |  |  |
| Weekly_per_capita_consumption_Mutton | 2/52 {yet to be confirmed} |  | kg per week per person |  |  |
| Policy_Module: | | | | | |
| Government_subsidy[HHD_Gender, SR_activity_responsibility] | 0 |  | Dimensionless |  |  |
| Government_subsidy_cost_for_vaccination_of_goats | (MEAN(Proportion_covered_by_subsidy)*MEAN(Government_subsidy))*MEAN(Economic_Module.Actual_vaccination_cost_goat)*Marketing_Module.Number_of_goat_rearing_farm_households_in_Senegal*Marketing_Module.Average_goat_from_individual_farm_household |  | CFA/Weeks |  |  |
| Government_subsidy_cost_for_vaccination_of_sheep | (MEAN(Proportion_covered_by_subsidy)*MEAN(Government_subsidy))*MEAN(Economic_Module.Actual_vaccination_cost_sheep)*Marketing_Module.Number_of_sheep_rearing_farm_households_in_Senegal*Marketing_Module.Average_sheep_from_individual_farm_household |  | CFA/Weeks |  |  |
| Proportion_covered_by_subsidy[HHD_Gender, SR_activity_responsibility] | 0 |  | Dimensionless |  |  |
| Total_cost_of_government_subsidy_for_PPR_Control | Government_subsidy_cost_for_vaccination_of_sheep+Government_subsidy_cost_for_vaccination_of_goats |  | CFA/Weeks |  |  |
| "Production-_Epidemiological_Module": | | | | | |
| "Adult_billy_>_12_months_susceptible"[HHD_Gender, SR_activity_responsibility](t) | "Adult_billy_>_12_months_susceptible"[HHD_Gender, SR_activity_responsibility](t - dt) + ( - "Adult_billy_>_12_months_infection_rate"[HHD_Gender, SR_activity_responsibility] - "Adult_billy_>12_months_vaccinating"[HHD_Gender, SR_activity_responsibility] - Adult_billy_stolen[HHD_Gender, SR_activity_responsibility]) * dt | INIT "Adult_billy_>_12_months_susceptible"[HHD_Gender, SR_activity_responsibility] = TRIANGULAR(1, 2, 5) {HH survey data} | Goat |  | NON-NEGATIVE |
| "Adult_billy_>12_months_infected"[HHD_Gender, SR_activity_responsibility](t) | "Adult_billy_>12_months_infected"[HHD_Gender, SR_activity_responsibility](t - dt) + ("Adult_billy_>_12_months_infection_rate"[HHD_Gender, SR_activity_responsibility] - Adult_billy_Dying[HHD_Gender, SR_activity_responsibility] - "Adult_billy_>_12_months_recovery_rate"[HHD_Gender, SR_activity_responsibility]) * dt | INIT "Adult_billy_>12_months_infected"[HHD_Gender, SR_activity_responsibility] = 2 | Goat |  | NON-NEGATIVE |
| "Adult_billy_>12_months_recovered"[HHD_Gender, SR_activity_responsibility](t) | "Adult_billy_>12_months_recovered"[HHD_Gender, SR_activity_responsibility](t - dt) + ("Adult_billy_>_12_months_recovery_rate"[HHD_Gender, SR_activity_responsibility] + Adult_billy_vaccinated_becoming_recovered[HHD_Gender, SR_activity_responsibility] + Billy_received_from_external_sources[HHD_Gender, SR_activity_responsibility] - Selling_Adult_billy[HHD_Gender, SR_activity_responsibility] - HH_consumption_billy[HHD_Gender, SR_activity_responsibility] - Reserved_billy_for_breeding[HHD_Gender, SR_activity_responsibility]) * dt | INIT "Adult_billy_>12_months_recovered"[HHD_Gender, SR_activity_responsibility] = 0 | Goat |  | NON-NEGATIVE |
| "Adult_billy_>12_months_vaccinated"[HHD_Gender, SR_activity_responsibility](t) | "Adult_billy_>12_months_vaccinated"[HHD_Gender, SR_activity_responsibility](t - dt) + ("Adult_billy_>12_months_vaccinating"[HHD_Gender, SR_activity_responsibility] - Adult_billy_vaccinated_becoming_recovered[HHD_Gender, SR_activity_responsibility]) * dt | INIT "Adult_billy_>12_months_vaccinated"[HHD_Gender, SR_activity_responsibility] = 0 | Goat |  | NON-NEGATIVE |
| Adult_billy_Deaths[HHD_Gender, SR_activity_responsibility](t) | Adult_billy_Deaths[HHD_Gender, SR_activity_responsibility](t - dt) + (Adult_billy_Dying[HHD_Gender, SR_activity_responsibility]) * dt | INIT Adult_billy_Deaths[HHD_Gender, SR_activity_responsibility] = 0 | Goat |  | NON-NEGATIVE |
| Adult_Billy_reserved_sold[HHD_Gender, SR_activity_responsibility](t) | Adult_Billy_reserved_sold[HHD_Gender, SR_activity_responsibility](t - dt) + (Selling_of_adult_billy_reserved_for_breeding[HHD_Gender, SR_activity_responsibility]) * dt | INIT Adult_Billy_reserved_sold[HHD_Gender, SR_activity_responsibility] = 0 | Goat |  | NON-NEGATIVE |
| Adult_billy_sold[HHD_Gender, SR_activity_responsibility](t) | Adult_billy_sold[HHD_Gender, SR_activity_responsibility](t - dt) + (Selling_Adult_billy[HHD_Gender, SR_activity_responsibility]) * dt | INIT Adult_billy_sold[HHD_Gender, SR_activity_responsibility] = 0 | Goat |  | NON-NEGATIVE |
| Adult_Buck_for_breeding[HHD_Gender, SR_activity_responsibility](t) | Adult_Buck_for_breeding[HHD_Gender, SR_activity_responsibility](t - dt) + (Becoming_Adult_billy_natural_recovered[HHD_Gender, SR_activity_responsibility] + "Becoming_adult_billy_>_12_months_vaccinated_identified"[HHD_Gender, SR_activity_responsibility] + Reserved_billy_for_breeding[HHD_Gender, SR_activity_responsibility] - Selling_of_adult_billy_reserved_for_breeding[HHD_Gender, SR_activity_responsibility]) * dt | INIT Adult_Buck_for_breeding[HHD_Gender, SR_activity_responsibility] = 1 | Goat |  | NON-NEGATIVE |
| "Adult_Ewe_>_12_months_susceptible"[HHD_Gender, SR_activity_responsibility](t) | "Adult_Ewe_>_12_months_susceptible"[HHD_Gender, SR_activity_responsibility](t - dt) + ( - "Adult_Ewe_>12_months_infection_rate"[HHD_Gender, SR_activity_responsibility] - "Adult_Ewes_>12_months_vaccinating"[HHD_Gender, SR_activity_responsibility] - Adult_Ewe_stolen[HHD_Gender, SR_activity_responsibility]) * dt | INIT "Adult_Ewe_>_12_months_susceptible"[HHD_Gender, SR_activity_responsibility] = TRIANGULAR(0, 1, 15){HH survey data} | Sheep |  | NON-NEGATIVE |
| "Adult_Ewe_>12_months"[HHD_Gender, SR_activity_responsibility](t) | "Adult_Ewe_>12_months"[HHD_Gender, SR_activity_responsibility](t - dt) + (Becoming_Adult_Ewe_Vaccinated_identified[HHD_Gender, SR_activity_responsibility] + Breeding_Ewes_natural_recovered[HHD_Gender, SR_activity_responsibility] + Becoming_Adult_Ewe_natural_recovered[HHD_Gender, SR_activity_responsibility] - Breeding_Ewes_vaccine_antibodies[HHD_Gender, SR_activity_responsibility] - "Adult_Ewe_>12_months_selling"[HHD_Gender, SR_activity_responsibility]) * dt | INIT "Adult_Ewe_>12_months"[HHD_Gender, SR_activity_responsibility] = 0 | Sheep |  | NON-NEGATIVE |
| "Adult_Ewe_>12_months_infected"[HHD_Gender, SR_activity_responsibility](t) | "Adult_Ewe_>12_months_infected"[HHD_Gender, SR_activity_responsibility](t - dt) + ("Adult_Ewe_>12_months_infection_rate"[HHD_Gender, SR_activity_responsibility] - "Adult_Ewe_>_12_months_recovery_rate"[HHD_Gender, SR_activity_responsibility] - Adult_ewes_dying[HHD_Gender, SR_activity_responsibility]) * dt | INIT "Adult_Ewe_>12_months_infected"[HHD_Gender, SR_activity_responsibility] = 0 | Sheep |  | NON-NEGATIVE |
| "Adult_Ewe_>12_months_recovered"[HHD_Gender, SR_activity_responsibility](t) | "Adult_Ewe_>12_months_recovered"[HHD_Gender, SR_activity_responsibility](t - dt) + ("Adult_Ewe_>_12_months_recovery_rate"[HHD_Gender, SR_activity_responsibility] + "Adult_Ewes_>12_months_vaccinating"[HHD_Gender, SR_activity_responsibility] - Breeding_Ewes_natural_recovered[HHD_Gender, SR_activity_responsibility]) * dt | INIT "Adult_Ewe_>12_months_recovered"[HHD_Gender, SR_activity_responsibility] = 0 | Sheep |  | NON-NEGATIVE |
| Adult_Ewes_Death[HHD_Gender, SR_activity_responsibility](t) | Adult_Ewes_Death[HHD_Gender, SR_activity_responsibility](t - dt) + (Adult_ewes_dying[HHD_Gender, SR_activity_responsibility]) * dt | INIT Adult_Ewes_Death[HHD_Gender, SR_activity_responsibility] = 0 | Sheep |  | NON-NEGATIVE |
| "Adult_nanny_>_12_months_susceptible"[HHD_Gender, SR_activity_responsibility](t) | "Adult_nanny_>_12_months_susceptible"[HHD_Gender, SR_activity_responsibility](t - dt) + ( - "Adult_nanny_>_12_months_infection_rate"[HHD_Gender, SR_activity_responsibility] - "Adult_nanny_>12_months_vaccinating"[HHD_Gender, SR_activity_responsibility] - Adult_nanny_stolen[HHD_Gender, SR_activity_responsibility]) * dt | INIT "Adult_nanny_>_12_months_susceptible"[HHD_Gender, SR_activity_responsibility] = TRIANGULAR(1, 2, 5) {HH survey data} | Goat |  | NON-NEGATIVE |
| "Adult_Nanny_>12_months_Death"[HHD_Gender, SR_activity_responsibility](t) | "Adult_Nanny_>12_months_Death"[HHD_Gender, SR_activity_responsibility](t - dt) + ("Adult_nanny_>_12_months_dying"[HHD_Gender, SR_activity_responsibility]) * dt | INIT "Adult_Nanny_>12_months_Death"[HHD_Gender, SR_activity_responsibility] = 0 | Goat |  | NON-NEGATIVE |
| "Adult_nanny_>12_months_infected"[HHD_Gender, SR_activity_responsibility](t) | "Adult_nanny_>12_months_infected"[HHD_Gender, SR_activity_responsibility](t - dt) + ("Adult_nanny_>_12_months_infection_rate"[HHD_Gender, SR_activity_responsibility] - "Adult_nanny_>_12_months_recovery_rate"[HHD_Gender, SR_activity_responsibility] - "Adult_nanny_>_12_months_dying"[HHD_Gender, SR_activity_responsibility]) * dt | INIT "Adult_nanny_>12_months_infected"[HHD_Gender, SR_activity_responsibility] = 1 | Goat |  | NON-NEGATIVE |
| "Adult_nanny_>12_months_recovered"[HHD_Gender, SR_activity_responsibility](t) | "Adult_nanny_>12_months_recovered"[HHD_Gender, SR_activity_responsibility](t - dt) + ("Adult_nanny_>_12_months_recovery_rate"[HHD_Gender, SR_activity_responsibility] + Nanny_received_from_external_sources[HHD_Gender, SR_activity_responsibility] - Breeding_nannies[HHD_Gender, SR_activity_responsibility]) * dt | INIT "Adult_nanny_>12_months_recovered"[HHD_Gender, SR_activity_responsibility] = 0 | Goat |  | NON-NEGATIVE |
| "Adult_nanny_>12_months_vaccinated"[HHD_Gender, SR_activity_responsibility](t) | "Adult_nanny_>12_months_vaccinated"[HHD_Gender, SR_activity_responsibility](t - dt) + ("Adult_nanny_>12_months_vaccinating"[HHD_Gender, SR_activity_responsibility] - Adult_nanny_vaccinated_becoming_recovered[HHD_Gender, SR_activity_responsibility]) * dt | INIT "Adult_nanny_>12_months_vaccinated"[HHD_Gender, SR_activity_responsibility] = 0 | Goat |  | NON-NEGATIVE |
| "Adult_Ram_>_12_months_infected"[HHD_Gender, SR_activity_responsibility](t) | "Adult_Ram_>_12_months_infected"[HHD_Gender, SR_activity_responsibility](t - dt) + ("Adult_Ram_>12_months_infection_rate"[HHD_Gender, SR_activity_responsibility] - Adult_Ram_Deaths[HHD_Gender, SR_activity_responsibility] - "Adult_Ram_>_12_months_natural_recovery_rate"[HHD_Gender, SR_activity_responsibility]) * dt | INIT "Adult_Ram_>_12_months_infected"[HHD_Gender, SR_activity_responsibility] = 2 | Sheep |  | NON-NEGATIVE |
| "Adult_Ram_>_12_months_vaccinated"[HHD_Gender, SR_activity_responsibility](t) | "Adult_Ram_>_12_months_vaccinated"[HHD_Gender, SR_activity_responsibility](t - dt) + ("Adult_Ram_>12_months_vaccinating"[HHD_Gender, SR_activity_responsibility] - "Adult_Ram_>_12_months_vaccinated_recovery_rate"[HHD_Gender, SR_activity_responsibility]) * dt | INIT "Adult_Ram_>_12_months_vaccinated"[HHD_Gender, SR_activity_responsibility] = 0 | Sheep |  | NON-NEGATIVE |
| "Adult_Ram_>12_months_Death"[HHD_Gender, SR_activity_responsibility](t) | "Adult_Ram_>12_months_Death"[HHD_Gender, SR_activity_responsibility](t - dt) + (Adult_Ram_Deaths[HHD_Gender, SR_activity_responsibility]) * dt | INIT "Adult_Ram_>12_months_Death"[HHD_Gender, SR_activity_responsibility] = 0 | Sheep |  | NON-NEGATIVE |
| "Adult_Ram_>12_months_Recovered"[HHD_Gender, SR_activity_responsibility](t) | "Adult_Ram_>12_months_Recovered"[HHD_Gender, SR_activity_responsibility](t - dt) + (Becoming_Adult_Ram_vaccinated_identified[HHD_Gender, SR_activity_responsibility] + "Adult_Ram_>_12_months_natural_recovery_rate"[HHD_Gender, SR_activity_responsibility] + "Adult_Ram_>_12_months_vaccinated_recovery_rate"[HHD_Gender, SR_activity_responsibility] + Ram_received_from_external_sources[HHD_Gender, SR_activity_responsibility] + Becoming_Adult_Ram_natural_recovery[HHD_Gender, SR_activity_responsibility] - HH_consumption_Ram[HHD_Gender, SR_activity_responsibility] - Reserved_ram_for_breeding[HHD_Gender, SR_activity_responsibility] - Selling_Adult_Ram[HHD_Gender, SR_activity_responsibility]) * dt | INIT "Adult_Ram_>12_months_Recovered"[HHD_Gender, SR_activity_responsibility] = 0 | Sheep |  | NON-NEGATIVE |
| "Adult_Ram_>12_months_susceptible"[HHD_Gender, SR_activity_responsibility](t) | "Adult_Ram_>12_months_susceptible"[HHD_Gender, SR_activity_responsibility](t - dt) + ( - "Adult_Ram_>12_months_infection_rate"[HHD_Gender, SR_activity_responsibility] - "Adult_Ram_>12_months_vaccinating"[HHD_Gender, SR_activity_responsibility] - "Adult_Ram_>12_months_stolen"[HHD_Gender, SR_activity_responsibility]) * dt | INIT "Adult_Ram_>12_months_susceptible"[HHD_Gender, SR_activity_responsibility] = 5 {HH survey data} | Sheep |  | NON-NEGATIVE |
| Adult_Ram_for_breeding[HHD_Gender, SR_activity_responsibility](t) | Adult_Ram_for_breeding[HHD_Gender, SR_activity_responsibility](t - dt) + (Reserved_ram_for_breeding[HHD_Gender, SR_activity_responsibility] - Selling_Reserved_Adult_Ram[HHD_Gender, SR_activity_responsibility]) * dt | INIT Adult_Ram_for_breeding[HHD_Gender, SR_activity_responsibility] = 1 | Sheep |  | NON-NEGATIVE |
| Bred_back_Ewes[HHD_Gender, SR_activity_responsibility](t) | Bred_back_Ewes[HHD_Gender, SR_activity_responsibility](t - dt) + (Breeding_Ewes_vaccine_antibodies[HHD_Gender, SR_activity_responsibility] + Ewe_received_from_external_sources[HHD_Gender, SR_activity_responsibility] - HH_Ewe_Consumption[HHD_Gender, SR_activity_responsibility] - Selling_Cull_Adult_Ewe[HHD_Gender, SR_activity_responsibility]) * dt | INIT Bred_back_Ewes[HHD_Gender, SR_activity_responsibility] = 1 | Sheep |  | NON-NEGATIVE |
| Bred_back_Nanny[HHD_Gender, SR_activity_responsibility](t) | Bred_back_Nanny[HHD_Gender, SR_activity_responsibility](t - dt) + (Breeding_nannies[HHD_Gender, SR_activity_responsibility] + Adult_nanny_vaccinated_becoming_recovered[HHD_Gender, SR_activity_responsibility] + Adulting_nanny_becoming_vulnerable[HHD_Gender, SR_activity_responsibility] + "Becoming_adult_nanny_>_12_months_vaccinated"[HHD_Gender, SR_activity_responsibility] - HH_nanny_Consumption[HHD_Gender, SR_activity_responsibility] - Cull_selling_adult_nanny[HHD_Gender, SR_activity_responsibility]) * dt | INIT Bred_back_Nanny[HHD_Gender, SR_activity_responsibility] = 1 | Goat |  | NON-NEGATIVE |
| Goat_Gestation_Delay[HHD_Gender, SR_activity_responsibility](t) | Goat_Gestation_Delay[HHD_Gender, SR_activity_responsibility](t - dt) + (Kid_Breeding_rate[HHD_Gender, SR_activity_responsibility] - Kid_Still_born_rate[HHD_Gender, SR_activity_responsibility] - Kidding[HHD_Gender, SR_activity_responsibility]) * dt | INIT Goat_Gestation_Delay[HHD_Gender, SR_activity_responsibility] = 0 | Goat |  | NON-NEGATIVE |
| Grower_billy_6_to_12_months_infected[HHD_Gender, SR_activity_responsibility](t) | Grower_billy_6_to_12_months_infected[HHD_Gender, SR_activity_responsibility](t - dt) + (Grower_billy_6_to_12_months_infection_rate[HHD_Gender, SR_activity_responsibility] - Grower_billy_6_to_12_months_recovery_rate[HHD_Gender, SR_activity_responsibility] - Growers_billy_Dying[HHD_Gender, SR_activity_responsibility]) * dt | INIT Grower_billy_6_to_12_months_infected[HHD_Gender, SR_activity_responsibility] = 2 | Goat |  | NON-NEGATIVE |
| Grower_billy_6_to_12_months_recovered[HHD_Gender, SR_activity_responsibility](t) | Grower_billy_6_to_12_months_recovered[HHD_Gender, SR_activity_responsibility](t - dt) + (Grower_billy_6_to_12_months_recovery_rate[HHD_Gender, SR_activity_responsibility] - Billy_given_out[HHD_Gender, SR_activity_responsibility] - Becoming_Adult_billy_natural_recovered[HHD_Gender, SR_activity_responsibility]) * dt | INIT Grower_billy_6_to_12_months_recovered[HHD_Gender, SR_activity_responsibility] = 0 | Goat |  | NON-NEGATIVE |
| Grower_billy_6_to_12_months_susceptible[HHD_Gender, SR_activity_responsibility](t) | Grower_billy_6_to_12_months_susceptible[HHD_Gender, SR_activity_responsibility](t - dt) + ( - Grower_billy_6_to_12_months_infection_rate[HHD_Gender, SR_activity_responsibility] - Grower_billy_6_to_12_months_vaccinating[HHD_Gender, SR_activity_responsibility] - Grower_billy_stolen[HHD_Gender, SR_activity_responsibility]) * dt | INIT Grower_billy_6_to_12_months_susceptible[HHD_Gender, SR_activity_responsibility] = TRIANGULAR(1, 2, 5){HH survey data} | Goat |  | NON-NEGATIVE |
| Grower_billy_6_to_12_months_vaccinated[HHD_Gender, SR_activity_responsibility](t) | Grower_billy_6_to_12_months_vaccinated[HHD_Gender, SR_activity_responsibility](t - dt) + (Grower_billy_6_to_12_months_vaccinating[HHD_Gender, SR_activity_responsibility] - Grower_billy_6_to_12_months_vaccinated_recovery_rate[HHD_Gender, SR_activity_responsibility]) * dt | INIT Grower_billy_6_to_12_months_vaccinated[HHD_Gender, SR_activity_responsibility] = 0 | Goat |  | NON-NEGATIVE |
| Grower_billy_6_to_12_months_vaccinated_recovered[HHD_Gender, SR_activity_responsibility](t) | Grower_billy_6_to_12_months_vaccinated_recovered[HHD_Gender, SR_activity_responsibility](t - dt) + (Grower_billy_6_to_12_months_vaccinated_recovery_rate[HHD_Gender, SR_activity_responsibility] + Growing_billy_natural_recovered[HHD_Gender, SR_activity_responsibility] + Growing_billy_vaccinated_identified[HHD_Gender, SR_activity_responsibility] - "Becoming_adult_billy_>_12_months_vaccinated_identified"[HHD_Gender, SR_activity_responsibility] - Becoming_adult_vaccinated_unidentified[HHD_Gender, SR_activity_responsibility]) * dt | INIT Grower_billy_6_to_12_months_vaccinated_recovered[HHD_Gender, SR_activity_responsibility] = 0 | Goat |  | NON-NEGATIVE |
| Grower_Billy_Deaths[HHD_Gender, SR_activity_responsibility](t) | Grower_Billy_Deaths[HHD_Gender, SR_activity_responsibility](t - dt) + (Growers_billy_Dying[HHD_Gender, SR_activity_responsibility]) * dt | INIT Grower_Billy_Deaths[HHD_Gender, SR_activity_responsibility] = 0 | Goat |  | NON-NEGATIVE |
| Grower_Ewe_6_to_12_months[HHD_Gender, SR_activity_responsibility](t) | Grower_Ewe_6_to_12_months[HHD_Gender, SR_activity_responsibility](t - dt) + (Grower_Ewe_6_to_12_months_vaccinated_recovery_rate[HHD_Gender, SR_activity_responsibility] + Growing_Ewe_natural_recovered[HHD_Gender, SR_activity_responsibility] + Growing_Ewe_vaccinated_identified[HHD_Gender, SR_activity_responsibility] - Becoming_Adult_Ewe_Vaccinated_identified[HHD_Gender, SR_activity_responsibility] - Becoming_Adult_Ewe_vaccinated_unidentified[HHD_Gender, SR_activity_responsibility]) * dt | INIT Grower_Ewe_6_to_12_months[HHD_Gender, SR_activity_responsibility] = 0 | Sheep |  | NON-NEGATIVE |
| Grower_Ewe_6_to_12_months_infected[HHD_Gender, SR_activity_responsibility](t) | Grower_Ewe_6_to_12_months_infected[HHD_Gender, SR_activity_responsibility](t - dt) + (Grower_Ewe_6_to_12_months_infection_rate[HHD_Gender, SR_activity_responsibility] - Grower_Ewe_6_to_12_months_natural_recovery_rate[HHD_Gender, SR_activity_responsibility] - Grower_Ewes_dying[HHD_Gender, SR_activity_responsibility]) * dt | INIT Grower_Ewe_6_to_12_months_infected[HHD_Gender, SR_activity_responsibility] = 0 | Sheep |  | NON-NEGATIVE |
| Grower_Ewe_6_to_12_months_natural_recovered[HHD_Gender, SR_activity_responsibility](t) | Grower_Ewe_6_to_12_months_natural_recovered[HHD_Gender, SR_activity_responsibility](t - dt) + (Grower_Ewe_6_to_12_months_natural_recovery_rate[HHD_Gender, SR_activity_responsibility] - Ewe_given_out[HHD_Gender, SR_activity_responsibility] - Becoming_Adult_Ewe_natural_recovered[HHD_Gender, SR_activity_responsibility]) * dt | INIT Grower_Ewe_6_to_12_months_natural_recovered[HHD_Gender, SR_activity_responsibility] = 0 | Sheep |  | NON-NEGATIVE |
| Grower_Ewe_6_to_12_months_susceptible[HHD_Gender, SR_activity_responsibility](t) | Grower_Ewe_6_to_12_months_susceptible[HHD_Gender, SR_activity_responsibility](t - dt) + ( - Grower_Ewe_6_to_12_months_vaccinating[HHD_Gender, SR_activity_responsibility] - Grower_Ewe_6_to_12_months_infection_rate[HHD_Gender, SR_activity_responsibility] - Grower_Ewe_6_to_12_months_stolen[HHD_Gender, SR_activity_responsibility]) * dt | INIT Grower_Ewe_6_to_12_months_susceptible[HHD_Gender, SR_activity_responsibility] = TRIANGULAR(0, 1, 5) {HH survey data} | Sheep |  | NON-NEGATIVE |
| Grower_Ewe_6_to_12_months_vaccinated[HHD_Gender, SR_activity_responsibility](t) | Grower_Ewe_6_to_12_months_vaccinated[HHD_Gender, SR_activity_responsibility](t - dt) + (Grower_Ewe_6_to_12_months_vaccinating[HHD_Gender, SR_activity_responsibility] - Grower_Ewe_6_to_12_months_vaccinated_recovery_rate[HHD_Gender, SR_activity_responsibility]) * dt | INIT Grower_Ewe_6_to_12_months_vaccinated[HHD_Gender, SR_activity_responsibility] = 0 | Sheep |  | NON-NEGATIVE |
| Grower_Ewes_6_to_12_months_Death[HHD_Gender, SR_activity_responsibility](t) | Grower_Ewes_6_to_12_months_Death[HHD_Gender, SR_activity_responsibility](t - dt) + (Grower_Ewes_dying[HHD_Gender, SR_activity_responsibility]) * dt | INIT Grower_Ewes_6_to_12_months_Death[HHD_Gender, SR_activity_responsibility] = 0 | Sheep |  | NON-NEGATIVE |
| Grower_nanny_6_to_12_months_infected[HHD_Gender, SR_activity_responsibility](t) | Grower_nanny_6_to_12_months_infected[HHD_Gender, SR_activity_responsibility](t - dt) + (Grower_nanny_6_to_12_months_infection_rate[HHD_Gender, SR_activity_responsibility] - Grower_nanny_Dying[HHD_Gender, SR_activity_responsibility] - Grower_nanny_6_to_12_months_recovery_rate[HHD_Gender, SR_activity_responsibility]) * dt | INIT Grower_nanny_6_to_12_months_infected[HHD_Gender, SR_activity_responsibility] = 1 | Goat |  | NON-NEGATIVE |
| Grower_nanny_6_to_12_months_recovered[HHD_Gender, SR_activity_responsibility](t) | Grower_nanny_6_to_12_months_recovered[HHD_Gender, SR_activity_responsibility](t - dt) + (Grower_nanny_6_to_12_months_recovery_rate[HHD_Gender, SR_activity_responsibility] - Nanny_given_out[HHD_Gender, SR_activity_responsibility] - Adulting_nanny_becoming_vulnerable[HHD_Gender, SR_activity_responsibility]) * dt | INIT Grower_nanny_6_to_12_months_recovered[HHD_Gender, SR_activity_responsibility] = 0 | Goat |  | NON-NEGATIVE |
| Grower_nanny_6_to_12_months_susceptible[HHD_Gender, SR_activity_responsibility](t) | Grower_nanny_6_to_12_months_susceptible[HHD_Gender, SR_activity_responsibility](t - dt) + ( - Grower_nanny_6_to_12_months_vaccinating[HHD_Gender, SR_activity_responsibility] - Grower_nanny_6_to_12_months_infection_rate[HHD_Gender, SR_activity_responsibility] - Grower_nanny_stolen[HHD_Gender, SR_activity_responsibility]) * dt | INIT Grower_nanny_6_to_12_months_susceptible[HHD_Gender, SR_activity_responsibility] = TRIANGULAR(1, 2, 5){HH survey data} | Goat |  | NON-NEGATIVE |
| Grower_nanny_6_to_12_months_vaccinated[HHD_Gender, SR_activity_responsibility](t) | Grower_nanny_6_to_12_months_vaccinated[HHD_Gender, SR_activity_responsibility](t - dt) + (Grower_nanny_6_to_12_months_vaccinating[HHD_Gender, SR_activity_responsibility] - Grower_nanny_6_to_12_months_vaccinated_recovery_rate[HHD_Gender, SR_activity_responsibility]) * dt | INIT Grower_nanny_6_to_12_months_vaccinated[HHD_Gender, SR_activity_responsibility] = 0 | Goat |  | NON-NEGATIVE |
| Grower_nanny_6_to_12_months_vaccinated_recovered[HHD_Gender, SR_activity_responsibility](t) | Grower_nanny_6_to_12_months_vaccinated_recovered[HHD_Gender, SR_activity_responsibility](t - dt) + (Grower_nanny_6_to_12_months_vaccinated_recovery_rate[HHD_Gender, SR_activity_responsibility] + Growing_nanny_becoming_vulnerable[HHD_Gender, SR_activity_responsibility] + Growing_nanny_vaccinated_identified[HHD_Gender, SR_activity_responsibility] - "Becoming_adult_nanny_>_12_months_vaccinated"[HHD_Gender, SR_activity_responsibility] - "Becoming_adult_nanny_>_12_months_vaccinated_unidentified"[HHD_Gender, SR_activity_responsibility]) * dt | INIT Grower_nanny_6_to_12_months_vaccinated_recovered[HHD_Gender, SR_activity_responsibility] = 0 | Goat |  | NON-NEGATIVE |
| Grower_Nanny_Death[HHD_Gender, SR_activity_responsibility](t) | Grower_Nanny_Death[HHD_Gender, SR_activity_responsibility](t - dt) + (Grower_nanny_Dying[HHD_Gender, SR_activity_responsibility]) * dt | INIT Grower_Nanny_Death[HHD_Gender, SR_activity_responsibility] = 0 | Goat |  | NON-NEGATIVE |
| Grower_Rams_6_to_12_months[HHD_Gender, SR_activity_responsibility](t) | Grower_Rams_6_to_12_months[HHD_Gender, SR_activity_responsibility](t - dt) + (Growing_Ram_natural_recovery[HHD_Gender, SR_activity_responsibility] + Grower_Rams_6_to_12_months_vaccinated_recovery_rate[HHD_Gender, SR_activity_responsibility] + Growing_Ram_vaccinated_identified[HHD_Gender, SR_activity_responsibility] - Becoming_Adult_Ram_vaccinated_identified[HHD_Gender, SR_activity_responsibility] - Grower_Ram_given_out[HHD_Gender, SR_activity_responsibility] - Becoming_Adult_Ram_vaccinated_but_unidentified[HHD_Gender, SR_activity_responsibility]) * dt | INIT Grower_Rams_6_to_12_months[HHD_Gender, SR_activity_responsibility] = 0 | Sheep |  | NON-NEGATIVE |
| Grower_Rams_6_to_12_months_Death[HHD_Gender, SR_activity_responsibility](t) | Grower_Rams_6_to_12_months_Death[HHD_Gender, SR_activity_responsibility](t - dt) + (Growers_Ram_Deaths[HHD_Gender, SR_activity_responsibility]) * dt | INIT Grower_Rams_6_to_12_months_Death[HHD_Gender, SR_activity_responsibility] = 0 | Sheep |  | NON-NEGATIVE |
| Grower_Rams_6_to_12_months_infected[HHD_Gender, SR_activity_responsibility](t) | Grower_Rams_6_to_12_months_infected[HHD_Gender, SR_activity_responsibility](t - dt) + (Grower_Rams_6_to_12_months_infection_rate[HHD_Gender, SR_activity_responsibility] - Growers_Ram_Deaths[HHD_Gender, SR_activity_responsibility] - Grower_Rams_6_to_12_months_natural_recovery_rate[HHD_Gender, SR_activity_responsibility]) * dt | INIT Grower_Rams_6_to_12_months_infected[HHD_Gender, SR_activity_responsibility] = 0 | Sheep |  | NON-NEGATIVE |
| Grower_Rams_6_to_12_months_natural_recovered[HHD_Gender, SR_activity_responsibility](t) | Grower_Rams_6_to_12_months_natural_recovered[HHD_Gender, SR_activity_responsibility](t - dt) + (Grower_Rams_6_to_12_months_natural_recovery_rate[HHD_Gender, SR_activity_responsibility] - Becoming_Adult_Ram_natural_recovery[HHD_Gender, SR_activity_responsibility]) * dt | INIT Grower_Rams_6_to_12_months_natural_recovered[HHD_Gender, SR_activity_responsibility] = 0 | Sheep |  | NON-NEGATIVE |
| Grower_Rams_6_to_12_months_Susceptible[HHD_Gender, SR_activity_responsibility](t) | Grower_Rams_6_to_12_months_Susceptible[HHD_Gender, SR_activity_responsibility](t - dt) + ( - Grower_Rams_6_to_12_months_infection_rate[HHD_Gender, SR_activity_responsibility] - Grower_Rams_6_to_12_months_vaccinating[HHD_Gender, SR_activity_responsibility] - Grower_3_to_6_months_stolen[HHD_Gender, SR_activity_responsibility]) * dt | INIT Grower_Rams_6_to_12_months_Susceptible[HHD_Gender, SR_activity_responsibility] = TRIANGULAR(0, 2, 15) {HH survey data} | Sheep |  | NON-NEGATIVE |
| Grower_Rams_6_to_12_months_vaccinated[HHD_Gender, SR_activity_responsibility](t) | Grower_Rams_6_to_12_months_vaccinated[HHD_Gender, SR_activity_responsibility](t - dt) + (Grower_Rams_6_to_12_months_vaccinating[HHD_Gender, SR_activity_responsibility] - Grower_Rams_6_to_12_months_vaccinated_recovery_rate[HHD_Gender, SR_activity_responsibility]) * dt | INIT Grower_Rams_6_to_12_months_vaccinated[HHD_Gender, SR_activity_responsibility] = 0 | Sheep |  | NON-NEGATIVE |
| "Kid_>3_months"[HHD_Gender, SR_activity_responsibility](t) | "Kid_>3_months"[HHD_Gender, SR_activity_responsibility](t - dt) + (Kidding[HHD_Gender, SR_activity_responsibility] - Weaning_billy_goat[HHD_Gender, SR_activity_responsibility] - Weaning_nanny_goat[HHD_Gender, SR_activity_responsibility] - "Kid_>3_months_Dying"[HHD_Gender, SR_activity_responsibility]) * dt | INIT "Kid_>3_months"[HHD_Gender, SR_activity_responsibility] = TRIANGULAR(1, 3, 30) {HH survey data} | Goat |  | NON-NEGATIVE |
| "Kid_>3_months_Death"[HHD_Gender, SR_activity_responsibility](t) | "Kid_>3_months_Death"[HHD_Gender, SR_activity_responsibility](t - dt) + ("Kid_>3_months_Dying"[HHD_Gender, SR_activity_responsibility]) * dt | INIT "Kid_>3_months_Death"[HHD_Gender, SR_activity_responsibility] = 0 | Goat |  | NON-NEGATIVE |
| Kid_Still_Birth_Death[HHD_Gender, SR_activity_responsibility](t) | Kid_Still_Birth_Death[HHD_Gender, SR_activity_responsibility](t - dt) + (Kid_Still_born_rate[HHD_Gender, SR_activity_responsibility]) * dt | INIT Kid_Still_Birth_Death[HHD_Gender, SR_activity_responsibility] = 0 | Goat |  | NON-NEGATIVE |
| "Lamb_>3_months_Death"[HHD_Gender, SR_activity_responsibility](t) | "Lamb_>3_months_Death"[HHD_Gender, SR_activity_responsibility](t - dt) + ("Lamb_>3_months_Dying"[HHD_Gender, SR_activity_responsibility]) * dt | INIT "Lamb_>3_months_Death"[HHD_Gender, SR_activity_responsibility] = 0 | Sheep |  | NON-NEGATIVE |
| "Lambs_>3_months"[HHD_Gender, SR_activity_responsibility](t) | "Lambs_>3_months"[HHD_Gender, SR_activity_responsibility](t - dt) + (Lambing[HHD_Gender, SR_activity_responsibility] - "Lamb_>3_months_Dying"[HHD_Gender, SR_activity_responsibility] - Weaning_Ewe[HHD_Gender, SR_activity_responsibility] - Weaning_Ram[HHD_Gender, SR_activity_responsibility]) * dt | INIT "Lambs_>3_months"[HHD_Gender, SR_activity_responsibility] = TRIANGULAR(1, 3, 12) {HH survey data} | Sheep |  | NON-NEGATIVE |
| Sheep_Gestation_Delay[HHD_Gender, SR_activity_responsibility](t) | Sheep_Gestation_Delay[HHD_Gender, SR_activity_responsibility](t - dt) + (Lamb_Breeding_rate[HHD_Gender, SR_activity_responsibility] - Ram_Still_born_rate[HHD_Gender, SR_activity_responsibility] - Lambing[HHD_Gender, SR_activity_responsibility]) * dt | INIT Sheep_Gestation_Delay[HHD_Gender, SR_activity_responsibility] = 0 | Sheep |  | NON-NEGATIVE |
| "Sold_Adult_Ewe_>12_months"[HHD_Gender, SR_activity_responsibility](t) | "Sold_Adult_Ewe_>12_months"[HHD_Gender, SR_activity_responsibility](t - dt) + ("Adult_Ewe_>12_months_selling"[HHD_Gender, SR_activity_responsibility]) * dt | INIT "Sold_Adult_Ewe_>12_months"[HHD_Gender, SR_activity_responsibility] = 0 | Sheep |  | NON-NEGATIVE |
| Sold_Adult_ram[HHD_Gender, SR_activity_responsibility](t) | Sold_Adult_ram[HHD_Gender, SR_activity_responsibility](t - dt) + (Selling_Adult_Ram[HHD_Gender, SR_activity_responsibility]) * dt | INIT Sold_Adult_ram[HHD_Gender, SR_activity_responsibility] = 0 | Sheep |  | NON-NEGATIVE |
| Sold_Cull_Adult_Ewe[HHD_Gender, SR_activity_responsibility](t) | Sold_Cull_Adult_Ewe[HHD_Gender, SR_activity_responsibility](t - dt) + (Selling_Cull_Adult_Ewe[HHD_Gender, SR_activity_responsibility]) * dt | INIT Sold_Cull_Adult_Ewe[HHD_Gender, SR_activity_responsibility] = 0 | Sheep |  | NON-NEGATIVE |
| Sold_Cull_Adult_nanny[HHD_Gender, SR_activity_responsibility](t) | Sold_Cull_Adult_nanny[HHD_Gender, SR_activity_responsibility](t - dt) + (Cull_selling_adult_nanny[HHD_Gender, SR_activity_responsibility]) * dt | INIT Sold_Cull_Adult_nanny[HHD_Gender, SR_activity_responsibility] = 0 | Goat |  | NON-NEGATIVE |
| Sold_Reserved_Adult_Ram[HHD_Gender, SR_activity_responsibility](t) | Sold_Reserved_Adult_Ram[HHD_Gender, SR_activity_responsibility](t - dt) + (Selling_Reserved_Adult_Ram[HHD_Gender, SR_activity_responsibility]) * dt | INIT Sold_Reserved_Adult_Ram[HHD_Gender, SR_activity_responsibility] = 0 | Sheep |  | NON-NEGATIVE |
| Sold_Young_Ewe[HHD_Gender, SR_activity_responsibility](t) | Sold_Young_Ewe[HHD_Gender, SR_activity_responsibility](t - dt) + (Young_Ewe_selling[HHD_Gender, SR_activity_responsibility]) * dt | INIT Sold_Young_Ewe[HHD_Gender, SR_activity_responsibility] = 0 | Sheep |  | NON-NEGATIVE |
| Sold_Young_Ram_3_to_6_months_natural_recovered[HHD_Gender, SR_activity_responsibility](t) | Sold_Young_Ram_3_to_6_months_natural_recovered[HHD_Gender, SR_activity_responsibility](t - dt) + (Young_Ram_3_to_6_months_natural_recovered_sold[HHD_Gender, SR_activity_responsibility]) * dt | INIT Sold_Young_Ram_3_to_6_months_natural_recovered[HHD_Gender, SR_activity_responsibility] = 0 | Sheep |  | NON-NEGATIVE |
| Sold_Young_Ram_3_to_6_recovered[HHD_Gender, SR_activity_responsibility](t) | Sold_Young_Ram_3_to_6_recovered[HHD_Gender, SR_activity_responsibility](t - dt) + (Young_Ram_3_to_6_months_vaccinated_recovered_selling[HHD_Gender, SR_activity_responsibility]) * dt | INIT Sold_Young_Ram_3_to_6_recovered[HHD_Gender, SR_activity_responsibility] = 0 | Sheep |  | NON-NEGATIVE |
| Still_Birth_Ram_Death[HHD_Gender, SR_activity_responsibility](t) | Still_Birth_Ram_Death[HHD_Gender, SR_activity_responsibility](t - dt) + (Ram_Still_born_rate[HHD_Gender, SR_activity_responsibility]) * dt | INIT Still_Birth_Ram_Death[HHD_Gender, SR_activity_responsibility] = 0 | Sheep |  | NON-NEGATIVE |
| Young_billy_3_to_6_months_infected[HHD_Gender, SR_activity_responsibility](t) | Young_billy_3_to_6_months_infected[HHD_Gender, SR_activity_responsibility](t - dt) + (Young_billy_3_to_6_months_infection_rate[HHD_Gender, SR_activity_responsibility] - Young_billy_Dying[HHD_Gender, SR_activity_responsibility] - Young_billy_3_to_6_months_recovery_rate[HHD_Gender, SR_activity_responsibility]) * dt | INIT Young_billy_3_to_6_months_infected[HHD_Gender, SR_activity_responsibility] = 0 | Goat |  | NON-NEGATIVE |
| Young_billy_3_to_6_months_recovered[HHD_Gender, SR_activity_responsibility](t) | Young_billy_3_to_6_months_recovered[HHD_Gender, SR_activity_responsibility](t - dt) + (Young_billy_3_to_6_months_recovery_rate[HHD_Gender, SR_activity_responsibility] - Young_billy_selling[HHD_Gender, SR_activity_responsibility] - Growing_billy_natural_recovered[HHD_Gender, SR_activity_responsibility]) * dt | INIT Young_billy_3_to_6_months_recovered[HHD_Gender, SR_activity_responsibility] = 0 | Goat |  | NON-NEGATIVE |
| Young_billy_3_to_6_months_susceptible[HHD_Gender, SR_activity_responsibility](t) | Young_billy_3_to_6_months_susceptible[HHD_Gender, SR_activity_responsibility](t - dt) + (Weaning_billy_goat[HHD_Gender, SR_activity_responsibility] - Young_billy_3_to_6_months_infection_rate[HHD_Gender, SR_activity_responsibility] - Young_billy_3_to_6_months_vaccinating[HHD_Gender, SR_activity_responsibility] - Young_kid_stolen[HHD_Gender, SR_activity_responsibility]) * dt | INIT Young_billy_3_to_6_months_susceptible[HHD_Gender, SR_activity_responsibility] = TRIANGULAR(0, 2, 25) {HH survey data} | Goat |  | NON-NEGATIVE |
| Young_billy_3_to_6_months_vaccinated[HHD_Gender, SR_activity_responsibility](t) | Young_billy_3_to_6_months_vaccinated[HHD_Gender, SR_activity_responsibility](t - dt) + (Young_billy_3_to_6_months_vaccinating[HHD_Gender, SR_activity_responsibility] - Young_billy_3_to_6_months_vaccinated_recovery_rate[HHD_Gender, SR_activity_responsibility]) * dt | INIT Young_billy_3_to_6_months_vaccinated[HHD_Gender, SR_activity_responsibility] = 0 | Goat |  | NON-NEGATIVE |
| Young_billy_3_to_6_months_vaccinated_recovered[HHD_Gender, SR_activity_responsibility](t) | Young_billy_3_to_6_months_vaccinated_recovered[HHD_Gender, SR_activity_responsibility](t - dt) + (Young_billy_3_to_6_months_vaccinated_recovery_rate[HHD_Gender, SR_activity_responsibility] - Growing_billy_vaccinated_identified[HHD_Gender, SR_activity_responsibility] - Growing_billy_vaccinated_unidentified[HHD_Gender, SR_activity_responsibility]) * dt | INIT Young_billy_3_to_6_months_vaccinated_recovered[HHD_Gender, SR_activity_responsibility] = 0 | Goat |  | NON-NEGATIVE |
| Young_Billy_Deaths[HHD_Gender, SR_activity_responsibility](t) | Young_Billy_Deaths[HHD_Gender, SR_activity_responsibility](t - dt) + (Young_billy_Dying[HHD_Gender, SR_activity_responsibility]) * dt | INIT Young_Billy_Deaths[HHD_Gender, SR_activity_responsibility] = 0 | Goat |  | NON-NEGATIVE |
| Young_billy_Sold[HHD_Gender, SR_activity_responsibility](t) | Young_billy_Sold[HHD_Gender, SR_activity_responsibility](t - dt) + (Young_billy_selling[HHD_Gender, SR_activity_responsibility]) * dt | INIT Young_billy_Sold[HHD_Gender, SR_activity_responsibility] = 0 | Goat |  | NON-NEGATIVE |
| Young_Ewe_3_to_6_months_Death[HHD_Gender, SR_activity_responsibility](t) | Young_Ewe_3_to_6_months_Death[HHD_Gender, SR_activity_responsibility](t - dt) + (Young_Ewe_3_to_6_months_dying[HHD_Gender, SR_activity_responsibility]) * dt | INIT Young_Ewe_3_to_6_months_Death[HHD_Gender, SR_activity_responsibility] = 0 | Sheep |  | NON-NEGATIVE |
| Young_Ewe_3_to_6_months_infected[HHD_Gender, SR_activity_responsibility](t) | Young_Ewe_3_to_6_months_infected[HHD_Gender, SR_activity_responsibility](t - dt) + (Young_Ewe_3_to_6_months_infection_rate[HHD_Gender, SR_activity_responsibility] - Young_Ewe_3_to_6_months_dying[HHD_Gender, SR_activity_responsibility] - Young_Ewe_3_to_6_months_natural_recovery_rate[HHD_Gender, SR_activity_responsibility]) * dt | INIT Young_Ewe_3_to_6_months_infected[HHD_Gender, SR_activity_responsibility] = 0 | Sheep |  | NON-NEGATIVE |
| Young_Ewe_3_to_6_months_natural_recovered[HHD_Gender, SR_activity_responsibility](t) | Young_Ewe_3_to_6_months_natural_recovered[HHD_Gender, SR_activity_responsibility](t - dt) + (Young_Ewe_3_to_6_months_natural_recovery_rate[HHD_Gender, SR_activity_responsibility] - Growing_Ewe_natural_recovered[HHD_Gender, SR_activity_responsibility] - Young_Ewe_selling[HHD_Gender, SR_activity_responsibility]) * dt | INIT Young_Ewe_3_to_6_months_natural_recovered[HHD_Gender, SR_activity_responsibility] = 0 | Sheep |  | NON-NEGATIVE |
| Young_Ewe_3_to_6_months_susceptible[HHD_Gender, SR_activity_responsibility](t) | Young_Ewe_3_to_6_months_susceptible[HHD_Gender, SR_activity_responsibility](t - dt) + (Weaning_Ewe[HHD_Gender, SR_activity_responsibility] + Young_sheep_received_as_gift[HHD_Gender, SR_activity_responsibility] - Young_Ewe_3_to_6_months_infection_rate[HHD_Gender, SR_activity_responsibility] - Young_Ewe_3_to_6_months_vaccinating[HHD_Gender, SR_activity_responsibility] - Young_Ewe_3_to_6_months_stolen[HHD_Gender, SR_activity_responsibility]) * dt | INIT Young_Ewe_3_to_6_months_susceptible[HHD_Gender, SR_activity_responsibility] = TRIANGULAR(0, 1, 20) {HH survey data} | Sheep |  | NON-NEGATIVE |
| Young_Ewe_3_to_6_months_vaccinated[HHD_Gender, SR_activity_responsibility](t) | Young_Ewe_3_to_6_months_vaccinated[HHD_Gender, SR_activity_responsibility](t - dt) + (Young_Ewe_3_to_6_months_vaccinating[HHD_Gender, SR_activity_responsibility] - Young_Ewe_3_to_6_months_vaccinated_recovery_rate[HHD_Gender, SR_activity_responsibility]) * dt | INIT Young_Ewe_3_to_6_months_vaccinated[HHD_Gender, SR_activity_responsibility] = 0 | Sheep |  | NON-NEGATIVE |
| Young_Ewe_3_to_6_months_vaccinated_recovered[HHD_Gender, SR_activity_responsibility](t) | Young_Ewe_3_to_6_months_vaccinated_recovered[HHD_Gender, SR_activity_responsibility](t - dt) + (Young_Ewe_3_to_6_months_vaccinated_recovery_rate[HHD_Gender, SR_activity_responsibility] - Growing_Ewe_vaccinated_identified[HHD_Gender, SR_activity_responsibility] - Growing_Ewe_vaccinated_but_unidentified[HHD_Gender, SR_activity_responsibility]) * dt | INIT Young_Ewe_3_to_6_months_vaccinated_recovered[HHD_Gender, SR_activity_responsibility] = 0 | Sheep |  | NON-NEGATIVE |
| Young_nanny_3_to_6_months_infected[HHD_Gender, SR_activity_responsibility](t) | Young_nanny_3_to_6_months_infected[HHD_Gender, SR_activity_responsibility](t - dt) + (Young_nanny_3_to_6_months_infection_rate[HHD_Gender, SR_activity_responsibility] - Young_Nanny_Dying[HHD_Gender, SR_activity_responsibility] - Young_nanny_3_to_6_months_recovery_rate[HHD_Gender, SR_activity_responsibility]) * dt | INIT Young_nanny_3_to_6_months_infected[HHD_Gender, SR_activity_responsibility] = 0 | Goat |  | NON-NEGATIVE |
| Young_nanny_3_to_6_months_recovered[HHD_Gender, SR_activity_responsibility](t) | Young_nanny_3_to_6_months_recovered[HHD_Gender, SR_activity_responsibility](t - dt) + (Young_nanny_3_to_6_months_recovery_rate[HHD_Gender, SR_activity_responsibility] - Young_nanny_Selling[HHD_Gender, SR_activity_responsibility] - Growing_nanny_becoming_vulnerable[HHD_Gender, SR_activity_responsibility]) * dt | INIT Young_nanny_3_to_6_months_recovered[HHD_Gender, SR_activity_responsibility] = 0 | Goat |  | NON-NEGATIVE |
| Young_nanny_3_to_6_months_susceptible[HHD_Gender, SR_activity_responsibility](t) | Young_nanny_3_to_6_months_susceptible[HHD_Gender, SR_activity_responsibility](t - dt) + (Weaning_nanny_goat[HHD_Gender, SR_activity_responsibility] - Young_nanny_3_to_6_months_infection_rate[HHD_Gender, SR_activity_responsibility] - Young_nanny_3_to_6_months_vaccinating[HHD_Gender, SR_activity_responsibility] - Young_nanny_stolen[HHD_Gender, SR_activity_responsibility]) * dt | INIT Young_nanny_3_to_6_months_susceptible[HHD_Gender, SR_activity_responsibility] = TRIANGULAR(0, 3, 30) {HH survey data} | Goat |  | NON-NEGATIVE |
| Young_nanny_3_to_6_months_vaccinated[HHD_Gender, SR_activity_responsibility](t) | Young_nanny_3_to_6_months_vaccinated[HHD_Gender, SR_activity_responsibility](t - dt) + (Young_nanny_3_to_6_months_vaccinating[HHD_Gender, SR_activity_responsibility] - Young_nanny_3_to_6_months_vaccinated_recovery_rate[HHD_Gender, SR_activity_responsibility]) * dt | INIT Young_nanny_3_to_6_months_vaccinated[HHD_Gender, SR_activity_responsibility] = 0 | Goat |  | NON-NEGATIVE |
| Young_nanny_3_to_6_months_vaccinated_recovered[HHD_Gender, SR_activity_responsibility](t) | Young_nanny_3_to_6_months_vaccinated_recovered[HHD_Gender, SR_activity_responsibility](t - dt) + (Young_nanny_3_to_6_months_vaccinated_recovery_rate[HHD_Gender, SR_activity_responsibility] - Growing_nanny_vaccinated_identified[HHD_Gender, SR_activity_responsibility] - Growing_nanny_vaccinated_unidentified[HHD_Gender, SR_activity_responsibility]) * dt | INIT Young_nanny_3_to_6_months_vaccinated_recovered[HHD_Gender, SR_activity_responsibility] = 0 | Goat |  | NON-NEGATIVE |
| Young_Nanny_Death[HHD_Gender, SR_activity_responsibility](t) | Young_Nanny_Death[HHD_Gender, SR_activity_responsibility](t - dt) + (Young_Nanny_Dying[HHD_Gender, SR_activity_responsibility]) * dt | INIT Young_Nanny_Death[HHD_Gender, SR_activity_responsibility] = 0 | Goat |  | NON-NEGATIVE |
| Young_nanny_Sold[HHD_Gender, SR_activity_responsibility](t) | Young_nanny_Sold[HHD_Gender, SR_activity_responsibility](t - dt) + (Young_nanny_Selling[HHD_Gender, SR_activity_responsibility]) * dt | INIT Young_nanny_Sold[HHD_Gender, SR_activity_responsibility] = 0 | Goat |  | NON-NEGATIVE |
| Young_Ram_3_to_6_months_Death[HHD_Gender, SR_activity_responsibility](t) | Young_Ram_3_to_6_months_Death[HHD_Gender, SR_activity_responsibility](t - dt) + (Young_Ram_Dying[HHD_Gender, SR_activity_responsibility]) * dt | INIT Young_Ram_3_to_6_months_Death[HHD_Gender, SR_activity_responsibility] = 0 | Sheep |  | NON-NEGATIVE |
| Young_Ram_3_to_6_months_infected[HHD_Gender, SR_activity_responsibility](t) | Young_Ram_3_to_6_months_infected[HHD_Gender, SR_activity_responsibility](t - dt) + (Young_Ram_3_to_6_months_infection_rate[HHD_Gender, SR_activity_responsibility] - Young_Ram_3_to_6_months_natural_recovery_rate[HHD_Gender, SR_activity_responsibility] - Young_Ram_Dying[HHD_Gender, SR_activity_responsibility]) * dt | INIT Young_Ram_3_to_6_months_infected[HHD_Gender, SR_activity_responsibility] = 1 | Sheep |  | NON-NEGATIVE |
| Young_Ram_3_to_6_months_natural_recovered[HHD_Gender, SR_activity_responsibility](t) | Young_Ram_3_to_6_months_natural_recovered[HHD_Gender, SR_activity_responsibility](t - dt) + (Young_Ram_3_to_6_months_natural_recovery_rate[HHD_Gender, SR_activity_responsibility] - Young_Ram_3_to_6_months_natural_recovered_sold[HHD_Gender, SR_activity_responsibility] - Growing_Ram_natural_recovery[HHD_Gender, SR_activity_responsibility]) * dt | INIT Young_Ram_3_to_6_months_natural_recovered[HHD_Gender, SR_activity_responsibility] = 0 | Sheep |  | NON-NEGATIVE |
| Young_Ram_3_to_6_months_susceptible[HHD_Gender, SR_activity_responsibility](t) | Young_Ram_3_to_6_months_susceptible[HHD_Gender, SR_activity_responsibility](t - dt) + (Weaning_Ram[HHD_Gender, SR_activity_responsibility] - Young_Ram_3_to_6_months_vaccinating[HHD_Gender, SR_activity_responsibility] - Young_ram_3_to_6_months_stolen[HHD_Gender, SR_activity_responsibility] - Young_Ram_3_to_6_months_infection_rate[HHD_Gender, SR_activity_responsibility]) * dt | INIT Young_Ram_3_to_6_months_susceptible[HHD_Gender, SR_activity_responsibility] = TRIANGULAR(0, 2, 11){HH survey data} | Sheep |  | NON-NEGATIVE |
| Young_Ram_3_to_6_months_vaccinated[HHD_Gender, SR_activity_responsibility](t) | Young_Ram_3_to_6_months_vaccinated[HHD_Gender, SR_activity_responsibility](t - dt) + (Young_Ram_3_to_6_months_vaccinating[HHD_Gender, SR_activity_responsibility] - Young_Ram_3_to_6_months_vaccinated_recovery_rate[HHD_Gender, SR_activity_responsibility]) * dt | INIT Young_Ram_3_to_6_months_vaccinated[HHD_Gender, SR_activity_responsibility] = 0 | Sheep |  | NON-NEGATIVE |
| Young_Ram_3_to_6_months_vaccinated_recovered[HHD_Gender, SR_activity_responsibility](t) | Young_Ram_3_to_6_months_vaccinated_recovered[HHD_Gender, SR_activity_responsibility](t - dt) + (Young_Ram_3_to_6_months_vaccinated_recovery_rate[HHD_Gender, SR_activity_responsibility] - Young_Ram_3_to_6_months_vaccinated_recovered_selling[HHD_Gender, SR_activity_responsibility] - Growing_Ram_vaccinated_identified[HHD_Gender, SR_activity_responsibility] - Growing_Ram_vaccinated_but_unidentified[HHD_Gender, SR_activity_responsibility]) * dt | INIT Young_Ram_3_to_6_months_vaccinated_recovered[HHD_Gender, SR_activity_responsibility] = 0 | Sheep |  | NON-NEGATIVE |
| "Adult_billy_>_12_months_infection_rate"[HHD_Gender, SR_activity_responsibility] | (Contact_rate*"Seroprevalence_Adult_goat_>12_months"*"Adult_billy_>_12_months_susceptible")*("Adult_billy_>12_months_infected"/Total_goat) | OUTFLOW PRIORITY: 1 | Goat/Weeks |  | UNIFLOW |
| "Adult_billy_>_12_months_recovery_rate"[HHD_Gender, SR_activity_responsibility] | "Adult_billy_>12_months_infected"/Average_PPR_illness_duration | OUTFLOW PRIORITY: 2 | Goat/Weeks |  | UNIFLOW |
| "Adult_billy_>12_months_vaccinating"[HHD_Gender, SR_activity_responsibility] | (Disease_Control_Module."Adult_billy_>12_months_vaccination_rate"*Disease_Control_Module."Sero-conversion_rate")+Becoming_adult_vaccinated_unidentified | OUTFLOW PRIORITY: 2 | Goat/Weeks |  | UNIFLOW |
| Adult_billy_Dying[HHD_Gender, SR_activity_responsibility] | "Adult_billy_>12_months_infected"*"Mortality_rate_>young_SR" | OUTFLOW PRIORITY: 1 | Goat/Weeks |  | UNIFLOW |
| Adult_billy_stolen[HHD_Gender, SR_activity_responsibility] | Number_stolen_goat | OUTFLOW PRIORITY: 3 | Goat/Weeks |  | UNIFLOW |
| Adult_billy_vaccinated_becoming_recovered[HHD_Gender, SR_activity_responsibility] | "Adult_billy_>12_months_vaccinated"/Average_PPR_illness_duration |  | Goat/Weeks |  | UNIFLOW |
| "Adult_Ewe_>_12_months_recovery_rate"[HHD_Gender, SR_activity_responsibility] | "Adult_Ewe_>12_months_infected"/Average_PPR_illness_duration | OUTFLOW PRIORITY: 1 | Sheep per week |  | UNIFLOW |
| "Adult_Ewe_>12_months_infection_rate"[HHD_Gender, SR_activity_responsibility] | (Contact_rate*"Seroprevalence_Adult_sheep_>12_months"*"Adult_Ewe_>_12_months_susceptible")*("Adult_Ewe_>12_months_infected"/Total_sheep) | OUTFLOW PRIORITY: 1 | Sheep per week |  | UNIFLOW |
| "Adult_Ewe_>12_months_selling"[HHD_Gender, SR_activity_responsibility] | "Adult_Ewe_>12_months"/Time_to_sell_adult_ewe | OUTFLOW PRIORITY: 2 | Sheep per week |  | UNIFLOW |
| Adult_Ewe_stolen[HHD_Gender, SR_activity_responsibility] | Number_stolen | OUTFLOW PRIORITY: 3 | Sheep per week |  | UNIFLOW |
| "Adult_Ewes_>12_months_vaccinating"[HHD_Gender, SR_activity_responsibility] | (Disease_Control_Module."Adult_Ewe_>_12_months_vaccination_rate"*Disease_Control_Module."Sero-conversion_rate")+Becoming_Adult_Ewe_vaccinated_unidentified | OUTFLOW PRIORITY: 2 | Sheep per week |  | UNIFLOW |
| Adult_ewes_dying[HHD_Gender, SR_activity_responsibility] | "Adult_Ewe_>12_months_infected"*"Mortality_rate_>young_SR" | OUTFLOW PRIORITY: 2 | Sheep per week |  | UNIFLOW |
| "Adult_nanny_>_12_months_dying"[HHD_Gender, SR_activity_responsibility] | "Mortality_rate_>young_SR"*"Adult_nanny_>12_months_infected" | OUTFLOW PRIORITY: 2 | Goat/Weeks |  | UNIFLOW |
| "Adult_nanny_>_12_months_infection_rate"[HHD_Gender, SR_activity_responsibility] | ("Seroprevalence_Adult_goat_>12_months"*Contact_rate*"Adult_nanny_>_12_months_susceptible")*("Adult_nanny_>12_months_infected"/Total_goat) | OUTFLOW PRIORITY: 1 | Goat/Weeks |  | UNIFLOW |
| "Adult_nanny_>_12_months_recovery_rate"[HHD_Gender, SR_activity_responsibility] | "Adult_nanny_>12_months_infected"/Average_PPR_illness_duration | OUTFLOW PRIORITY: 1 | Goat/Weeks |  | UNIFLOW |
| "Adult_nanny_>12_months_vaccinating"[HHD_Gender, SR_activity_responsibility] | (Disease_Control_Module."Sero-conversion_rate"*Disease_Control_Module."Adult_nanny_>_12_months_vaccination_rate")+"Becoming_adult_nanny_>_12_months_vaccinated_unidentified" | OUTFLOW PRIORITY: 2 | Goat/Weeks |  | UNIFLOW |
| Adult_nanny_stolen[HHD_Gender, SR_activity_responsibility] | Number_stolen_goat | OUTFLOW PRIORITY: 3 | Goat/Weeks |  | UNIFLOW |
| Adult_nanny_vaccinated_becoming_recovered[HHD_Gender, SR_activity_responsibility] | "Adult_nanny_>12_months_vaccinated"/Average_PPR_illness_duration |  | Goat/Weeks |  | UNIFLOW |
| "Adult_Ram_>_12_months_natural_recovery_rate"[HHD_Gender, SR_activity_responsibility] | "Adult_Ram_>_12_months_infected"/Average_PPR_illness_duration | OUTFLOW PRIORITY: 2 | Sheep per week |  | UNIFLOW |
| "Adult_Ram_>_12_months_vaccinated_recovery_rate"[HHD_Gender, SR_activity_responsibility] | "Adult_Ram_>_12_months_vaccinated"/vaccine_recovery_duration |  | Sheep per week |  | UNIFLOW |
| "Adult_Ram_>12_months_infection_rate"[HHD_Gender, SR_activity_responsibility] | (Contact_rate*"Seroprevalence_Adult_sheep_>12_months"*"Adult_Ram_>12_months_susceptible")*("Adult_Ram_>_12_months_infected"/Total_sheep) | OUTFLOW PRIORITY: 1 | Sheep per week |  | UNIFLOW |
| "Adult_Ram_>12_months_stolen"[HHD_Gender, SR_activity_responsibility] | Number_stolen | OUTFLOW PRIORITY: 3 | Sheep per week |  | UNIFLOW |
| "Adult_Ram_>12_months_vaccinating"[HHD_Gender, SR_activity_responsibility] | (Disease_Control_Module."Adult_Ram_>12_months_vaccination_rate"*Disease_Control_Module."Sero-conversion_rate")+Becoming_Adult_Ram_vaccinated_but_unidentified | OUTFLOW PRIORITY: 2 | Sheep per week |  | UNIFLOW |
| Adult_Ram_Deaths[HHD_Gender, SR_activity_responsibility] | "Mortality_rate_>young_SR"*"Adult_Ram_>_12_months_infected" | OUTFLOW PRIORITY: 1 | Sheep per week |  | UNIFLOW |
| Adulting_nanny_becoming_vulnerable[HHD_Gender, SR_activity_responsibility] | (Grower_nanny_6_to_12_months_recovered*(1-Grower_nanny_gifting_rate))/Time_for_goat_to_become_adult {Goat per week} | OUTFLOW PRIORITY: 2 | Goat/Weeks |  | UNIFLOW |
| "Becoming_adult_billy_>_12_months_vaccinated_identified"[HHD_Gender, SR_activity_responsibility] | (Grower_billy_6_to_12_months_vaccinated_recovered/Time_for_goat_to_become_adult)*(1-Proportion_wrongly_identified) | OUTFLOW PRIORITY: 1 | Goat/Weeks |  | UNIFLOW |
| Becoming_Adult_billy_natural_recovered[HHD_Gender, SR_activity_responsibility] | (Grower_billy_6_to_12_months_recovered*(1-Young_billy_gifting_rate))/Time_for_goat_to_become_adult {Goat per week} | OUTFLOW PRIORITY: 2 | Goat/Weeks |  | UNIFLOW |
| Becoming_Adult_Ewe_natural_recovered[HHD_Gender, SR_activity_responsibility] | (Grower_Ewe_6_to_12_months_natural_recovered*(1-Young_ewe_gifting_rate))/Time_for_sheep_to_become_Adult {Sheep per week} | OUTFLOW PRIORITY: 2 | Sheep per week |  | UNIFLOW |
| Becoming_Adult_Ewe_Vaccinated_identified[HHD_Gender, SR_activity_responsibility] | (Grower_Ewe_6_to_12_months/Time_for_sheep_to_become_Adult)*(1-Proportion_wrongly_identified) | OUTFLOW PRIORITY: 1 | Sheep per week |  | UNIFLOW |
| Becoming_Adult_Ewe_vaccinated_unidentified[HHD_Gender, SR_activity_responsibility] | (Grower_Ewe_6_to_12_months/Time_for_sheep_to_become_Adult)*Proportion_wrongly_identified | OUTFLOW PRIORITY: 2 | Sheep per week |  | UNIFLOW |
| "Becoming_adult_nanny_>_12_months_vaccinated"[HHD_Gender, SR_activity_responsibility] | Grower_nanny_6_to_12_months_vaccinated_recovered/Time_for_goat_to_become_adult | OUTFLOW PRIORITY: 1 | Goat/Weeks |  | UNIFLOW |
| "Becoming_adult_nanny_>_12_months_vaccinated_unidentified"[HHD_Gender, SR_activity_responsibility] | (Grower_nanny_6_to_12_months_vaccinated_recovered/Time_for_goat_to_become_adult)*Proportion_wrongly_identified | OUTFLOW PRIORITY: 2 | Goat/Weeks |  | UNIFLOW |
| Becoming_Adult_Ram_natural_recovery[HHD_Gender, SR_activity_responsibility] | Grower_Rams_6_to_12_months_natural_recovered/Time_for_sheep_to_become_Adult{Sheep per week} |  | Sheep per week |  | UNIFLOW |
| Becoming_Adult_Ram_vaccinated_but_unidentified[HHD_Gender, SR_activity_responsibility] | (Grower_Rams_6_to_12_months/Time_for_sheep_to_become_Adult)*Proportion_wrongly_identified | OUTFLOW PRIORITY: 3 | Sheep per week |  | UNIFLOW |
| Becoming_Adult_Ram_vaccinated_identified[HHD_Gender, SR_activity_responsibility] | (Grower_Rams_6_to_12_months/Time_for_sheep_to_become_Adult)*(1-Proportion_wrongly_identified) | OUTFLOW PRIORITY: 1 | Sheep per week |  | UNIFLOW |
| Becoming_adult_vaccinated_unidentified[HHD_Gender, SR_activity_responsibility] | (Grower_billy_6_to_12_months_vaccinated_recovered/Time_for_goat_to_become_adult)*Proportion_wrongly_identified | OUTFLOW PRIORITY: 2 | Goat/Weeks |  | UNIFLOW |
| Billy_given_out[HHD_Gender, SR_activity_responsibility] | (Grower_billy_6_to_12_months_recovered*Young_billy_gifting_rate*Frequency_of_gifting)/Time_to_gift_goat | OUTFLOW PRIORITY: 1 | Goat/Weeks |  | UNIFLOW |
| Billy_received_from_external_sources[HHD_Gender, SR_activity_responsibility] | (Average_billy_received*Frequency_of_gifting)/Time_to_receive_Adult_ram{Goats per weeks} |  | Goat/Weeks |  | UNIFLOW |
| Breeding_Ewes_natural_recovered[HHD_Gender, SR_activity_responsibility] | "Adult_Ewe_>12_months_recovered"/Time_ewe_kept {Sheep per weeks} |  | Sheep per week |  | UNIFLOW |
| Breeding_Ewes_vaccine_antibodies[HHD_Gender, SR_activity_responsibility] | "Adult_Ewe_>12_months"/Time_ewe_kept | OUTFLOW PRIORITY: 1 | Sheep per week |  | UNIFLOW |
| Breeding_nannies[HHD_Gender, SR_activity_responsibility] | "Adult_nanny_>12_months_recovered"*Percentage_of_nannies_reserved_for_breeding_annually {goats per week} |  | Goat/Weeks |  | UNIFLOW |
| Cull_selling_adult_nanny[HHD_Gender, SR_activity_responsibility] | (Bred_back_Nanny*Cull_out_rate_adult_nanny)/Time_to_sell_bred_back_nanny | OUTFLOW PRIORITY: 2 | Goat/Weeks |  | UNIFLOW |
| Ewe_given_out[HHD_Gender, SR_activity_responsibility] | (Grower_Ewe_6_to_12_months_natural_recovered*Young_ewe_gifting_rate*Frequency_of_gifting)/Time_to_gift_sheep | OUTFLOW PRIORITY: 1 | Sheep per week |  | UNIFLOW |
| Ewe_received_from_external_sources[HHD_Gender, SR_activity_responsibility] | (Average_ewe_received*Frequency_of_gifting)/ Time_to_receive_Adult_ram |  | Sheep per week |  | UNIFLOW |
| Grower_3_to_6_months_stolen[HHD_Gender, SR_activity_responsibility] | Number_stolen | OUTFLOW PRIORITY: 3 | Sheep per week |  | UNIFLOW |
| Grower_billy_6_to_12_months_infection_rate[HHD_Gender, SR_activity_responsibility] | (Contact_rate*Seroprevalence_Grower_goat_6_to_12_months*Grower_billy_6_to_12_months_susceptible)*(Grower_billy_6_to_12_months_infected/Total_goat) | OUTFLOW PRIORITY: 1 | Goat/Weeks |  | UNIFLOW |
| Grower_billy_6_to_12_months_recovery_rate[HHD_Gender, SR_activity_responsibility] | Grower_billy_6_to_12_months_infected/Average_PPR_illness_duration | OUTFLOW PRIORITY: 1 | Goat/Weeks |  | UNIFLOW |
| Grower_billy_6_to_12_months_vaccinated_recovery_rate[HHD_Gender, SR_activity_responsibility] | Grower_billy_6_to_12_months_vaccinated/vaccine_recovery_duration |  | Goat/Weeks |  | UNIFLOW |
| Grower_billy_6_to_12_months_vaccinating[HHD_Gender, SR_activity_responsibility] | (Disease_Control_Module."Sero-conversion_rate"*Disease_Control_Module.Grower_billy_6_to_12_months_vaccination_rate)+Growing_billy_vaccinated_unidentified | OUTFLOW PRIORITY: 2 | Goat/Weeks |  | UNIFLOW |
| Grower_billy_stolen[HHD_Gender, SR_activity_responsibility] | Number_stolen_goat | OUTFLOW PRIORITY: 3 | Goat/Weeks |  | UNIFLOW |
| Grower_Ewe_6_to_12_months_infection_rate[HHD_Gender, SR_activity_responsibility] | (Contact_rate*Seroprevalence_Grower_sheep_6_to_12_months*Grower_Ewe_6_to_12_months_susceptible)*(Grower_Ewe_6_to_12_months_infected/Total_sheep) | OUTFLOW PRIORITY: 2 | Sheep per week |  | UNIFLOW |
| Grower_Ewe_6_to_12_months_natural_recovery_rate[HHD_Gender, SR_activity_responsibility] | Grower_Ewe_6_to_12_months_infected/Average_PPR_illness_duration | OUTFLOW PRIORITY: 1 | Sheep per week |  | UNIFLOW |
| Grower_Ewe_6_to_12_months_stolen[HHD_Gender, SR_activity_responsibility] | Number_stolen | OUTFLOW PRIORITY: 3 | Sheep per week |  | UNIFLOW |
| Grower_Ewe_6_to_12_months_vaccinated_recovery_rate[HHD_Gender, SR_activity_responsibility] | Grower_Ewe_6_to_12_months_vaccinated/vaccine_recovery_duration |  | Sheep per week |  | UNIFLOW |
| Grower_Ewe_6_to_12_months_vaccinating[HHD_Gender, SR_activity_responsibility] | (Disease_Control_Module.Grower_Ewes_6_to_12_months_vaccination_rate*Disease_Control_Module."Sero-conversion_rate")+Growing_Ewe_vaccinated_but_unidentified | OUTFLOW PRIORITY: 1 | Sheep per week |  | UNIFLOW |
| Grower_Ewes_dying[HHD_Gender, SR_activity_responsibility] | Grower_Ewe_6_to_12_months_infected*"Mortality_rate_>young_SR" | OUTFLOW PRIORITY: 2 | Sheep per week |  | UNIFLOW |
| Grower_nanny_6_to_12_months_infection_rate[HHD_Gender, SR_activity_responsibility] | (Contact_rate*Seroprevalence_Grower_goat_6_to_12_months*Grower_nanny_6_to_12_months_susceptible)*(Grower_nanny_6_to_12_months_infected/Total_goat) | OUTFLOW PRIORITY: 2 | Goat/Weeks |  | UNIFLOW |
| Grower_nanny_6_to_12_months_recovery_rate[HHD_Gender, SR_activity_responsibility] | Grower_nanny_6_to_12_months_infected/Average_PPR_illness_duration | OUTFLOW PRIORITY: 2 | Goat/Weeks |  | UNIFLOW |
| Grower_nanny_6_to_12_months_vaccinated_recovery_rate[HHD_Gender, SR_activity_responsibility] | Grower_nanny_6_to_12_months_vaccinated/vaccine_recovery_duration |  | Goat/Weeks |  | UNIFLOW |
| Grower_nanny_6_to_12_months_vaccinating[HHD_Gender, SR_activity_responsibility] | (Disease_Control_Module.Grower_nanny_6_to_12_months_vaccination_rate*Disease_Control_Module."Sero-conversion_rate")+Growing_nanny_vaccinated_unidentified | OUTFLOW PRIORITY: 1 | Goat/Weeks |  | UNIFLOW |
| Grower_nanny_Dying[HHD_Gender, SR_activity_responsibility] | Grower_nanny_6_to_12_months_infected*"Mortality_rate_>young_SR" | OUTFLOW PRIORITY: 1 | Goat/Weeks |  | UNIFLOW |
| Grower_nanny_stolen[HHD_Gender, SR_activity_responsibility] | Number_stolen_goat | OUTFLOW PRIORITY: 3 | Goat/Weeks |  | UNIFLOW |
| Grower_Ram_given_out[HHD_Gender, SR_activity_responsibility] | (Grower_Rams_6_to_12_months*Proportion_of_vaccinated_grower_ram_given_out*Frequency_of_gifting)/Time_to_gift_sheep | OUTFLOW PRIORITY: 2 | Sheep per week |  | UNIFLOW |
| Grower_Rams_6_to_12_months_infection_rate[HHD_Gender, SR_activity_responsibility] | (Contact_rate*Seroprevalence_Grower_sheep_6_to_12_months*Grower_Rams_6_to_12_months_Susceptible)*(Grower_Rams_6_to_12_months_infected/Total_sheep) | OUTFLOW PRIORITY: 1 | Sheep per week |  | UNIFLOW |
| Grower_Rams_6_to_12_months_natural_recovery_rate[HHD_Gender, SR_activity_responsibility] | Grower_Rams_6_to_12_months_infected/Average_PPR_illness_duration | OUTFLOW PRIORITY: 2 | Sheep per week |  | UNIFLOW |
| Grower_Rams_6_to_12_months_vaccinated_recovery_rate[HHD_Gender, SR_activity_responsibility] | Grower_Rams_6_to_12_months_vaccinated/vaccine_recovery_duration |  | Sheep per week |  | UNIFLOW |
| Grower_Rams_6_to_12_months_vaccinating[HHD_Gender, SR_activity_responsibility] | (Disease_Control_Module.Grower_Rams_6_to_12_months_vaccination_rate*Disease_Control_Module."Sero-conversion_rate")+Growing_Ram_vaccinated_but_unidentified | OUTFLOW PRIORITY: 2 | Sheep per week |  | UNIFLOW |
| Growers_billy_Dying[HHD_Gender, SR_activity_responsibility] | Grower_billy_6_to_12_months_infected*"Mortality_rate_>young_SR" | OUTFLOW PRIORITY: 2 | Goat/Weeks |  | UNIFLOW |
| Growers_Ram_Deaths[HHD_Gender, SR_activity_responsibility] | Grower_Rams_6_to_12_months_infected*"Mortality_rate_>young_SR" | OUTFLOW PRIORITY: 1 | Sheep per week |  | UNIFLOW |
| Growing_billy_natural_recovered[HHD_Gender, SR_activity_responsibility] | (Young_billy_3_to_6_months_recovered*(1-Young_billy_selling_'rate))/Time_for_goat_to_grow {Goat per week} | OUTFLOW PRIORITY: 2 | Goat/Weeks |  | UNIFLOW |
| Growing_billy_vaccinated_identified[HHD_Gender, SR_activity_responsibility] | (Young_billy_3_to_6_months_vaccinated_recovered/Time_for_goat_to_grow)*(1-Proportion_wrongly_identified) | OUTFLOW PRIORITY: 1 | Goat/Weeks |  | UNIFLOW |
| Growing_billy_vaccinated_unidentified[HHD_Gender, SR_activity_responsibility] | (Young_billy_3_to_6_months_vaccinated_recovered/Time_for_goat_to_grow)*Proportion_wrongly_identified | OUTFLOW PRIORITY: 2 | Goat/Weeks |  | UNIFLOW |
| Growing_Ewe_natural_recovered[HHD_Gender, SR_activity_responsibility] | (Young_Ewe_3_to_6_months_natural_recovered*(1-Offtake_rate_Young_Ewe))/Time_for_sheep_to_grow {Sheep per week} | OUTFLOW PRIORITY: 1 | Sheep per week |  | UNIFLOW |
| Growing_Ewe_vaccinated_but_unidentified[HHD_Gender, SR_activity_responsibility] | (Young_Ewe_3_to_6_months_vaccinated_recovered/Time_for_sheep_to_grow)*Proportion_wrongly_identified | OUTFLOW PRIORITY: 2 | Sheep per week |  | UNIFLOW |
| Growing_Ewe_vaccinated_identified[HHD_Gender, SR_activity_responsibility] | ((Young_Ewe_3_to_6_months_vaccinated_recovered*(1-Offtake_rate_Young_Ewe))/Time_for_sheep_to_grow)/(1-Proportion_wrongly_identified) | OUTFLOW PRIORITY: 1 | Sheep per week |  | UNIFLOW |
| Growing_nanny_becoming_vulnerable[HHD_Gender, SR_activity_responsibility] | (Young_nanny_3_to_6_months_recovered*(1-Offtake_rate_Young_nanny))/Time_for_goat_to_grow {Sheep per week} | OUTFLOW PRIORITY: 2 | Goat/Weeks |  | UNIFLOW |
| Growing_nanny_vaccinated_identified[HHD_Gender, SR_activity_responsibility] | (Young_nanny_3_to_6_months_vaccinated_recovered/Time_for_goat_to_grow)*(1-Proportion_wrongly_identified) | OUTFLOW PRIORITY: 1 | Goat/Weeks |  | UNIFLOW |
| Growing_nanny_vaccinated_unidentified[HHD_Gender, SR_activity_responsibility] | (Young_nanny_3_to_6_months_vaccinated_recovered/Time_for_goat_to_grow)*Proportion_wrongly_identified | OUTFLOW PRIORITY: 2 | Goat/Weeks |  | UNIFLOW |
| Growing_Ram_natural_recovery[HHD_Gender, SR_activity_responsibility] | (Young_Ram_3_to_6_months_natural_recovered*(1-Proportion_sold_young_ram_3_to_6_months_natural_recovered))/Time_for_sheep_to_grow {Sheep per week} | OUTFLOW PRIORITY: 2 | Sheep per week |  | UNIFLOW |
| Growing_Ram_vaccinated_but_unidentified[HHD_Gender, SR_activity_responsibility] | (Young_Ram_3_to_6_months_vaccinated_recovered/Time_for_sheep_to_grow)*Proportion_wrongly_identified | OUTFLOW PRIORITY: 3 | Sheep per week |  | UNIFLOW |
| Growing_Ram_vaccinated_identified[HHD_Gender, SR_activity_responsibility] | (Young_Ram_3_to_6_months_vaccinated_recovered/Time_for_sheep_to_grow)*(1-Proportion_wrongly_identified) | OUTFLOW PRIORITY: 2 | Sheep per week |  | UNIFLOW |
| HH_consumption_billy[HHD_Gender, SR_activity_responsibility] | (HH_billy_Consumption_rate*"Adult_billy_>12_months_recovered")/Time_HH_slaughter{Sheep per weeks} | OUTFLOW PRIORITY: 2 | Goat/Weeks |  | UNIFLOW |
| HH_consumption_Ram[HHD_Gender, SR_activity_responsibility] | (HH_Ram_Consumption_rate*"Adult_Ram_>12_months_Recovered")/Time_of_HH_Ram_Consumption | OUTFLOW PRIORITY: 1 | Sheep per week |  | UNIFLOW |
| HH_Ewe_Consumption[HHD_Gender, SR_activity_responsibility] | (Bred_back_Ewes*HH_Ewe_Consumption_rate)/ Time_for_HH_ewe_slaughter | OUTFLOW PRIORITY: 1 | Sheep per week |  | UNIFLOW |
| HH_nanny_Consumption[HHD_Gender, SR_activity_responsibility] | (Bred_back_Nanny*HH_nanny_Consumption_rate)/HH_nanny_consumption_time {Goats per week} | OUTFLOW PRIORITY: 1 | Goat/Weeks |  | UNIFLOW |
| "Kid_>3_months_Dying"[HHD_Gender, SR_activity_responsibility] | (Young_kids_mortality_rate*"Kid_>3_months")/Time_being_kid | OUTFLOW PRIORITY: 3 | Goat/Weeks |  | UNIFLOW |
| Kid_Breeding_rate[HHD_Gender, SR_activity_responsibility] | (Bred_back_Nanny*Fertilization_success_goat*Adult_nanny_pregnancy_percentage*Kid_per_parturition)/52 |  | Goat/Weeks |  | UNIFLOW |
| Kid_Still_born_rate[HHD_Gender, SR_activity_responsibility] | DELAY3((Kid_Breeding_rate*Fractional_abortion_rate_goat), Gestation_period_Goat) {Goat per week} | OUTFLOW PRIORITY: 1 | Goat/Weeks |  | UNIFLOW |
| Kidding[HHD_Gender, SR_activity_responsibility] | DELAY3((Kid_Breeding_rate*Goat_Survival_rate), Gestation_period_Goat) | OUTFLOW PRIORITY: 2 | Goat/Weeks |  | UNIFLOW |
| "Lamb_>3_months_Dying"[HHD_Gender, SR_activity_responsibility] | ("Lambs_>3_months"*Infant_sheep_mortality_rate)/Time_being_lamb | OUTFLOW PRIORITY: 1 | Sheep per week |  | UNIFLOW |
| Lamb_Breeding_rate[HHD_Gender, SR_activity_responsibility] | (Bred_back_Ewes*Fertilization_success_sheep*Adult_Ewe_Pregnancy_percentage*Lamb_per_parturition)/52 {Sheep per weeks} |  | Sheep per week |  | UNIFLOW |
| Lambing[HHD_Gender, SR_activity_responsibility] | DELAY3((Lamb_Breeding_rate*Sheep_Survival_rate), Gestation_period_sheep) | OUTFLOW PRIORITY: 2 | Sheep per week |  | UNIFLOW |
| Nanny_given_out[HHD_Gender, SR_activity_responsibility] | (Grower_nanny_6_to_12_months_recovered*Grower_nanny_gifting_rate*Frequency_of_gifting)/Time_to_gift_goat | OUTFLOW PRIORITY: 1 | Goat/Weeks |  | UNIFLOW |
| Nanny_received_from_external_sources[HHD_Gender, SR_activity_responsibility] | (Average_nanny_received*Frequency_of_gifting)/Time_to_receive_Adult_ram {Goats per week} |  | Goat/Weeks |  | UNIFLOW |
| Ram_received_from_external_sources[HHD_Gender, SR_activity_responsibility] | (Average_ram_received*Frequency_of_gifting)/ Time_to_receive_Adult_ram |  | Sheep per week |  | UNIFLOW |
| Ram_Still_born_rate[HHD_Gender, SR_activity_responsibility] | DELAY3((Lamb_Breeding_rate*Fractional_abortion_rate_sheep), Gestation_period_sheep) {Sheep per week} | OUTFLOW PRIORITY: 1 | Sheep per week |  | UNIFLOW |
| Reserved_billy_for_breeding[HHD_Gender, SR_activity_responsibility] | (Billy_reserve_breeding_rate*"Adult_billy_>12_months_recovered")/Time_to_sell_reserved_billy_for_breeding {goat per week} | OUTFLOW PRIORITY: 3 | Goat/Weeks |  | UNIFLOW |
| Reserved_ram_for_breeding[HHD_Gender, SR_activity_responsibility] | (Ram_reserve_breeding_rate*"Adult_Ram_>12_months_Recovered")/Time_for_ram_to_start_breeding {Sheep per week} | OUTFLOW PRIORITY: 2 | Sheep per week |  | UNIFLOW |
| Selling_Adult_billy[HHD_Gender, SR_activity_responsibility] | (Offtake_Rate_Adult_billy*"Adult_billy_>12_months_recovered")/Time_to_sell_adult_billy{Sheep per weeks} | OUTFLOW PRIORITY: 1 | Goat/Weeks |  | UNIFLOW |
| Selling_Adult_Ram[HHD_Gender, SR_activity_responsibility] | (Offtake_Rate_Adult_Ram*"Adult_Ram_>12_months_Recovered")/Time_to_sell_adult_ram{Sheep per weeks} | OUTFLOW PRIORITY: 3 | Sheep per week |  | UNIFLOW |
| Selling_Cull_Adult_Ewe[HHD_Gender, SR_activity_responsibility] | (Bred_back_Ewes*Cull_out_rate_Adult_Ewe)/ Time_ewe_kept | OUTFLOW PRIORITY: 2 | Sheep per week |  | UNIFLOW |
| Selling_of_adult_billy_reserved_for_breeding[HHD_Gender, SR_activity_responsibility] | Adult_Buck_for_breeding*Offtake_rate_reserved_adult_billy/ Time_to_sell_reserved_billy_for_breeding |  | Goat/Weeks |  | UNIFLOW |
| Selling_Reserved_Adult_Ram[HHD_Gender, SR_activity_responsibility] | (Adult_Ram_for_breeding*Cull_out_rate_Reserved_Adult_Ram)/Time_to_sell_reserved_ram_for_breeding {Sheep per week} |  | Sheep per week |  | UNIFLOW |
| Weaning_billy_goat[HHD_Gender, SR_activity_responsibility] | ("Kid_>3_months"*Billy_to_nanny_ratio)/Time_to_wean_young_goat | OUTFLOW PRIORITY: 1 | Goat/Weeks |  | UNIFLOW |
| Weaning_Ewe[HHD_Gender, SR_activity_responsibility] | ("Lambs_>3_months"*(1-Ram_to_Ewe_ratio))/Time_to_wean_young_lamb {Sheep per week} | OUTFLOW PRIORITY: 2 | Sheep per week |  | UNIFLOW |
| Weaning_nanny_goat[HHD_Gender, SR_activity_responsibility] | ("Kid_>3_months"*(1-Billy_to_nanny_ratio))/Time_to_wean_young_goat | OUTFLOW PRIORITY: 2 | Goat/Weeks |  | UNIFLOW |
| Weaning_Ram[HHD_Gender, SR_activity_responsibility] | ("Lambs_>3_months"*Ram_to_Ewe_ratio)/Time_to_wean_young_lamb | OUTFLOW PRIORITY: 3 | Sheep per week |  | UNIFLOW |
| Young_billy_3_to_6_months_infection_rate[HHD_Gender, SR_activity_responsibility] | (Contact_rate*Seroprevalence_young_goat_3_to_6_months*Young_billy_3_to_6_months_susceptible)*(Young_billy_3_to_6_months_infected/Total_goat) | OUTFLOW PRIORITY: 1 | Goat/Weeks |  | UNIFLOW |
| Young_billy_3_to_6_months_recovery_rate[HHD_Gender, SR_activity_responsibility] | Young_billy_3_to_6_months_infected/Average_PPR_illness_duration | OUTFLOW PRIORITY: 2 | Goat/Weeks |  | UNIFLOW |
| Young_billy_3_to_6_months_vaccinated_recovery_rate[HHD_Gender, SR_activity_responsibility] | Young_billy_3_to_6_months_vaccinated/vaccine_recovery_duration |  | Goat/Weeks |  | UNIFLOW |
| Young_billy_3_to_6_months_vaccinating[HHD_Gender, SR_activity_responsibility] | Disease_Control_Module."Sero-conversion_rate"*Disease_Control_Module.Young_billy_3_to_6_months_vaccination_rate | OUTFLOW PRIORITY: 2 | Goat/Weeks |  | UNIFLOW |
| Young_billy_Dying[HHD_Gender, SR_activity_responsibility] | Young_billy_3_to_6_months_infected*Mortality_rate_young_SR | OUTFLOW PRIORITY: 1 | Goat/Weeks |  | UNIFLOW |
| Young_billy_selling[HHD_Gender, SR_activity_responsibility] | (Young_billy_3_to_6_months_recovered*Young_billy_selling_'rate)/Time_to_sell_young_billy {Goat per week} | OUTFLOW PRIORITY: 1 | Goat/Weeks |  | UNIFLOW |
| Young_Ewe_3_to_6_months_dying[HHD_Gender, SR_activity_responsibility] | Young_Ewe_3_to_6_months_infected*Mortality_rate_young_SR | OUTFLOW PRIORITY: 1 | Sheep per week |  | UNIFLOW |
| Young_Ewe_3_to_6_months_infection_rate[HHD_Gender, SR_activity_responsibility] | (Contact_rate*Seroprevalence_Young_sheep_3_to_6_months*Young_Ewe_3_to_6_months_susceptible)*(Young_Ewe_3_to_6_months_infected/Total_sheep) | OUTFLOW PRIORITY: 1 | Sheep per week |  | UNIFLOW |
| Young_Ewe_3_to_6_months_natural_recovery_rate[HHD_Gender, SR_activity_responsibility] | Young_Ewe_3_to_6_months_infected/ Average_PPR_illness_duration | OUTFLOW PRIORITY: 2 | Sheep per week |  | UNIFLOW |
| Young_Ewe_3_to_6_months_stolen[HHD_Gender, SR_activity_responsibility] | Number_stolen | OUTFLOW PRIORITY: 3 | Sheep per week |  | UNIFLOW |
| Young_Ewe_3_to_6_months_vaccinated_recovery_rate[HHD_Gender, SR_activity_responsibility] | Young_Ewe_3_to_6_months_vaccinated/vaccine_recovery_duration |  | Sheep per week |  | UNIFLOW |
| Young_Ewe_3_to_6_months_vaccinating[HHD_Gender, SR_activity_responsibility] | Disease_Control_Module."Sero-conversion_rate"*Disease_Control_Module.Young_Ewe_3_to_6_months_Vaccination_rate | OUTFLOW PRIORITY: 2 | Sheep per week |  | UNIFLOW |
| Young_Ewe_selling[HHD_Gender, SR_activity_responsibility] | (Young_Ewe_3_to_6_months_natural_recovered*Offtake_rate_Young_Ewe)/Time_to_sell_young_lamb {Sheep per week} | OUTFLOW PRIORITY: 2 | Sheep per week |  | UNIFLOW |
| Young_kid_stolen[HHD_Gender, SR_activity_responsibility] | Number_stolen_goat | OUTFLOW PRIORITY: 3 | Goat/Weeks |  | UNIFLOW |
| Young_nanny_3_to_6_months_infection_rate[HHD_Gender, SR_activity_responsibility] | (Contact_rate*Seroprevalence_young_goat_3_to_6_months*Young_nanny_3_to_6_months_susceptible)*(Young_nanny_3_to_6_months_infected/Total_goat) | OUTFLOW PRIORITY: 1 | Goat/Weeks |  | UNIFLOW |
| Young_nanny_3_to_6_months_recovery_rate[HHD_Gender, SR_activity_responsibility] | Young_nanny_3_to_6_months_infected/Average_PPR_illness_duration | OUTFLOW PRIORITY: 2 | Goat/Weeks |  | UNIFLOW |
| Young_nanny_3_to_6_months_vaccinated_recovery_rate[HHD_Gender, SR_activity_responsibility] | Young_nanny_3_to_6_months_vaccinated/vaccine_recovery_duration |  | Goat/Weeks |  | UNIFLOW |
| Young_nanny_3_to_6_months_vaccinating[HHD_Gender, SR_activity_responsibility] | Disease_Control_Module.Young_nanny_3_to_6_months_vaccination_rate*Disease_Control_Module."Sero-conversion_rate" | OUTFLOW PRIORITY: 2 | Goat/Weeks |  | UNIFLOW |
| Young_Nanny_Dying[HHD_Gender, SR_activity_responsibility] | Mortality_rate_young_SR*Young_nanny_3_to_6_months_infected | OUTFLOW PRIORITY: 1 | Goat/Weeks |  | UNIFLOW |
| Young_nanny_Selling[HHD_Gender, SR_activity_responsibility] | (Young_nanny_3_to_6_months_recovered*Offtake_rate_Young_nanny)/Time_to_sell_young_billy {Goats per week} | OUTFLOW PRIORITY: 1 | Goat/Weeks |  | UNIFLOW |
| Young_nanny_stolen[HHD_Gender, SR_activity_responsibility] | Number_stolen_goat | OUTFLOW PRIORITY: 3 | Goat/Weeks |  | UNIFLOW |
| Young_Ram_3_to_6_months_infection_rate[HHD_Gender, SR_activity_responsibility] | (Contact_rate*Seroprevalence_Young_sheep_3_to_6_months*Young_Ram_3_to_6_months_susceptible)*(Young_Ram_3_to_6_months_infected/Total_sheep) | OUTFLOW PRIORITY: 3 | Sheep per week |  | UNIFLOW |
| Young_Ram_3_to_6_months_natural_recovered_sold[HHD_Gender, SR_activity_responsibility] | (Young_Ram_3_to_6_months_natural_recovered *Proportion_sold_young_ram_3_to_6_months_natural_recovered)/Time_to_sell_young_lamb | OUTFLOW PRIORITY: 1 | Sheep per week |  | UNIFLOW |
| Young_Ram_3_to_6_months_natural_recovery_rate[HHD_Gender, SR_activity_responsibility] | Young_Ram_3_to_6_months_infected/Average_PPR_illness_duration | OUTFLOW PRIORITY: 1 | Sheep per week |  | UNIFLOW |
| Young_ram_3_to_6_months_stolen[HHD_Gender, SR_activity_responsibility] | Number_stolen | OUTFLOW PRIORITY: 2 | Sheep per week |  | UNIFLOW |
| Young_Ram_3_to_6_months_vaccinated_recovered_selling[HHD_Gender, SR_activity_responsibility] | (Young_Ram_3_to_6_months_vaccinated_recovered*(1-Proportion_sold_young_ram_3_to_6_months_natural_recovered))/Time_to_sell_young_lamb | OUTFLOW PRIORITY: 1 | Sheep per week |  | UNIFLOW |
| Young_Ram_3_to_6_months_vaccinated_recovery_rate[HHD_Gender, SR_activity_responsibility] | Young_Ram_3_to_6_months_vaccinated/vaccine_recovery_duration |  | Sheep per week |  | UNIFLOW |
| Young_Ram_3_to_6_months_vaccinating[HHD_Gender, SR_activity_responsibility] | Disease_Control_Module.Young_Ram_3_to_6_months_Vaccination_rate*Disease_Control_Module."Sero-conversion_rate" | OUTFLOW PRIORITY: 1 | Sheep per week |  | UNIFLOW |
| Young_Ram_Dying[HHD_Gender, SR_activity_responsibility] | Young_Ram_3_to_6_months_infected*Mortality_rate_young_SR | OUTFLOW PRIORITY: 2 | Sheep per week |  | UNIFLOW |
| Young_sheep_received_as_gift[HHD_Gender, SR_activity_responsibility] | (Average_young_sheep_received*Frequency_of_gifting)/Time_to_receive_young_sheep |  | Sheep per week |  | UNIFLOW |
| Adult_Ewe_Pregnancy_percentage[HHD_Gender, SR_activity_responsibility] | 1*Animal_husbandry_effect_on_productivity {unitless} |  | Dimensionless |  |  |
| Adult_nanny_pregnancy_percentage[HHD_Gender, SR_activity_responsibility] | 1*Animal_husbandry_effect_on_productivity {unitless} |  | Dimensionless |  |  |
| Animal_husbandry_effect_on_productivity[HHD_Gender, SR_activity_responsibility] | IF(Feeding_ration < Required_feed_ration) THEN 1 ELSE 0.5 |  | Dimensionless |  |  |
| Average_billy_received[Male_HHD, HHD] | 1 {HH survey data} |  | Goat |  |  |
| Average_billy_received[Male_HHD, Spouse] | 0 {HH survey data} |  |  |  |  |
| Average_billy_received[Female_HHD, HHD] | 1 {HH survey data} |  |  |  |  |
| Average_billy_received[Female_HHD, Spouse] | 0 {HH survey data} |  |  |  |  |
| Average_ewe_received[Male_HHD, HHD] | 0 {HH survey data} |  | Sheep |  |  |
| Average_ewe_received[Male_HHD, Spouse] | 1 |  |  |  |  |
| Average_ewe_received[Female_HHD, HHD] | 1 {HH survey dat} |  |  |  |  |
| Average_ewe_received[Female_HHD, Spouse] | 0 {HH survey data} |  |  |  |  |
| Average_nanny_received[Male_HHD, HHD] | 0 {HH survey data} |  | Goat |  |  |
| Average_nanny_received[Male_HHD, Spouse] | 1{HH survey data} |  |  |  |  |
| Average_nanny_received[Female_HHD, HHD] | 0 |  |  |  |  |
| Average_nanny_received[Female_HHD, Spouse] | 1 {HH survey data} |  |  |  |  |
| Average_PPR_illness_duration | 3 {OIE, 2013 - incubation period for Terrestrial Animal Health code} |  | Weeks |  |  |
| Average_ram_received[Male_HHD, HHD] | 1 {HH survey dat} |  | Sheep |  |  |
| Average_ram_received[Male_HHD, Spouse] | 1 {HH survey data} |  |  |  |  |
| Average_ram_received[Female_HHD, HHD] | 1 {HH survey data} |  |  |  |  |
| Average_ram_received[Female_HHD, Spouse] | 0 {HH survey data} |  |  |  |  |
| Average_young_sheep_received[Male_HHD, HHD] | 0 {HH survey data} |  | Sheep |  |  |
| Average_young_sheep_received[Male_HHD, Spouse] | 0 {HH survey data} |  |  |  |  |
| Average_young_sheep_received[Female_HHD, HHD] | 0 {HH survey data} |  |  |  |  |
| Average_young_sheep_received[Female_HHD, Spouse] | 1 {HH survey data} |  |  |  |  |
| Billy_reserve_breeding_rate | Offtake_Rate_Adult_billy*HH_billy_Consumption_rate {unitless} |  | Dimensionless |  |  |
| Billy_to_nanny_ratio | 0.8 {HH survey data} |  | Dimensionless |  |  |
| Contact_rate | 1 |  | Dimensionless |  |  |
| Cull_out_rate_Adult_Ewe | GRAPH(TIME) Points: (1.0, 0.000), (3.03529411765, 0.000), (5.07058823529, 0.000), (7.10588235294, 0.000), (9.14117647059, 0.000), (11.1764705882, 0.000), (13.2117647059, 0.000), (15.2470588235, 0.000), (17.2823529412, 0.000), (19.3176470588, 0.000), (21.3529411765, 0.000), (23.3882352941, 0.000), (25.4235294118, 0.000), (27.4588235294, 0.000), (29.4941176471, 0.000), (31.5294117647, 0.000), (33.5647058824, 0.000), (35.6, 0.000), (37.6352941176, 0.000), (39.6705882353, 0.000), (41.7058823529, 0.000), (43.7411764706, 0.000), (45.7764705882, 0.000), (47.8117647059, 0.000), (49.8470588235, 0.000), (51.8823529412, 0.000), (53.9176470588, 0.000), (55.9529411765, 0.000), (57.9882352941, 0.000), (60.0235294118, 0.000), (62.0588235294, 0.000), (64.0941176471, 0.000), (66.1294117647, 0.000), (68.1647058824, 0.000), (70.2, 0.000), (72.2352941176, 0.000), (74.2705882353, 0.000), (76.3058823529, 0.000), (78.3411764706, 0.000), (80.3764705882, 0.000), (82.4117647059, 0.000), (84.4470588235, 0.000), (86.4823529412, 0.000), (88.5176470588, 0.000), (90.5529411765, 0.000), (92.5882352941, 0.000), (94.6235294118, 0.000), (96.6588235294, 0.000), (98.6941176471, 0.000), (100.729411765, 0.000), (102.764705882, 0.000), (104.8, 0.000), (106.835294118, 0.000), (108.870588235, 0.000), (110.905882353, 0.000), (112.941176471, 0.000), (114.976470588, 0.000), (117.011764706, 0.000), (119.047058824, 0.000), (121.082352941, 0.000), (123.117647059, 0.000), (125.152941176, 0.000), (127.188235294, 0.000), (129.223529412, 0.000), (131.258823529, 0.000), (133.294117647, 0.000), (135.329411765, 1.000), (137.364705882, 0.000), (139.4, 0.000), (141.435294118, 0.000), (143.470588235, 0.000), (145.505882353, 0.000), (147.541176471, 0.000), (149.576470588, 0.000), (151.611764706, 0.000), (153.647058824, 0.000), (155.682352941, 0.000), (157.717647059, 0.000), (159.752941176, 0.000), (161.788235294, 0.000), (163.823529412, 0.000), (165.858823529, 0.000), (167.894117647, 0.000), (169.929411765, 0.000), (171.964705882, 0.000), (174.0, 0.000), (176.035294118, 0.000), (178.070588235, 0.000), (180.105882353, 0.000), (182.141176471, 0.000), (184.176470588, 0.000), (186.211764706, 0.000), (188.247058824, 0.000), (190.282352941, 0.000), (192.317647059, 0.000), (194.352941176, 0.000), (196.388235294, 0.000), (198.423529412, 0.000), (200.458823529, 0.000), (202.494117647, 0.000), (204.529411765, 0.000), (206.564705882, 0.000), (208.6, 0.000), (210.635294118, 0.000), (212.670588235, 0.000), (214.705882353, 0.000), (216.741176471, 0.000), (218.776470588, 0.000), (220.811764706, 0.000), (222.847058824, 0.000), (224.882352941, 0.000), (226.917647059, 0.000), (228.952941176, 0.000), (230.988235294, 0.000), (233.023529412, 0.000), (235.058823529, 0.000), (237.094117647, 0.000), (239.129411765, 0.000), (241.164705882, 0.000), (243.2, 0.000), (245.235294118, 0.000), (247.270588235, 0.000), (249.305882353, 0.000), (251.341176471, 0.000), (253.376470588, 0.000), (255.411764706, 0.000), (257.447058824, 0.000), (259.482352941, 0.000), (261.517647059, 0.000), (263.552941176, 0.000), (265.588235294, 0.000), (267.623529412, 0.000), (269.658823529, 0.000), (271.694117647, 1.000), (273.729411765, 0.000), (275.764705882, 0.000), (277.8, 0.000), (279.835294118, 0.000), (281.870588235, 0.000), (283.905882353, 0.000), (285.941176471, 0.000), (287.976470588, 0.000), (290.011764706, 0.000), (292.047058824, 0.000), (294.082352941, 0.000), (296.117647059, 0.000), (298.152941176, 0.000), (300.188235294, 0.000), (302.223529412, 0.000), (304.258823529, 0.000), (306.294117647, 0.000), (308.329411765, 0.000), (310.364705882, 0.000), (312.4, 0.000), (314.435294118, 0.000), (316.470588235, 0.000), (318.505882353, 0.000), (320.541176471, 0.000), (322.576470588, 0.000), (324.611764706, 0.000), (326.647058824, 0.000), (328.682352941, 0.000), (330.717647059, 0.000), (332.752941176, 0.000), (334.788235294, 0.000), (336.823529412, 0.000), (338.858823529, 0.000), (340.894117647, 0.000), (342.929411765, 0.000), (344.964705882, 0.000), (347.0, 0.000), (349.035294118, 0.000), (351.070588235, 0.000), (353.105882353, 0.000), (355.141176471, 0.000), (357.176470588, 0.000), (359.211764706, 0.000), (361.247058824, 0.000), (363.282352941, 0.000), (365.317647059, 0.000), (367.352941176, 0.000), (369.388235294, 0.000), (371.423529412, 0.000), (373.458823529, 0.000), (375.494117647, 0.000), (377.529411765, 0.000), (379.564705882, 0.000), (381.6, 0.000), (383.635294118, 0.000), (385.670588235, 0.000), (387.705882353, 0.000), (389.741176471, 0.000), (391.776470588, 0.000), (393.811764706, 0.000), (395.847058824, 0.000), (397.882352941, 0.000), (399.917647059, 0.000), (401.952941176, 0.000), (403.988235294, 0.000), (406.023529412, 1.000), (408.058823529, 0.000), (410.094117647, 0.000), (412.129411765, 0.000), (414.164705882, 0.000), (416.2, 0.000), (418.235294118, 0.000), (420.270588235, 0.000), (422.305882353, 0.000), (424.341176471, 0.000), (426.376470588, 0.000), (428.411764706, 0.000), (430.447058824, 0.000), (432.482352941, 0.000), (434.517647059, 0.000), (436.552941176, 0.000), (438.588235294, 0.000), (440.623529412, 0.000), (442.658823529, 0.000), (444.694117647, 0.000), (446.729411765, 0.000), (448.764705882, 0.000), (450.8, 0.000), (452.835294118, 0.000), (454.870588235, 0.000), (456.905882353, 0.000), (458.941176471, 0.000), (460.976470588, 0.000), (463.011764706, 0.000), (465.047058824, 0.000), (467.082352941, 0.000), (469.117647059, 0.000), (471.152941176, 0.000), (473.188235294, 0.000), (475.223529412, 0.000), (477.258823529, 0.000), (479.294117647, 0.000), (481.329411765, 0.000), (483.364705882, 0.000), (485.4, 0.000), (487.435294118, 0.000), (489.470588235, 0.000), (491.505882353, 0.000), (493.541176471, 0.000), (495.576470588, 0.000), (497.611764706, 0.000), (499.647058824, 0.000), (501.682352941, 0.000), (503.717647059, 0.000), (505.752941176, 0.000), (507.788235294, 0.000), (509.823529412, 0.000), (511.858823529, 0.000), (513.894117647, 0.000), (515.929411765, 0.000), (517.964705882, 0.000), (520.0, 0.000) |  | Dimensionless |  |  |
| Cull_out_rate_adult_nanny | 1-HH_nanny_Consumption_rate{unitless} |  | Dimensionless |  |  |
| Cull_out_rate_Reserved_Adult_Ram | GRAPH(TIME) Points: (1.0, 0.000), (3.03529411765, 0.000), (5.07058823529, 0.000), (7.10588235294, 0.000), (9.14117647059, 0.000), (11.1764705882, 0.000), (13.2117647059, 0.000), (15.2470588235, 0.000), (17.2823529412, 0.000), (19.3176470588, 0.000), (21.3529411765, 0.000), (23.3882352941, 0.000), (25.4235294118, 0.000), (27.4588235294, 0.000), (29.4941176471, 0.000), (31.5294117647, 0.000), (33.5647058824, 0.000), (35.6, 0.000), (37.6352941176, 0.000), (39.6705882353, 0.000), (41.7058823529, 0.000), (43.7411764706, 0.000), (45.7764705882, 0.000), (47.8117647059, 0.000), (49.8470588235, 0.000), (51.8823529412, 0.000), (53.9176470588, 0.000), (55.9529411765, 0.000), (57.9882352941, 0.000), (60.0235294118, 0.000), (62.0588235294, 0.000), (64.0941176471, 0.000), (66.1294117647, 0.000), (68.1647058824, 0.000), (70.2, 0.000), (72.2352941176, 0.000), (74.2705882353, 0.000), (76.3058823529, 0.000), (78.3411764706, 0.000), (80.3764705882, 0.000), (82.4117647059, 0.000), (84.4470588235, 0.000), (86.4823529412, 0.000), (88.5176470588, 0.000), (90.5529411765, 0.000), (92.5882352941, 0.000), (94.6235294118, 0.000), (96.6588235294, 0.000), (98.6941176471, 0.000), (100.729411765, 0.000), (102.764705882, 0.000), (104.8, 0.000), (106.835294118, 0.000), (108.870588235, 0.000), (110.905882353, 0.000), (112.941176471, 0.000), (114.976470588, 0.000), (117.011764706, 0.000), (119.047058824, 0.000), (121.082352941, 0.000), (123.117647059, 0.000), (125.152941176, 0.000), (127.188235294, 0.000), (129.223529412, 0.000), (131.258823529, 0.000), (133.294117647, 0.000), (135.329411765, 0.000), (137.364705882, 0.000), (139.4, 0.000), (141.435294118, 0.000), (143.470588235, 0.000), (145.505882353, 0.000), (147.541176471, 0.000), (149.576470588, 0.000), (151.611764706, 1.000), (153.647058824, 0.000), (155.682352941, 1.000), (157.717647059, 0.000), (159.752941176, 0.000), (161.788235294, 0.000), (163.823529412, 0.000), (165.858823529, 0.000), (167.894117647, 0.000), (169.929411765, 0.000), (171.964705882, 0.000), (174.0, 0.000), (176.035294118, 0.000), (178.070588235, 0.000), (180.105882353, 0.000), (182.141176471, 0.000), (184.176470588, 0.000), (186.211764706, 0.000), (188.247058824, 0.000), (190.282352941, 0.000), (192.317647059, 0.000), (194.352941176, 0.000), (196.388235294, 0.000), (198.423529412, 0.000), (200.458823529, 0.000), (202.494117647, 0.000), (204.529411765, 0.000), (206.564705882, 0.000), (208.6, 0.000), (210.635294118, 0.000), (212.670588235, 0.000), (214.705882353, 0.000), (216.741176471, 0.000), (218.776470588, 0.000), (220.811764706, 0.000), (222.847058824, 0.000), (224.882352941, 0.000), (226.917647059, 0.000), (228.952941176, 0.000), (230.988235294, 0.000), (233.023529412, 0.000), (235.058823529, 0.000), (237.094117647, 0.000), (239.129411765, 0.000), (241.164705882, 0.000), (243.2, 0.000), (245.235294118, 0.000), (247.270588235, 0.000), (249.305882353, 0.000), (251.341176471, 0.000), (253.376470588, 0.000), (255.411764706, 0.000), (257.447058824, 0.000), (259.482352941, 0.000), (261.517647059, 0.000), (263.552941176, 0.000), (265.588235294, 0.000), (267.623529412, 0.000), (269.658823529, 0.000), (271.694117647, 0.000), (273.729411765, 0.000), (275.764705882, 0.000), (277.8, 0.000), (279.835294118, 0.000), (281.870588235, 0.000), (283.905882353, 0.000), (285.941176471, 0.000), (287.976470588, 0.000), (290.011764706, 0.000), (292.047058824, 0.000), (294.082352941, 0.000), (296.117647059, 0.000), (298.152941176, 0.000), (300.188235294, 0.000), (302.223529412, 1.000), (304.258823529, 0.000), (306.294117647, 0.000), (308.329411765, 0.000), (310.364705882, 0.000), (312.4, 0.000), (314.435294118, 0.000), (316.470588235, 0.000), (318.505882353, 0.000), (320.541176471, 0.000), (322.576470588, 0.000), (324.611764706, 0.000), (326.647058824, 0.000), (328.682352941, 0.000), (330.717647059, 0.000), (332.752941176, 0.000), (334.788235294, 0.000), (336.823529412, 0.000), (338.858823529, 0.000), (340.894117647, 0.000), (342.929411765, 0.000), (344.964705882, 0.000), (347.0, 0.000), (349.035294118, 0.000), (351.070588235, 0.000), (353.105882353, 0.000), (355.141176471, 0.000), (357.176470588, 0.000), (359.211764706, 0.000), (361.247058824, 0.000), (363.282352941, 0.000), (365.317647059, 0.000), (367.352941176, 0.000), (369.388235294, 0.000), (371.423529412, 0.000), (373.458823529, 0.000), (375.494117647, 0.000), (377.529411765, 0.000), (379.564705882, 0.000), (381.6, 0.000), (383.635294118, 0.000), (385.670588235, 0.000), (387.705882353, 0.000), (389.741176471, 0.000), (391.776470588, 0.000), (393.811764706, 0.000), (395.847058824, 0.000), (397.882352941, 0.000), (399.917647059, 0.000), (401.952941176, 0.000), (403.988235294, 0.000), (406.023529412, 0.000), (408.058823529, 0.000), (410.094117647, 0.000), (412.129411765, 0.000), (414.164705882, 0.000), (416.2, 0.000), (418.235294118, 0.000), (420.270588235, 0.000), (422.305882353, 0.000), (424.341176471, 0.000), (426.376470588, 0.000), (428.411764706, 0.000), (430.447058824, 0.000), (432.482352941, 0.000), (434.517647059, 0.000), (436.552941176, 0.000), (438.588235294, 0.000), (440.623529412, 0.000), (442.658823529, 0.000), (444.694117647, 0.000), (446.729411765, 0.000), (448.764705882, 0.000), (450.8, 1.000), (452.835294118, 0.000), (454.870588235, 0.000), (456.905882353, 0.000), (458.941176471, 0.000), (460.976470588, 0.000), (463.011764706, 0.000), (465.047058824, 0.000), (467.082352941, 0.000), (469.117647059, 0.000), (471.152941176, 0.000), (473.188235294, 0.000), (475.223529412, 0.000), (477.258823529, 0.000), (479.294117647, 0.000), (481.329411765, 0.000), (483.364705882, 0.000), (485.4, 0.000), (487.435294118, 0.000), (489.470588235, 0.000), (491.505882353, 0.000), (493.541176471, 0.000), (495.576470588, 0.000), (497.611764706, 0.000), (499.647058824, 0.000), (501.682352941, 0.000), (503.717647059, 0.000), (505.752941176, 0.000), (507.788235294, 0.000), (509.823529412, 0.000), (511.858823529, 0.000), (513.894117647, 0.000), (515.929411765, 0.000), (517.964705882, 0.000), (520.0, 0.000) |  | Dimensionless |  |  |
| Expected_production_investment[HHD_Gender, SR_activity_responsibility] | IF(SMTH1(Economic_Module.Profit, 1)>0) THEN 1 ELSE 0 |  | Dimensionless |  |  |
| Feeding_ration[HHD_Gender, SR_activity_responsibility] | IF(Expected_production_investment=1)THEN 2 ELSE 1 {unitless} |  | Dimensionless |  |  |
| Fertilization_success_goat[HHD_Gender, SR_activity_responsibility] | IF(((Adult_Buck_for_breeding*Bred_back_Nanny)>0)) THEN 1 ELSE 0 {unitless} |  | Dimensionless |  |  |
| Fertilization_success_sheep[HHD_Gender, SR_activity_responsibility] | IF((Adult_Ram_for_breeding>0) AND(Bred_back_Ewes>0)) THEN 1 ELSE 0 |  | Dimensionless |  |  |
| Fractional_abortion_rate_goat[HHD_Gender, SR_activity_responsibility] | 0.32*Animal_husbandry_effect_on_productivity {HH survey data} |  | Dimensionless |  |  |
| Fractional_abortion_rate_sheep[HHD_Gender, SR_activity_responsibility] | 0.52*Animal_husbandry_effect_on_productivity {HH survey data} |  | Dimensionless |  |  |
| Frequency_of_gifting | GRAPH(TIME) Points: (1.0, 0.000), (52.9, 0.000), (104.8, 0.000), (156.7, 0.000), (208.6, 1.000), (260.5, 0.000), (312.4, 0.000), (364.3, 0.000), (416.2, 0.000), (468.1, 0.000), (520.0, 1.000) |  | Dimensionless |  |  |
| Gestation_period_Goat | 37.5 {Lesnoff & Lancelot, 2010} |  | Weeks |  |  |
| Gestation_period_sheep | 37.5 {Lesnoff & Lancelot, 2010} |  | Weeks |  |  |
| Goat_Survival_rate[HHD_Gender, SR_activity_responsibility] | 1-Fractional_abortion_rate_goat {unitless} |  | Dimensionless |  |  |
| Grower_nanny_gifting_rate | 0.02 {unitless} |  | Dimensionless |  |  |
| HH_billy_Consumption_rate | GRAPH(TIME{HH survey data}) Points: (1.0, 0.0), (52.9, 0.02), (104.8, 0.02), (156.7, 0.02), (208.6, 0.02), (260.5, 0.02), (312.4, 0.02), (364.3, 0.02), (416.2, 0.02), (468.1, 0.02), (520.0, 0.02) |  | Dimensionless |  |  |
| HH_Ewe_Consumption_rate | GRAPH(TIME) Points: (1.0, 0.0), (52.9, 0.0), (104.8, 0.0), (156.7, 0.0), (208.6, 0.02), (260.5, 0.0), (312.4, 0.0), (364.3, 0.02), (416.2, 0.0), (468.1, 0.0), (520.0, 0.02) |  | Dimensionless |  |  |
| HH_nanny_Consumption_rate | GRAPH(TIME) Points: (1.0, 0.0), (52.9, 0.02), (104.8, 0.02), (156.7, 0.02), (208.6, 0.02), (260.5, 0.02), (312.4, 0.02), (364.3, 0.02), (416.2, 0.02), (468.1, 0.02), (520.0, 0.0) |  | Dimensionless |  |  |
| HH_nanny_consumption_time | 156{weeks} |  | Weeks |  |  |
| HH_Ram_Consumption_rate | GRAPH(TIME) Points: (1.0, 0.0), (52.9, 0.1), (104.8, 0.1), (156.7, 0.1), (208.6, 0.1), (260.5, 0.1), (312.4, 0.1), (364.3, 0.1), (416.2, 0.1), (468.1, 0.1), (520.0, 0.0) |  | Dimensionless |  |  |
| Infant_sheep_mortality_rate[HHD_Gender, SR_activity_responsibility] | IF(Bred_back_Ewes=0) THEN 0.6 ELSE 0 |  | Dimensionless |  |  |
| Kid_per_parturition | 1{No twinning assumed} |  | Per Week |  |  |
| Lamb_per_parturition | 2{Assumed no twinning} |  | Per Week |  |  |
| "Mortality_rate_>young_SR" | 0.6 {El Arbi et al., 2019 - for acute cases} |  | Per Week |  |  |
| Mortality_rate_young_SR | 0.6{El Arbi et al.,2019 for acute mortality rate} |  | Per Week |  |  |
| Number_stolen | GRAPH(TIME) Points: (1.0, 0.000), (52.9, 0.000), (104.8, 0.000), (156.7, 0.000), (208.6, 1.000), (260.5, 0.000), (312.4, 0.000), (364.3, 0.000), (416.2, 0.000), (468.1, 0.000), (520.0, 0.000) |  | Sheep per week |  |  |
| Number_stolen_goat | GRAPH(TIME {HH survey data}) Points: (1.0, 0.000), (52.9, 0.000), (104.8, 0.000), (156.7, 0.000), (208.6, 0.000), (260.5, 1.000), (312.4, 0.000), (364.3, 0.000), (416.2, 0.000), (468.1, 0.000), (520.0, 0.000) |  | Goat/Weeks |  |  |
| Offtake_Rate_Adult_billy | GRAPH(TIME {Sow et al., 2021}) Points: (1.0, 0.0), (3.03529411765, 0.0), (5.07058823529, 0.0), (7.10588235294, 0.0), (9.14117647059, 0.0), (11.1764705882, 0.0), (13.2117647059, 0.0), (15.2470588235, 0.0), (17.2823529412, 0.0), (19.3176470588, 0.0), (21.3529411765, 0.0), (23.3882352941, 0.0), (25.4235294118, 0.0), (27.4588235294, 0.0), (29.4941176471, 0.0), (31.5294117647, 0.0), (33.5647058824, 0.0), (35.6, 0.0), (37.6352941176, 0.0), (39.6705882353, 0.0), (41.7058823529, 0.0), (43.7411764706, 0.0), (45.7764705882, 0.0), (47.8117647059, 0.0), (49.8470588235, 0.0), (51.8823529412, 0.0), (53.9176470588, 0.0), (55.9529411765, 0.0), (57.9882352941, 0.0), (60.0235294118, 0.0), (62.0588235294, 0.0), (64.0941176471, 0.0), (66.1294117647, 0.0), (68.1647058824, 0.0), (70.2, 0.0), (72.2352941176, 0.0), (74.2705882353, 0.0), (76.3058823529, 0.0), (78.3411764706, 0.0), (80.3764705882, 0.0), (82.4117647059, 0.0), (84.4470588235, 0.0), (86.4823529412, 0.0), (88.5176470588, 0.0), (90.5529411765, 0.0), (92.5882352941, 0.0), (94.6235294118, 0.0), (96.6588235294, 0.0), (98.6941176471, 0.0), (100.729411765, 0.0), (102.764705882, 0.0), (104.8, 0.0), (106.835294118, 0.0), (108.870588235, 0.0), (110.905882353, 0.6), (112.941176471, 0.0), (114.976470588, 0.0), (117.011764706, 0.0), (119.047058824, 0.0), (121.082352941, 0.0), (123.117647059, 0.0), (125.152941176, 0.0), (127.188235294, 0.0), (129.223529412, 0.0), (131.258823529, 0.0), (133.294117647, 0.0), (135.329411765, 0.0), (137.364705882, 0.0), (139.4, 0.0), (141.435294118, 0.0), (143.470588235, 0.0), (145.505882353, 0.0), (147.541176471, 0.0), (149.576470588, 0.0), (151.611764706, 0.0), (153.647058824, 0.0), (155.682352941, 0.0), (157.717647059, 0.0), (159.752941176, 0.0), (161.788235294, 0.0), (163.823529412, 0.0), (165.858823529, 0.0), (167.894117647, 0.0), (169.929411765, 0.0), (171.964705882, 0.0), (174.0, 0.0), (176.035294118, 0.0), (178.070588235, 0.0), (180.105882353, 0.0), (182.141176471, 0.0), (184.176470588, 0.0), (186.211764706, 0.0), (188.247058824, 0.0), (190.282352941, 0.0), (192.317647059, 0.0), (194.352941176, 0.0), (196.388235294, 0.0), (198.423529412, 0.0), (200.458823529, 0.0), (202.494117647, 0.0), (204.529411765, 0.0), (206.564705882, 0.0), (208.6, 0.0), (210.635294118, 0.0), (212.670588235, 0.0), (214.705882353, 0.0), (216.741176471, 0.0), (218.776470588, 0.0), (220.811764706, 0.6), (222.847058824, 0.0), (224.882352941, 0.0), (226.917647059, 0.0), (228.952941176, 0.0), (230.988235294, 0.0), (233.023529412, 0.0), (235.058823529, 0.0), (237.094117647, 0.0), (239.129411765, 0.0), (241.164705882, 0.0), (243.2, 0.0), (245.235294118, 0.0), (247.270588235, 0.0), (249.305882353, 0.0), (251.341176471, 0.0), (253.376470588, 0.0), (255.411764706, 0.0), (257.447058824, 0.0), (259.482352941, 0.0), (261.517647059, 0.0), (263.552941176, 0.0), (265.588235294, 0.0), (267.623529412, 0.0), (269.658823529, 0.0), (271.694117647, 0.0), (273.729411765, 0.0), (275.764705882, 0.0), (277.8, 0.0), (279.835294118, 0.0), (281.870588235, 0.0), (283.905882353, 0.0), (285.941176471, 0.0), (287.976470588, 0.0), (290.011764706, 0.0), (292.047058824, 0.0), (294.082352941, 0.0), (296.117647059, 0.0), (298.152941176, 0.0), (300.188235294, 0.0), (302.223529412, 0.0), (304.258823529, 0.0), (306.294117647, 0.0), (308.329411765, 0.0), (310.364705882, 0.0), (312.4, 0.0), (314.435294118, 0.0), (316.470588235, 0.0), (318.505882353, 0.0), (320.541176471, 0.0), (322.576470588, 0.0), (324.611764706, 0.0), (326.647058824, 0.0), (328.682352941, 0.0), (330.717647059, 0.6), (332.752941176, 0.0), (334.788235294, 0.0), (336.823529412, 0.0), (338.858823529, 0.0), (340.894117647, 0.0), (342.929411765, 0.0), (344.964705882, 0.0), (347.0, 0.0), (349.035294118, 0.0), (351.070588235, 0.0), (353.105882353, 0.0), (355.141176471, 0.0), (357.176470588, 0.0), (359.211764706, 0.0), (361.247058824, 0.0), (363.282352941, 0.0), (365.317647059, 0.0), (367.352941176, 0.0), (369.388235294, 0.0), (371.423529412, 0.0), (373.458823529, 0.0), (375.494117647, 0.0), (377.529411765, 0.0), (379.564705882, 0.0), (381.6, 0.0), (383.635294118, 0.0), (385.670588235, 0.0), (387.705882353, 0.0), (389.741176471, 0.0), (391.776470588, 0.0), (393.811764706, 0.0), (395.847058824, 0.0), (397.882352941, 0.0), (399.917647059, 0.0), (401.952941176, 0.0), (403.988235294, 0.0), (406.023529412, 0.0), (408.058823529, 0.0), (410.094117647, 0.0), (412.129411765, 0.0), (414.164705882, 0.0), (416.2, 0.0), (418.235294118, 0.0), (420.270588235, 0.0), (422.305882353, 0.0), (424.341176471, 0.0), (426.376470588, 0.0), (428.411764706, 0.0), (430.447058824, 0.0), (432.482352941, 0.0), (434.517647059, 0.0), (436.552941176, 0.0), (438.588235294, 0.0), (440.623529412, 0.6), (442.658823529, 0.0), (444.694117647, 0.0), (446.729411765, 0.0), (448.764705882, 0.0), (450.8, 0.0), (452.835294118, 0.0), (454.870588235, 0.0), (456.905882353, 0.0), (458.941176471, 0.0), (460.976470588, 0.0), (463.011764706, 0.0), (465.047058824, 0.0), (467.082352941, 0.0), (469.117647059, 0.0), (471.152941176, 0.0), (473.188235294, 0.0), (475.223529412, 0.0), (477.258823529, 0.0), (479.294117647, 0.0), (481.329411765, 0.0), (483.364705882, 0.0), (485.4, 0.0), (487.435294118, 0.0), (489.470588235, 0.0), (491.505882353, 0.0), (493.541176471, 0.0), (495.576470588, 0.0), (497.611764706, 0.0), (499.647058824, 0.0), (501.682352941, 0.0), (503.717647059, 0.0), (505.752941176, 0.0), (507.788235294, 0.0), (509.823529412, 0.0), (511.858823529, 0.0), (513.894117647, 0.0), (515.929411765, 0.0), (517.964705882, 0.0), (520.0, 0.0) |  | Dimensionless |  |  |
| Offtake_Rate_Adult_Ram | GRAPH(TIME {after 120 weeks -Sow et al., 2021}) Points: (1.0, 0.0), (3.03529411765, 0.0), (5.07058823529, 0.0), (7.10588235294, 0.0), (9.14117647059, 0.0), (11.1764705882, 0.0), (13.2117647059, 0.0), (15.2470588235, 0.0), (17.2823529412, 0.0), (19.3176470588, 0.0), (21.3529411765, 0.0), (23.3882352941, 0.0), (25.4235294118, 0.0), (27.4588235294, 0.0), (29.4941176471, 0.0), (31.5294117647, 0.0), (33.5647058824, 0.0), (35.6, 0.0), (37.6352941176, 0.0), (39.6705882353, 0.0), (41.7058823529, 0.0), (43.7411764706, 0.0), (45.7764705882, 0.0), (47.8117647059, 0.0), (49.8470588235, 0.0), (51.8823529412, 0.0), (53.9176470588, 0.0), (55.9529411765, 0.0), (57.9882352941, 0.0), (60.0235294118, 0.0), (62.0588235294, 0.0), (64.0941176471, 0.0), (66.1294117647, 0.0), (68.1647058824, 0.0), (70.2, 0.0), (72.2352941176, 0.0), (74.2705882353, 0.0), (76.3058823529, 0.0), (78.3411764706, 0.0), (80.3764705882, 0.0), (82.4117647059, 0.0), (84.4470588235, 0.0), (86.4823529412, 0.0), (88.5176470588, 0.0), (90.5529411765, 0.0), (92.5882352941, 0.0), (94.6235294118, 0.0), (96.6588235294, 0.0), (98.6941176471, 0.0), (100.729411765, 0.0), (102.764705882, 0.0), (104.8, 0.0), (106.835294118, 0.0), (108.870588235, 0.0), (110.905882353, 0.0), (112.941176471, 0.0), (114.976470588, 0.0), (117.011764706, 0.0), (119.047058824, 0.0), (121.082352941, 0.6), (123.117647059, 0.0), (125.152941176, 0.0), (127.188235294, 0.0), (129.223529412, 0.0), (131.258823529, 0.0), (133.294117647, 0.0), (135.329411765, 0.0), (137.364705882, 0.0), (139.4, 0.0), (141.435294118, 0.0), (143.470588235, 0.0), (145.505882353, 0.0), (147.541176471, 0.0), (149.576470588, 0.0), (151.611764706, 0.0), (153.647058824, 0.0), (155.682352941, 0.0), (157.717647059, 0.0), (159.752941176, 0.0), (161.788235294, 0.0), (163.823529412, 0.0), (165.858823529, 0.0), (167.894117647, 0.0), (169.929411765, 0.0), (171.964705882, 0.0), (174.0, 0.0), (176.035294118, 0.0), (178.070588235, 0.0), (180.105882353, 0.0), (182.141176471, 0.0), (184.176470588, 0.0), (186.211764706, 0.0), (188.247058824, 0.0), (190.282352941, 0.0), (192.317647059, 0.0), (194.352941176, 0.0), (196.388235294, 0.0), (198.423529412, 0.0), (200.458823529, 0.0), (202.494117647, 0.0), (204.529411765, 0.0), (206.564705882, 0.0), (208.6, 0.0), (210.635294118, 0.0), (212.670588235, 0.0), (214.705882353, 0.0), (216.741176471, 0.0), (218.776470588, 0.0), (220.811764706, 0.0), (222.847058824, 0.0), (224.882352941, 0.0), (226.917647059, 0.0), (228.952941176, 0.0), (230.988235294, 0.0), (233.023529412, 0.0), (235.058823529, 0.0), (237.094117647, 0.0), (239.129411765, 0.0), (241.164705882, 0.6), (243.2, 0.0), (245.235294118, 0.0), (247.270588235, 0.0), (249.305882353, 0.0), (251.341176471, 0.0), (253.376470588, 0.0), (255.411764706, 0.0), (257.447058824, 0.0), (259.482352941, 0.0), (261.517647059, 0.0), (263.552941176, 0.0), (265.588235294, 0.0), (267.623529412, 0.0), (269.658823529, 0.0), (271.694117647, 0.0), (273.729411765, 0.0), (275.764705882, 0.0), (277.8, 0.0), (279.835294118, 0.0), (281.870588235, 0.0), (283.905882353, 0.0), (285.941176471, 0.0), (287.976470588, 0.0), (290.011764706, 0.0), (292.047058824, 0.0), (294.082352941, 0.0), (296.117647059, 0.0), (298.152941176, 0.0), (300.188235294, 0.0), (302.223529412, 0.0), (304.258823529, 0.0), (306.294117647, 0.0), (308.329411765, 0.0), (310.364705882, 0.0), (312.4, 0.0), (314.435294118, 0.0), (316.470588235, 0.0), (318.505882353, 0.0), (320.541176471, 0.0), (322.576470588, 0.0), (324.611764706, 0.0), (326.647058824, 0.0), (328.682352941, 0.0), (330.717647059, 0.0), (332.752941176, 0.0), (334.788235294, 0.0), (336.823529412, 0.0), (338.858823529, 0.0), (340.894117647, 0.0), (342.929411765, 0.0), (344.964705882, 0.0), (347.0, 0.0), (349.035294118, 0.0), (351.070588235, 0.0), (353.105882353, 0.0), (355.141176471, 0.0), (357.176470588, 0.0), (359.211764706, 0.0), (361.247058824, 0.6), (363.282352941, 0.0), (365.317647059, 0.0), (367.352941176, 0.0), (369.388235294, 0.0), (371.423529412, 0.0), (373.458823529, 0.0), (375.494117647, 0.0), (377.529411765, 0.0), (379.564705882, 0.0), (381.6, 0.0), (383.635294118, 0.0), (385.670588235, 0.0), (387.705882353, 0.0), (389.741176471, 0.0), (391.776470588, 0.0), (393.811764706, 0.0), (395.847058824, 0.0), (397.882352941, 0.0), (399.917647059, 0.0), (401.952941176, 0.0), (403.988235294, 0.0), (406.023529412, 0.0), (408.058823529, 0.0), (410.094117647, 0.0), (412.129411765, 0.0), (414.164705882, 0.0), (416.2, 0.0), (418.235294118, 0.0), (420.270588235, 0.0), (422.305882353, 0.0), (424.341176471, 0.0), (426.376470588, 0.0), (428.411764706, 0.0), (430.447058824, 0.0), (432.482352941, 0.0), (434.517647059, 0.0), (436.552941176, 0.0), (438.588235294, 0.0), (440.623529412, 0.0), (442.658823529, 0.0), (444.694117647, 0.0), (446.729411765, 0.0), (448.764705882, 0.0), (450.8, 0.0), (452.835294118, 0.0), (454.870588235, 0.0), (456.905882353, 0.0), (458.941176471, 0.0), (460.976470588, 0.0), (463.011764706, 0.0), (465.047058824, 0.0), (467.082352941, 0.0), (469.117647059, 0.0), (471.152941176, 0.0), (473.188235294, 0.0), (475.223529412, 0.0), (477.258823529, 0.0), (479.294117647, 0.0), (481.329411765, 0.6), (483.364705882, 0.0), (485.4, 0.0), (487.435294118, 0.0), (489.470588235, 0.0), (491.505882353, 0.0), (493.541176471, 0.0), (495.576470588, 0.0), (497.611764706, 0.0), (499.647058824, 0.0), (501.682352941, 0.0), (503.717647059, 0.0), (505.752941176, 0.0), (507.788235294, 0.0), (509.823529412, 0.0), (511.858823529, 0.0), (513.894117647, 0.0), (515.929411765, 0.0), (517.964705882, 0.0), (520.0, 0.0) |  | Dimensionless |  |  |
| Offtake_rate_reserved_adult_billy | GRAPH(TIME {Sow et al., 2021}) Points: (1.0, 0.0), (3.03529411765, 0.0), (5.07058823529, 0.0), (7.10588235294, 0.0), (9.14117647059, 0.0), (11.1764705882, 0.0), (13.2117647059, 0.0), (15.2470588235, 0.0), (17.2823529412, 0.0), (19.3176470588, 0.0), (21.3529411765, 0.0), (23.3882352941, 0.0), (25.4235294118, 0.0), (27.4588235294, 0.0), (29.4941176471, 0.0), (31.5294117647, 0.0), (33.5647058824, 0.0), (35.6, 0.0), (37.6352941176, 0.0), (39.6705882353, 0.0), (41.7058823529, 0.0), (43.7411764706, 0.0), (45.7764705882, 0.0), (47.8117647059, 0.0), (49.8470588235, 0.0), (51.8823529412, 0.0), (53.9176470588, 0.0), (55.9529411765, 0.0), (57.9882352941, 0.0), (60.0235294118, 0.0), (62.0588235294, 0.0), (64.0941176471, 0.0), (66.1294117647, 0.0), (68.1647058824, 0.0), (70.2, 0.0), (72.2352941176, 0.0), (74.2705882353, 0.0), (76.3058823529, 0.0), (78.3411764706, 0.0), (80.3764705882, 0.0), (82.4117647059, 0.0), (84.4470588235, 0.0), (86.4823529412, 0.0), (88.5176470588, 0.0), (90.5529411765, 0.0), (92.5882352941, 0.0), (94.6235294118, 0.0), (96.6588235294, 0.0), (98.6941176471, 0.0), (100.729411765, 0.0), (102.764705882, 0.0), (104.8, 0.0), (106.835294118, 0.0), (108.870588235, 0.0), (110.905882353, 0.0), (112.941176471, 0.0), (114.976470588, 0.0), (117.011764706, 0.0), (119.047058824, 0.0), (121.082352941, 0.0), (123.117647059, 0.0), (125.152941176, 0.0), (127.188235294, 0.0), (129.223529412, 0.0), (131.258823529, 0.0), (133.294117647, 0.0), (135.329411765, 0.0), (137.364705882, 0.0), (139.4, 0.0), (141.435294118, 0.0), (143.470588235, 0.0), (145.505882353, 0.0), (147.541176471, 0.0), (149.576470588, 0.0), (151.611764706, 0.0), (153.647058824, 0.0), (155.682352941, 0.0), (157.717647059, 0.0), (159.752941176, 0.0), (161.788235294, 0.0), (163.823529412, 0.0), (165.858823529, 0.0), (167.894117647, 0.0), (169.929411765, 0.0), (171.964705882, 0.0), (174.0, 0.0), (176.035294118, 0.0), (178.070588235, 0.0), (180.105882353, 0.0), (182.141176471, 0.0), (184.176470588, 0.0), (186.211764706, 0.0), (188.247058824, 0.0), (190.282352941, 0.0), (192.317647059, 0.0), (194.352941176, 0.0), (196.388235294, 0.0), (198.423529412, 0.0), (200.458823529, 0.0), (202.494117647, 0.0), (204.529411765, 0.0), (206.564705882, 0.0), (208.6, 0.0), (210.635294118, 0.0), (212.670588235, 0.0), (214.705882353, 0.0), (216.741176471, 0.0), (218.776470588, 0.0), (220.811764706, 0.0), (222.847058824, 0.0), (224.882352941, 0.0), (226.917647059, 0.0), (228.952941176, 0.0), (230.988235294, 0.0), (233.023529412, 0.0), (235.058823529, 0.0), (237.094117647, 0.0), (239.129411765, 0.0), (241.164705882, 0.0), (243.2, 0.0), (245.235294118, 0.0), (247.270588235, 0.0), (249.305882353, 0.0), (251.341176471, 0.0), (253.376470588, 0.0), (255.411764706, 0.0), (257.447058824, 0.0), (259.482352941, 0.0), (261.517647059, 0.0), (263.552941176, 0.0), (265.588235294, 0.0), (267.623529412, 0.0), (269.658823529, 0.0), (271.694117647, 0.0), (273.729411765, 0.0), (275.764705882, 0.0), (277.8, 0.0), (279.835294118, 0.0), (281.870588235, 0.0), (283.905882353, 0.0), (285.941176471, 0.0), (287.976470588, 0.0), (290.011764706, 0.0), (292.047058824, 0.0), (294.082352941, 0.0), (296.117647059, 0.0), (298.152941176, 0.0), (300.188235294, 0.0), (302.223529412, 0.0), (304.258823529, 0.0), (306.294117647, 0.0), (308.329411765, 0.0), (310.364705882, 0.0), (312.4, 0.0), (314.435294118, 0.0), (316.470588235, 0.0), (318.505882353, 0.0), (320.541176471, 0.0), (322.576470588, 0.0), (324.611764706, 0.0), (326.647058824, 0.0), (328.682352941, 0.0), (330.717647059, 0.0), (332.752941176, 0.0), (334.788235294, 0.0), (336.823529412, 0.0), (338.858823529, 0.0), (340.894117647, 0.0), (342.929411765, 0.0), (344.964705882, 0.0), (347.0, 0.0), (349.035294118, 0.0), (351.070588235, 0.0), (353.105882353, 0.0), (355.141176471, 0.0), (357.176470588, 0.0), (359.211764706, 0.0), (361.247058824, 0.0), (363.282352941, 0.0), (365.317647059, 0.0), (367.352941176, 0.0), (369.388235294, 0.0), (371.423529412, 0.0), (373.458823529, 0.0), (375.494117647, 0.0), (377.529411765, 0.0), (379.564705882, 0.0), (381.6, 0.0), (383.635294118, 0.0), (385.670588235, 0.0), (387.705882353, 0.0), (389.741176471, 0.0), (391.776470588, 0.0), (393.811764706, 0.0), (395.847058824, 0.0), (397.882352941, 0.0), (399.917647059, 0.0), (401.952941176, 0.0), (403.988235294, 0.0), (406.023529412, 0.0), (408.058823529, 0.0), (410.094117647, 0.0), (412.129411765, 0.0), (414.164705882, 0.0), (416.2, 1.0), (418.235294118, 0.0), (420.270588235, 0.0), (422.305882353, 0.0), (424.341176471, 0.0), (426.376470588, 0.0), (428.411764706, 0.0), (430.447058824, 0.0), (432.482352941, 0.0), (434.517647059, 0.0), (436.552941176, 0.0), (438.588235294, 0.0), (440.623529412, 0.0), (442.658823529, 0.0), (444.694117647, 0.0), (446.729411765, 0.0), (448.764705882, 0.0), (450.8, 0.0), (452.835294118, 0.0), (454.870588235, 0.0), (456.905882353, 0.0), (458.941176471, 0.0), (460.976470588, 0.0), (463.011764706, 0.0), (465.047058824, 0.0), (467.082352941, 0.0), (469.117647059, 0.0), (471.152941176, 0.0), (473.188235294, 0.0), (475.223529412, 0.0), (477.258823529, 0.0), (479.294117647, 0.0), (481.329411765, 0.0), (483.364705882, 0.0), (485.4, 0.0), (487.435294118, 0.0), (489.470588235, 0.0), (491.505882353, 0.0), (493.541176471, 0.0), (495.576470588, 0.0), (497.611764706, 0.0), (499.647058824, 0.0), (501.682352941, 0.0), (503.717647059, 0.0), (505.752941176, 0.0), (507.788235294, 0.0), (509.823529412, 0.0), (511.858823529, 0.0), (513.894117647, 0.0), (515.929411765, 0.0), (517.964705882, 0.0), (520.0, 0.0) |  | Dimensionless |  |  |
| Offtake_rate_Young_Ewe | 0.2 {HH survey data} |  | Dimensionless |  |  |
| Offtake_rate_Young_nanny | GRAPH(TIME) Points: (1.0, 0.0), (3.03529411765, 0.0), (5.07058823529, 0.0), (7.10588235294, 0.0), (9.14117647059, 0.0), (11.1764705882, 0.0), (13.2117647059, 0.0), (15.2470588235, 0.0), (17.2823529412, 0.0), (19.3176470588, 0.0), (21.3529411765, 0.0), (23.3882352941, 0.0), (25.4235294118, 0.0), (27.4588235294, 0.02), (29.4941176471, 0.0), (31.5294117647, 0.0), (33.5647058824, 0.0), (35.6, 0.0), (37.6352941176, 0.0), (39.6705882353, 0.0), (41.7058823529, 0.0), (43.7411764706, 0.0), (45.7764705882, 0.0), (47.8117647059, 0.0), (49.8470588235, 0.0), (51.8823529412, 0.0), (53.9176470588, 0.0), (55.9529411765, 0.0), (57.9882352941, 0.0), (60.0235294118, 0.0), (62.0588235294, 0.0), (64.0941176471, 0.0), (66.1294117647, 0.0), (68.1647058824, 0.0), (70.2, 0.0), (72.2352941176, 0.0), (74.2705882353, 0.0), (76.3058823529, 0.0), (78.3411764706, 0.02), (80.3764705882, 0.0), (82.4117647059, 0.0), (84.4470588235, 0.0), (86.4823529412, 0.0), (88.5176470588, 0.0), (90.5529411765, 0.0), (92.5882352941, 0.0), (94.6235294118, 0.0), (96.6588235294, 0.0), (98.6941176471, 0.0), (100.729411765, 0.0), (102.764705882, 0.0), (104.8, 0.0), (106.835294118, 0.0), (108.870588235, 0.0), (110.905882353, 0.0), (112.941176471, 0.0), (114.976470588, 0.0), (117.011764706, 0.0), (119.047058824, 0.0), (121.082352941, 0.0), (123.117647059, 0.0), (125.152941176, 0.0), (127.188235294, 0.0), (129.223529412, 0.0), (131.258823529, 0.02), (133.294117647, 0.0), (135.329411765, 0.0), (137.364705882, 0.0), (139.4, 0.0), (141.435294118, 0.0), (143.470588235, 0.0), (145.505882353, 0.0), (147.541176471, 0.0), (149.576470588, 0.0), (151.611764706, 0.0), (153.647058824, 0.0), (155.682352941, 0.0), (157.717647059, 0.0), (159.752941176, 0.0), (161.788235294, 0.0), (163.823529412, 0.0), (165.858823529, 0.0), (167.894117647, 0.0), (169.929411765, 0.0), (171.964705882, 0.0), (174.0, 0.0), (176.035294118, 0.0), (178.070588235, 0.0), (180.105882353, 0.0), (182.141176471, 0.0), (184.176470588, 0.02), (186.211764706, 0.0), (188.247058824, 0.0), (190.282352941, 0.0), (192.317647059, 0.0), (194.352941176, 0.0), (196.388235294, 0.0), (198.423529412, 0.0), (200.458823529, 0.0), (202.494117647, 0.0), (204.529411765, 0.0), (206.564705882, 0.0), (208.6, 0.0), (210.635294118, 0.0), (212.670588235, 0.0), (214.705882353, 0.0), (216.741176471, 0.0), (218.776470588, 0.0), (220.811764706, 0.0), (222.847058824, 0.0), (224.882352941, 0.0), (226.917647059, 0.0), (228.952941176, 0.0), (230.988235294, 0.0), (233.023529412, 0.0), (235.058823529, 0.02), (237.094117647, 0.0), (239.129411765, 0.0), (241.164705882, 0.0), (243.2, 0.0), (245.235294118, 0.0), (247.270588235, 0.0), (249.305882353, 0.0), (251.341176471, 0.0), (253.376470588, 0.0), (255.411764706, 0.0), (257.447058824, 0.0), (259.482352941, 0.0), (261.517647059, 0.0), (263.552941176, 0.0), (265.588235294, 0.0), (267.623529412, 0.0), (269.658823529, 0.0), (271.694117647, 0.0), (273.729411765, 0.0), (275.764705882, 0.0), (277.8, 0.0), (279.835294118, 0.0), (281.870588235, 0.0), (283.905882353, 0.0), (285.941176471, 0.0), (287.976470588, 0.02), (290.011764706, 0.0), (292.047058824, 0.0), (294.082352941, 0.0), (296.117647059, 0.0), (298.152941176, 0.0), (300.188235294, 0.0), (302.223529412, 0.0), (304.258823529, 0.0), (306.294117647, 0.0), (308.329411765, 0.0), (310.364705882, 0.0), (312.4, 0.0), (314.435294118, 0.0), (316.470588235, 0.0), (318.505882353, 0.0), (320.541176471, 0.0), (322.576470588, 0.0), (324.611764706, 0.0), (326.647058824, 0.0), (328.682352941, 0.0), (330.717647059, 0.0), (332.752941176, 0.0), (334.788235294, 0.0), (336.823529412, 0.0), (338.858823529, 0.02), (340.894117647, 0.0), (342.929411765, 0.0), (344.964705882, 0.0), (347.0, 0.0), (349.035294118, 0.0), (351.070588235, 0.0), (353.105882353, 0.0), (355.141176471, 0.0), (357.176470588, 0.0), (359.211764706, 0.0), (361.247058824, 0.0), (363.282352941, 0.0), (365.317647059, 0.0), (367.352941176, 0.0), (369.388235294, 0.0), (371.423529412, 0.0), (373.458823529, 0.0), (375.494117647, 0.0), (377.529411765, 0.0), (379.564705882, 0.0), (381.6, 0.0), (383.635294118, 0.0), (385.670588235, 0.0), (387.705882353, 0.0), (389.741176471, 0.0), (391.776470588, 0.02), (393.811764706, 0.0), (395.847058824, 0.0), (397.882352941, 0.0), (399.917647059, 0.0), (401.952941176, 0.0), (403.988235294, 0.0), (406.023529412, 0.0), (408.058823529, 0.0), (410.094117647, 0.0), (412.129411765, 0.0), (414.164705882, 0.0), (416.2, 0.0), (418.235294118, 0.0), (420.270588235, 0.0), (422.305882353, 0.0), (424.341176471, 0.0), (426.376470588, 0.0), (428.411764706, 0.0), (430.447058824, 0.0), (432.482352941, 0.0), (434.517647059, 0.0), (436.552941176, 0.0), (438.588235294, 0.0), (440.623529412, 0.0), (442.658823529, 0.0), (444.694117647, 0.02), (446.729411765, 0.0), (448.764705882, 0.0), (450.8, 0.0), (452.835294118, 0.0), (454.870588235, 0.0), (456.905882353, 0.0), (458.941176471, 0.0), (460.976470588, 0.0), (463.011764706, 0.0), (465.047058824, 0.0), (467.082352941, 0.0), (469.117647059, 0.0), (471.152941176, 0.0), (473.188235294, 0.0), (475.223529412, 0.0), (477.258823529, 0.0), (479.294117647, 0.0), (481.329411765, 0.0), (483.364705882, 0.0), (485.4, 0.0), (487.435294118, 0.0), (489.470588235, 0.0), (491.505882353, 0.0), (493.541176471, 0.0), (495.576470588, 0.02), (497.611764706, 0.0), (499.647058824, 0.0), (501.682352941, 0.0), (503.717647059, 0.0), (505.752941176, 0.0), (507.788235294, 0.0), (509.823529412, 0.0), (511.858823529, 0.0), (513.894117647, 0.0), (515.929411765, 0.0), (517.964705882, 0.0), (520.0, 0.0) |  | Dimensionless |  |  |
| Percentage_of_nannies_reserved_for_breeding_annually | 1 {per week} |  | Per Week |  |  |
| Proportion_of_vaccinated_grower_ram_given_out | 0.2*Young_ram_gifting_rate |  | Dimensionless |  |  |
| Proportion_sold_young_ram_3_to_6_months_natural_recovered | 0.5*Young_ram_selling_'rate |  | Dimensionless |  |  |
| Proportion_wrongly_identified | 0{assumed} |  | Dimensionless |  |  |
| Ram_reserve_breeding_rate | Offtake_Rate_Adult_Ram*HH_Ram_Consumption_rate {unitless} |  | Dimensionless |  |  |
| Ram_to_Ewe_ratio | 0.5 {HH survey data} |  | Dimensionless |  |  |
| Required_feed_ration | 2 {unitless} |  | Dimensionless |  |  |
| "Seroprevalence_Adult_goat_>12_months" | 0.25 {ElArbi et al., 2019} |  | Per Week |  |  |
| "Seroprevalence_Adult_sheep_>12_months" | 0.25 {ElArbi et al., 2019} |  | Per Week |  |  |
| Seroprevalence_Grower_goat_6_to_12_months | 0.25 {ElArbi et al., 2019} |  | Per Week |  |  |
| Seroprevalence_Grower_sheep_6_to_12_months | 0.25{ElArbi et al., 2019} |  | Per Week |  |  |
| Seroprevalence_young_goat_3_to_6_months | 0.25{ElArbi et al., 2019} |  | Per Week |  |  |
| Seroprevalence_Young_sheep_3_to_6_months | 0.25{ElArbi et al., 2019 } |  | Per Week |  |  |
| Sheep_Survival_rate[HHD_Gender, SR_activity_responsibility] | 1-Fractional_abortion_rate_sheep |  | Dimensionless |  |  |
| Time_being_kid | 16{weeks} |  | Weeks |  |  |
| Time_being_lamb | 12 |  | Weeks |  |  |
| Time_ewe_kept | TRIANGULAR(77.9, 102, 135) {Sow et al., 2021} |  | Weeks |  |  |
| Time_for_goat_to_become_adult | 52{weeks} |  | Weeks |  |  |
| Time_for_goat_to_grow | 26 |  | Weeks |  |  |
| Time_for_HH_ewe_slaughter | 1 |  | Weeks |  |  |
| Time_for_ram_to_start_breeding | 52{week} |  | Weeks |  |  |
| Time_for_sheep_to_become_Adult | 52 |  | Weeks |  |  |
| Time_for_sheep_to_grow | 26 {weeks} |  | Weeks |  |  |
| Time_HH_slaughter | 1 |  | Weeks |  |  |
| Time_of_HH_Ram_Consumption | 1 |  | Weeks |  |  |
| Time_to_gift_goat | 1 |  | Weeks |  |  |
| Time_to_gift_sheep | 1{HH survey data} |  | Weeks |  |  |
| Time_to_receive_Adult_ram | 1 |  | Weeks |  |  |
| Time_to_receive_young_sheep | 1 |  | Weeks |  |  |
| Time_to_sell_adult_billy | 1 |  | Weeks |  |  |
| Time_to_sell_adult_ewe | TRIANGULAR(77.9, 102, 135) {Sow et al., 2021} |  | Weeks |  |  |
| Time_to_sell_adult_ram | 1 |  | Weeks |  |  |
| Time_to_sell_bred_back_nanny | TRIANGULAR(311.6, 408, 540) {sow et al., 2021} |  | Weeks |  |  |
| Time_to_sell_reserved_billy_for_breeding | 1 |  | Weeks |  |  |
| Time_to_sell_reserved_ram_for_breeding | 1 |  | Weeks |  |  |
| Time_to_sell_young_billy | 1 |  | Weeks |  |  |
| Time_to_sell_young_lamb | 26{Household survey data} |  | Weeks |  |  |
| Time_to_wean_young_goat | 13{Weeks} |  | Weeks |  |  |
| Time_to_wean_young_lamb | 13{https://agriculture.vic.gov.au/support-and-resources/newsletters/sheep-notes-newsletter/winter-2019/early-weaning#:~:text=Whilst%20the%20recommended%20lamb%20weaning,get%20them%20on%20a%20ration} |  | Weeks |  |  |
| "Total_Adult_>12_months"[HHD_Gender, SR_activity_responsibility] | "Adult_Ram_>12_months_susceptible"+"Adult_Ram_>12_months_Recovered"+"Adult_Ram_>_12_months_infected"+"Adult_Ram_>_12_months_vaccinated"+"Adult_Ewe_>12_months_recovered"+Bred_back_Ewes+"Adult_Ewe_>12_months"+"Adult_Ewe_>12_months_infected"+"Adult_Ewe_>_12_months_susceptible" |  | Sheep |  |  |
| "Total_Adult_goat_>12_months"[HHD_Gender, SR_activity_responsibility] | "Adult_billy_>_12_months_susceptible"+"Adult_billy_>12_months_infected"+"Adult_billy_>12_months_recovered"+"Adult_billy_>12_months_vaccinated"+Adult_Buck_for_breeding+"Adult_nanny_>_12_months_susceptible"+"Adult_nanny_>12_months_infected"+"Adult_nanny_>12_months_vaccinated"+"Adult_nanny_>12_months_recovered"+Bred_back_Nanny |  | Goat |  |  |
| Total_goat[HHD_Gender, SR_activity_responsibility] | Total_young_goat_3_to_6_months+Total_Grower_goat_6_to_12_months+"Total_Adult_goat_>12_months" |  | Goat |  |  |
| Total_Grower_goat_6_to_12_months[HHD_Gender, SR_activity_responsibility] | Grower_billy_6_to_12_months_susceptible+Grower_billy_6_to_12_months_recovered+Grower_billy_6_to_12_months_infected+Grower_billy_6_to_12_months_vaccinated_recovered+Grower_billy_6_to_12_months_vaccinated+Grower_nanny_6_to_12_months_susceptible+Grower_nanny_6_to_12_months_infected+Grower_nanny_6_to_12_months_vaccinated_recovered+Grower_nanny_6_to_12_months_recovered+Grower_nanny_6_to_12_months_vaccinated |  | Goat |  |  |
| Total_Grower_sheep_6_to_12_months[HHD_Gender, SR_activity_responsibility] | Grower_Rams_6_to_12_months_natural_recovered+Grower_Rams_6_to_12_months_Susceptible+Grower_Rams_6_to_12_months_infected+Grower_Rams_6_to_12_months_vaccinated+Grower_Rams_6_to_12_months+Grower_Ewe_6_to_12_months_natural_recovered+Grower_Ewe_6_to_12_months+Grower_Ewe_6_to_12_months_infected+Grower_Ewe_6_to_12_months_vaccinated+Grower_Ewe_6_to_12_months_susceptible |  | Sheep |  |  |
| Total_sheep[HHD_Gender, SR_activity_responsibility] | Total_Young_sheep_3_to_6_months+Total_Grower_sheep_6_to_12_months+"Total_Adult_>12_months" |  | Sheep |  |  |
| Total_young_goat_3_to_6_months[HHD_Gender, SR_activity_responsibility] | Young_billy_3_to_6_months_susceptible+Young_billy_3_to_6_months_vaccinated+Young_billy_3_to_6_months_infected+Young_billy_3_to_6_months_recovered+Young_billy_3_to_6_months_vaccinated_recovered+Young_nanny_3_to_6_months_susceptible+Young_nanny_3_to_6_months_infected+Young_nanny_3_to_6_months_recovered+Young_nanny_3_to_6_months_vaccinated+Young_nanny_3_to_6_months_vaccinated_recovered |  | Goat |  |  |
| Total_Young_sheep_3_to_6_months[HHD_Gender, SR_activity_responsibility] | Young_Ram_3_to_6_months_infected+Young_Ram_3_to_6_months_susceptible+Young_Ram_3_to_6_months_vaccinated+Young_Ram_3_to_6_months_natural_recovered+Young_Ram_3_to_6_months_vaccinated_recovered+Young_Ewe_3_to_6_months_vaccinated_recovered+Young_Ewe_3_to_6_months_natural_recovered+Young_Ewe_3_to_6_months_infected+Young_Ewe_3_to_6_months_vaccinated+Young_Ewe_3_to_6_months_susceptible |  | Sheep |  |  |
| vaccine_recovery_duration | 1 |  | Weeks |  |  |
| Young_billy_gifting_rate | 0.02 |  | Dimensionless |  |  |
| Young_billy_selling_'rate | GRAPH(TIME) Points: (1.0, 0.0), (3.03529411765, 0.0), (5.07058823529, 0.0), (7.10588235294, 0.0), (9.14117647059, 0.0), (11.1764705882, 0.0), (13.2117647059, 0.0), (15.2470588235, 0.0), (17.2823529412, 0.0), (19.3176470588, 0.0), (21.3529411765, 0.0), (23.3882352941, 0.0), (25.4235294118, 0.0), (27.4588235294, 0.01), (29.4941176471, 0.0), (31.5294117647, 0.0), (33.5647058824, 0.0), (35.6, 0.0), (37.6352941176, 0.0), (39.6705882353, 0.0), (41.7058823529, 0.0), (43.7411764706, 0.0), (45.7764705882, 0.0), (47.8117647059, 0.0), (49.8470588235, 0.0), (51.8823529412, 0.0), (53.9176470588, 0.0), (55.9529411765, 0.0), (57.9882352941, 0.0), (60.0235294118, 0.0), (62.0588235294, 0.0), (64.0941176471, 0.0), (66.1294117647, 0.0), (68.1647058824, 0.0), (70.2, 0.0), (72.2352941176, 0.0), (74.2705882353, 0.0), (76.3058823529, 0.0), (78.3411764706, 0.1), (80.3764705882, 0.0), (82.4117647059, 0.0), (84.4470588235, 0.0), (86.4823529412, 0.0), (88.5176470588, 0.0), (90.5529411765, 0.0), (92.5882352941, 0.0), (94.6235294118, 0.0), (96.6588235294, 0.0), (98.6941176471, 0.0), (100.729411765, 0.0), (102.764705882, 0.0), (104.8, 0.0), (106.835294118, 0.0), (108.870588235, 0.0), (110.905882353, 0.0), (112.941176471, 0.0), (114.976470588, 0.0), (117.011764706, 0.0), (119.047058824, 0.0), (121.082352941, 0.0), (123.117647059, 0.0), (125.152941176, 0.0), (127.188235294, 0.0), (129.223529412, 0.0), (131.258823529, 0.01), (133.294117647, 0.0), (135.329411765, 0.0), (137.364705882, 0.0), (139.4, 0.0), (141.435294118, 0.0), (143.470588235, 0.0), (145.505882353, 0.0), (147.541176471, 0.0), (149.576470588, 0.0), (151.611764706, 0.0), (153.647058824, 0.0), (155.682352941, 0.0), (157.717647059, 0.0), (159.752941176, 0.0), (161.788235294, 0.0), (163.823529412, 0.0), (165.858823529, 0.0), (167.894117647, 0.0), (169.929411765, 0.0), (171.964705882, 0.0), (174.0, 0.0), (176.035294118, 0.0), (178.070588235, 0.0), (180.105882353, 0.0), (182.141176471, 0.01), (184.176470588, 0.0), (186.211764706, 0.0), (188.247058824, 0.0), (190.282352941, 0.0), (192.317647059, 0.0), (194.352941176, 0.0), (196.388235294, 0.0), (198.423529412, 0.0), (200.458823529, 0.0), (202.494117647, 0.0), (204.529411765, 0.0), (206.564705882, 0.0), (208.6, 0.0), (210.635294118, 0.0), (212.670588235, 0.0), (214.705882353, 0.0), (216.741176471, 0.0), (218.776470588, 0.0), (220.811764706, 0.0), (222.847058824, 0.0), (224.882352941, 0.0), (226.917647059, 0.0), (228.952941176, 0.0), (230.988235294, 0.0), (233.023529412, 0.01), (235.058823529, 0.0), (237.094117647, 0.0), (239.129411765, 0.0), (241.164705882, 0.0), (243.2, 0.0), (245.235294118, 0.0), (247.270588235, 0.0), (249.305882353, 0.0), (251.341176471, 0.0), (253.376470588, 0.0), (255.411764706, 0.0), (257.447058824, 0.0), (259.482352941, 0.0), (261.517647059, 0.0), (263.552941176, 0.0), (265.588235294, 0.0), (267.623529412, 0.0), (269.658823529, 0.0), (271.694117647, 0.0), (273.729411765, 0.0), (275.764705882, 0.0), (277.8, 0.0), (279.835294118, 0.0), (281.870588235, 0.0), (283.905882353, 0.0), (285.941176471, 0.01), (287.976470588, 0.0), (290.011764706, 0.0), (292.047058824, 0.0), (294.082352941, 0.0), (296.117647059, 0.0), (298.152941176, 0.0), (300.188235294, 0.0), (302.223529412, 0.0), (304.258823529, 0.0), (306.294117647, 0.0), (308.329411765, 0.0), (310.364705882, 0.0), (312.4, 0.0), (314.435294118, 0.0), (316.470588235, 0.0), (318.505882353, 0.0), (320.541176471, 0.0), (322.576470588, 0.0), (324.611764706, 0.0), (326.647058824, 0.0), (328.682352941, 0.0), (330.717647059, 0.0), (332.752941176, 0.0), (334.788235294, 0.0), (336.823529412, 0.0), (338.858823529, 0.01), (340.894117647, 0.0), (342.929411765, 0.0), (344.964705882, 0.0), (347.0, 0.0), (349.035294118, 0.0), (351.070588235, 0.0), (353.105882353, 0.0), (355.141176471, 0.0), (357.176470588, 0.0), (359.211764706, 0.0), (361.247058824, 0.0), (363.282352941, 0.0), (365.317647059, 0.0), (367.352941176, 0.0), (369.388235294, 0.0), (371.423529412, 0.0), (373.458823529, 0.0), (375.494117647, 0.0), (377.529411765, 0.0), (379.564705882, 0.0), (381.6, 0.0), (383.635294118, 0.0), (385.670588235, 0.0), (387.705882353, 0.0), (389.741176471, 0.0), (391.776470588, 0.01), (393.811764706, 0.0), (395.847058824, 0.0), (397.882352941, 0.0), (399.917647059, 0.0), (401.952941176, 0.0), (403.988235294, 0.0), (406.023529412, 0.0), (408.058823529, 0.0), (410.094117647, 0.0), (412.129411765, 0.0), (414.164705882, 0.0), (416.2, 0.0), (418.235294118, 0.0), (420.270588235, 0.0), (422.305882353, 0.0), (424.341176471, 0.0), (426.376470588, 0.0), (428.411764706, 0.0), (430.447058824, 0.0), (432.482352941, 0.0), (434.517647059, 0.0), (436.552941176, 0.0), (438.588235294, 0.0), (440.623529412, 0.0), (442.658823529, 0.01), (444.694117647, 0.0), (446.729411765, 0.0), (448.764705882, 0.0), (450.8, 0.0), (452.835294118, 0.0), (454.870588235, 0.0), (456.905882353, 0.0), (458.941176471, 0.0), (460.976470588, 0.0), (463.011764706, 0.0), (465.047058824, 0.0), (467.082352941, 0.0), (469.117647059, 0.0), (471.152941176, 0.0), (473.188235294, 0.0), (475.223529412, 0.0), (477.258823529, 0.0), (479.294117647, 0.0), (481.329411765, 0.0), (483.364705882, 0.0), (485.4, 0.0), (487.435294118, 0.0), (489.470588235, 0.0), (491.505882353, 0.0), (493.541176471, 0.0), (495.576470588, 0.01), (497.611764706, 0.0), (499.647058824, 0.0), (501.682352941, 0.0), (503.717647059, 0.0), (505.752941176, 0.0), (507.788235294, 0.0), (509.823529412, 0.0), (511.858823529, 0.0), (513.894117647, 0.0), (515.929411765, 0.0), (517.964705882, 0.0), (520.0, 0.0) |  | Dimensionless |  |  |
| Young_ewe_gifting_rate | 0.2/52 {HH survey data} |  | Dimensionless |  |  |
| Young_kids_mortality_rate[HHD_Gender, SR_activity_responsibility] | IF(Adult_nanny_vaccinated_becoming_recovered=0) THEN 0.6 ELSE 0 |  | Dimensionless |  |  |
| Young_ram_gifting_rate | 0.2{HH sruvey data} |  | Dimensionless |  |  |
| Young_ram_selling_'rate | 0.2{HH survey data} |  | Dimensionless |  |  |

| Run Specs | |
| --- | --- |
| Start Time | 1 |
| Stop Time | 520 |
| DT | 1/4 |
| Fractional DT | True |
| Save Interval | 0.25 |
| Sim Duration | 1.5 |
| Time Units | Weeks |
| Pause Interval | 0 |
| Integration Method | Euler |
| Keep all variable results | True |
| Run By | Run |
| Calculate loop dominance information | True |
| Exhaustive Search Threshold | 1000 |

| Array Dimension | Indexed by | Elements |
| --- | --- | --- |
| Adult_goat_price | Label (2) | Adult_billy_price Adult_nanny_price |
| Adult_sheep_price | Label (2) | Adult_ram_price Adult_ewe_price |
| Animal_age_group | Label (3) | Young Grower Adult |
| HHD_Gender | Label (2) | Male_HHD Female_HHD |
| SR_activity_responsibility | Label (2) | HHD Spouse |
| Young_goat_price | Label (2) | Young_billy_price Young_nanny_price |
| Young_sheep_price | Label (2) | Young_ram_price Young_ewe_price |

| Custom Unit | Aliases | Equation |
| --- | --- | --- |
| Lambing per lamb per Year |  | Animal/year |
| CFA |  | Money |
| Sheep per lambing |  | Sheep/ Lambing |
| Sheep per year |  | Sheep/year |
| CFA per kg |  | CFA/Kilograms |
| Kid per kidding |  | Animal/kidding |
| Goat |  | Animal/week |
| Animals per week |  | Animals/Week |
| Animals per goat |  | Animals/ goat |
| Animals per sheep |  | Animals/sheep |
| CFA per kg per week |  | CFA/(Kilograms*Week) |
| Sheep per week |  | Sheep/week |
| Kg per Animal |  | Kg/Animals |
| CFA per sheep |  | CFA/Sheep |
| CFA per goat |  | CFA/Goat |
| kg per Goat |  | Kilograms/ Goat |
| Dimensionless | dmnl unitless | 1 |
| kilowatt hours per day |  | kWh/day |
| kilowatts | kilowatt | kW |
| kg per sheep |  | kilograms/sheep |
| People per household |  | Persons/(Families *1) |
| Household |  | (Families *1) |
| Sheep per household per weeks |  | Sheep/((Families * 1) *(Weeks *1)) |
| kg per week |  | kg/week |
| kg per week per person |  | kg/(week*person) |
| People |  | Persons *1 |
| Goat per household per weeks |  | Goat/(household*Weeks) |
